# Is GCR1 the GPR157 of plants?

**DOI:** 10.1093/plphys/kiaf057

**Published:** 2025-02-21

**Authors:** Aditi Gotkhindikar, David Chakravorty, Durba Sengupta, Manali Joshi, Sarah M Assmann

**Affiliations:** Bioinformatics Centre, S. P. Pune University, Pune 411007, India; Biology Department, Pennsylvania State University, University Park, PA 16802, USA; Physical and Materials Chemistry Division, National Chemical Laboratory, Pune 411008, India; Academy of Scientific and Innovative Research (AcSIR), Ghaziabad 201002, India; Bioinformatics Centre, S. P. Pune University, Pune 411007, India; Biology Department, Pennsylvania State University, University Park, PA 16802, USA

Dear Editor,

Heterotrimeric G proteins composed of Gα and Gβγ subunits are ubiquitous across eukaryotes, including plants, animals, and yeast, and mediate multifarious signaling cascades. Whether or not plants have canonical G protein-coupled receptors (GPCRs) with 7 transmembrane domains (7TM) is an ongoing debate. We propose that the plant protein GCR1, the frontrunner as a canonical plant GPCR, is like the GPR157 protein of mammals. GPR157 is a lesser-known ciliary GPCR identified recently in humans (Takeo et al. 2016). Here, we detail the background of the debate and evidence we have compiled regarding the question, “Is GCR1 the GPR157 of plants?”.

Opisthokont GPCRs are membrane proteins with characteristic 7TM domains, known to bind diverse extracellular ligands and relay resultant signals to the cell interior via coupling with G protein subunits. GPCRs are well characterized in mammals, with around 800 GPCR loci identified in humans alone (Fredriksson et al. 2003). GPCRs in mammalian systems perceive diverse stimuli ranging from neurotransmitters to hormones to lipids to ions, and signal via G proteins to effectors including adenylyl cyclases, phospholipases, and ion channels (Hauser et al. 2017; Liu et al. 2024). In mammals, GPCRs act as guanine nucleotide exchange factors (GEFs), wherein ligand binding to the GPCR initiates conformational changes that stimulate the associated Gα subunit to release GDP and bind GTP. This results in a dissociation of Gα from the Gβγ dimer that in turn initiates downstream signaling cascades (Papasergi-Scott et al. 2024). Whether functional GPCRs of this type exist in plants is a question that is intensely debated (Assmann 2005; Urano and Jones 2013; Taddese et al. 2014). Interestingly, plant Gα subunits execute unaided rapid GDP/GTP exchange, unlike their mammalian counterparts, questioning the requirement for a GPCR-GEF in plants (Jones et al. 2011; Urano and Jones 2013). These observations have fueled the plant GPCR “existential crisis” (Chakraborty and Raghuram 2023).

The GCR1 protein from *Arabidopsis thaliana* (AtGCR1) has been the poster child of mammalian-type plant GPCRs. GCR1 has a predicted 7TM domain structure, weak sequence similarities with known GPCRs (Josefsson and Rask 1997; Plakidou-Dymock et al. 1998), and interaction with the sole canonical Arabidopsis Gα subunit, GPA1, that has previously been experimentally demonstrated by in vitro pull-down, co-immunoprecipitation, and split-ubiquitin assays (Pandey and Assmann 2004). Mutants of *gcr1* and *gpa1* both displayed abolished Phe and Tyr accumulation, and *Lhcb* upregulation, in response to blue light (Warpeha et al. 2006, 2007). Microarray analyses of *gcr1*, *gpa1*, and *gcr1gpa1* demonstrated substantial overlap in differentially expressed genes (DEGs) between the single and double mutants, with 64 DEGs shared among all 3 mutants, consistent with commonality of signaling (Chakraborty et al. 2015a, 2015b, 2019). Here, we provide support for the AtGCR1–GPA1 physical interaction using the modern split luciferase method (Fig. 1A), which as a dissociable system displays reduced false positive rates compared with other common techniques, such as BiFC, and provides the superior real-time sensitivity of a quantitative luminescence reporter (Strotmann and Stahl 2022). We also establish that the strength of interaction between GPA1 and AtGCR1 in the split-luciferase system is similar to that of GPA1 with its confirmed primary regulator, the 7TM RGS1 protein (Fig. 1A).

**Figure 1.**
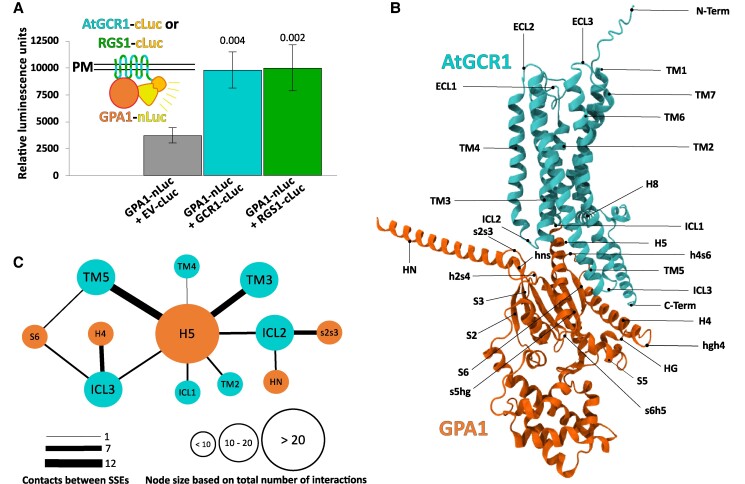
GPCR-like attributes of AtGCR1. **A)** Split luciferase (Luc) assays reveal AtGCR1 interaction with the canonical Arabidopsis Gα subunit, GPA1. The GPA1-nLuc + AtGCR1-cLuc combination was compared with a GPA1-nLuc + EV-cLuc parent vector negative control and a GPA1-nLuc + RGS1-cLuc positive control. EV, empty vector; in this case, cLuc lacking a fused partner protein. Numbers above the bars correspond to *P* values indicating statistically significant differences from the GPA1-nLuc + EV-cLuc parent vector. The assay was performed 3 independent times with similar results. Inset: diagram of the GPA1-nLuc + AtGCR1-cLuc or GPA1-nLuc + RGS1-cLuc split Luc protein–protein interaction assay, with color coding of proteins and Luc fragments consistent between the diagram and graph. See supplementary data for full details of experimental methods. **B)** Three-dimensional structural model of AtGCR1 coupled with GPA1. The proteins are rendered as cartoons. AtGCR1 is colored in cyan, while GPA1 is colored in orange. All SSEs are labeled. **C)** Interactions between SSEs of AtGCR1 and GPA1. SSEs of AtGCR1 are colored in cyan, while those of GPA1 are colored in orange. Node diameter is proportional to the number of contacts of that SSE, while edge thickness is proportional to the number of contacts between the 2 SSEs.

Recently, a structural model of AtGCR1 was proposed (Hernández et al. 2023) based on 4 GPCR templates: the Prostaglandin D2 Receptor 2, P2Y1 purinergic receptor, Beta-1 adrenergic receptor, and Prostaglandin E Receptor 3 with which AtGCR1 has 14.8% to 19.8% identity and 24.9% to 29.4% similarity, respectively, based on pairwise alignments. Here, we used the AlphaFold 3 server (Abramson et al. 2024) to build a model of AtGCR1 complexed with GPA1. We observe that AtGCR1 is predicted to display the classical 7TM structure, characteristic of GPCRs (Fig. 1B), consistent with the reports from several other groups using pre-AlphaFold computational approaches (Taddese et al. 2014; Hernández et al. 2023). Further, we analyzed the residue pairs in contact between AtGCR1 and GPA1 (Matic et al. 2023). Our results show that the AtGCR1–GPA1 structural model includes multiple interactions between TM3, TM5, and ICL2 of AtGCR1 and H5 of GPA1 (Fig. 1C). These are consistent with the secondary structure element (SSE) interactions that were reported to be central to mammalian GPCR and Gα protein subunits in a recent study analyzing G protein-coupling diversity across the GPCRome (Matic et al. 2023).

Previous comparisons of the AtGCR1 protein sequence with database information using sequence comparison programs did not provide very significant hits. To further assess the presence of homologs, we performed sequence comparison of AtGCR1 with the nonredundant protein sequence database (as of August 30, 2024) using the BLASTp algorithm with vertebrates as the organism filter. We found the top hits to be against sequences annotated as “uncharacterized proteins,” “cyclic AMP receptor-like proteins,” and “G protein-coupled receptor 1-like proteins” in amphibian and fish species, with the most significant *E*-value being 4.00*E*−19. However, aside from the homology to cyclic AMP receptors, which had already been reported between AtGCR1 and *Dictyostelium* cyclic AMP receptors (Taddese et al. 2014; Chakraborty and Raghuram 2023), new functional information on these hits was lacking, so we next restricted the search to mammals. To our surprise, the top hits are the GPR157 proteins from various *Camelu*s species with a query coverage of 51%, 28.1% identity, and a low *E*-value range of 2.00*E*−9 to 7.00*E*−10 (Supplementary Table S1-A). Pairwise alignment with the GPR157 isoform X2 of *Camelus ferus* reveals 23.7% identity and 41.5% similarity over the entire sequence lengths (Supplementary Table S1-B). The next set of hits from the BLASTp results are the GPR157 proteins from *Vicugna, Chrysochloris*, and *Myodes* species.

The sequence comparison values we found with mammalian proteins are higher than any reported to date. To investigate the comparison with GPR157 further, we compiled ∼400 protein sequences that are complete and annotated as GPR157 from UniprotKB and performed a multiple sequence alignment of AtGCR1 with these sequences (Supplementary Fig. S1). The multiple sequence alignment and 7TM predictions for a few representative proteins were used to map helical boundaries of all GPR157 proteins aligned with AtGCR1. Of the predicted 7TM helices, more than half of the amino acid residues in the 3rd helix are conserved (identical) or semiconserved (amino acids with similar properties) and around a third of the 2nd, 4th, 5th, and 6th helices are conserved/semiconserved (Supplementary Fig. S1). The conservation improves if the AtGCR1 sequence is compared with the consensus sequence of GPR157 (Fig. 2, A and B). It is noteworthy that the 3rd TM helix of AtGCR1 is one of the most conserved, since this helix is known to be the structural and functional hub of GPCRs (Venkatakrishnan et al. 2013). Taddese et al. (2014) had aligned TM Helix 3 to each of the GPCR classes to conclude that AtGCR1 is similar to Class A, B, and E GPCRs. GPCRs are known to possess low overall sequence identities (particularly because of very low conservation in the loop regions as is also seen in Fig. 2A), but high structural conservation.

**Figure 2.**
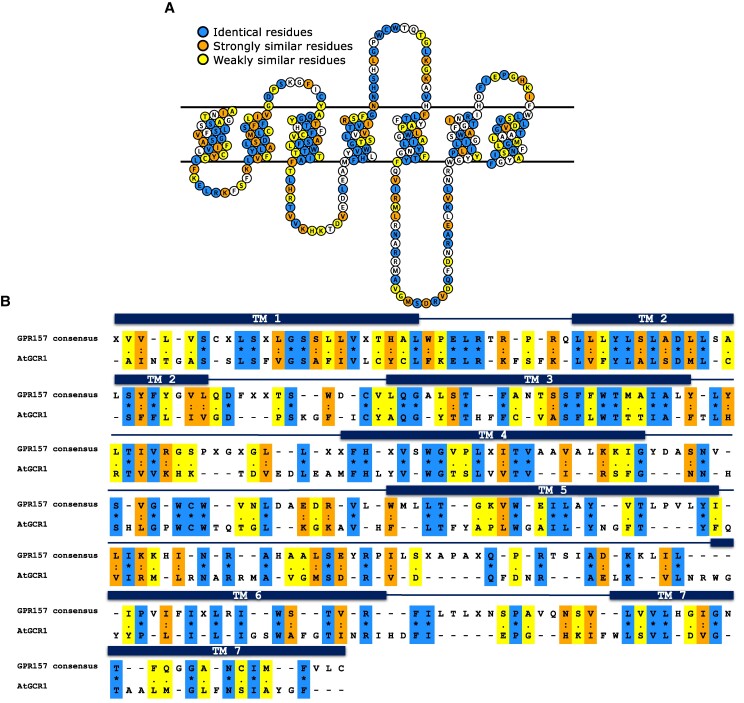
Comparison of AtGCR1 with GPR157. **A)** AtGCR1 sequence represented as a snake plot. Residues colored in blue are identical between AtGCR1 and the consensus sequence of GPR157, while those residues colored in orange are strongly similar and those residues colored in yellow are weakly similar. **B)** Sequence alignment of AtGCR1 with the consensus sequence of GPR157. The N- and C-terminal residues are not shown in **A)** and **B)** for clarity.

To assess the extent of AtGCR1 similarity with any other mammalian GPRs (i.e. annotated orphan GPCRs), a BLASTp comparison was performed using the protein sequence of AtGCR1 and all mammalian GPRs (annotated as such) in the UniprotKB. While the vast majority of hits corresponded to GPR157 proteins from various mammals, BLASTp indicates limited local similarity of AtGCR1 with GPR112, GPR126, GPR125, GPR124, and GPR110. Of these, only GPR110 shows appreciable full-length similarity, which at 16.8%/26.7% identity/similarity is considerably less than for human GPR157, with 24.0%/39.1% identity/similarity (Supplementary Table S2). To evaluate the similarity between a broad selection of plant GCR1 sequences with mammalian GPR157 protein sequences, the GCR1 sequences of 10 model plant species (Supplementary Table S3) were compared with the 12 mammalian GPR157 sequences obtained from BLASTp results with AtGCR1 as query. The results indicate that, like AtGCR1, the GCR1 sequences of other plant species show similarity to mammalian GPR157 sequences (Supplementary Fig. S2). The match to GPR157 by bioinformatics analysis is thought provoking and raises the question as to whether GCR1 could function in plants similarly as GPR157 does in mammals.

GPR157 is reported to have sequence similarities to Class A, Class B, and slime mold cyclic AMP receptors, uncannily similar to that reported for AtGCR1 in the early literature (Taddese et al. 2014; Chakraborty and Raghuram. 2023). Recently, GPR157 was reported to be expressed in the primary cilia of radial glial progenitor cells, where it contributes to their neuronal differentiation (Takeo et al. 2016). Assessment of GPR157 activation indicated that the ligand is in the cerebrospinal fluid, but its identity remains to be discovered; hence GPR157 remains an orphan GPCR. Peptides corresponding to the C-terminal region of Gαq were used as competitive blockers to determine that GPR157 couples to the Gαq class of G proteins (Takeo et al. 2016). Overexpression of GPR157 increased cytosolic Ca^2+^ concentrations, while coexpression of an InsP3 trapping protein suppressed this increase. Thus, it was concluded that GPR157 couples with Gαq to induce Ca^2+^ signaling via InsP3.

GPA1 has been reported to be similar to mammalian Gαi1 and Gαo based on sequence similarity (Ma et al. 1990; Gookin et al. 2023); GPA1 also displays similarity to the Gαq family (Supplementary Fig. S3). Knockout analyses have shown that plant G proteins regulate stomatal apertures, and stomatal closure is triggered by experimental elevation of cytosolic Ca^2+^ or InsP3 (Gilroy et al. 1990). Moreover, an early study found that overexpression of either AtGCR1 or GPA1 in Tobacco BY2 cells elevated PLC activity and InsP3 production (Apone at al. 2003). Thus, the possibility arises that, analogous to GPR157-Gαq, GCR1-GPA1 functions to regulate PLC activity, InsP3 production, and Ca^2+^ signaling.

In summary, AtGCR1 and related plant orthologs have not yet been shown to be anything other than GPCRs; AtGCR1 interacts with GPA1, and InsP3 production and Ca^2+^ signaling have been linked to plant G proteins based on transgenic analyses. We accordingly propose that GCR1 might function like mammalian GPR157, coupling with G proteins and regulating Ca^2+^ signaling in plants. Identification of an agonist for GCR1 and determination of the impacts of ligand binding on plant Gα conformation are ultimately needed to lay to rest the debate regarding plant 7TM GPCRs.

## Supplementary Material

kiaf057_Supplementary_Data

## Data Availability

The data supporting the findings of this study have been provided in the text and in the supplementary data files and are available upon request.

## Supplementary data

The following materials are available in the online version of this article.

**Supplementary Figure S1.** Multiple sequence alignment of proteins with “GPR157” in the annotation, aligned with AtGCR1 (top row), performed with MUSCLE software (Edgar 2004). Helix boundaries were predicted for a few representative sequences using DeepTMHMM program (Hallgren et al. 2022). Only the helices are included in the alignment for clarity. The amino acid residues are colored according to the helices they belong to, red—helix1, blue—helix2, green—helix3, purple—helix4, orange—helix5, brown—helix6, and magenta—helix7. The conserved positions are shown in the last row. The fully conserved residues are indicated by “*”, residues with strongly similar properties are indicated by “:”, and residues with weakly similar properties are indicated by “.”.

**Supplementary Figure S2.** Matrices comparing the **A)** percent identity and **B)** percent similarity values between AtGCR1 orthologs from a broad selection of plant species vs. mammalian GPR157 protein sequences. Aside from the reference AtGCR1 sequence in the top row, plant AtGCR1 orthologs were ordered based on the maximum value in the row of identities, and the same order was maintained for the similarity matrix. Mammalian GPR157 sequences on the *x* axis are listed in the same order as in Supplementary Table S1-B. Accession IDs of all plant GCR1 protein sequences are provided.

**Supplementary Figure S3.** Matrix with background color coding and enumeration of percent identity (cyan) and percent similarity (salmon) values between GPA1 of *A. thaliana* and the canonical isoforms of each of the 16 human Gα proteins. Human Gα proteins are grouped into 4 families: Gαi (GNAI1, GNAI2, GNAI3, GNAO1, GNAT1, GNAT2, GNAT3, and GNAZ), Gαq (GNAQ, GNA11, GNA14, and GNA15), Gαs (GNAS and GNAL), and Gα12/13 (GNA12 and GNA13). GPA1 displays the highest identity and similarity with the Gαi and Gαq families of human Gα subunits.

**Supplementary Table S1-A.** Sequence comparison results of AtGCR1 with nonredundant (nr) mammalian protein sequences using BLASTp (Altschul et al. 1990) search tool.

**Supplementary Table S1-B.** Pairwise sequence alignment of AtGCR1 with representative GPR157 protein sequences.

**Supplementary Table S2.** Limited sequence homology between AtGCR1 and GPR proteins other than GPR157. Homology between human GPR157 and AtGCR1 is included as a reference for comparison of the next most similar set of mammalian GPR proteins to AtGCR1.

**Supplementary Table S3.** Model plant species with ≥2 chromosome-level assemblies that were chosen for comparison of AtGCR1 orthologs with GPR157 protein sequences.

## Dive Curated Terms

The following phenotypic, genotypic, and functional terms are of significance to the work described in this paper:

GCR1 Gramene: AT1G48270
GCR1 Araport: AT1G48270

RGS1 Gramene: AT3G26090
RGS1 Araport: AT3G26090

GDP CHEBI: CHEBI:17552
