# Supplementary material for: Is GCR1 the GPR157 of plants?
Source: Plant Physiol. 2025 Feb 21;197(2):kiaf057. doi: 10.1093/plphys/kiaf057 (PMC11843928; doi:10.1093/plphys/kiaf057)
Supplement: kiaf057_Supplementary_Data [file kiaf057_supplementary_data.pdf]

Supplemental Data

Is GCR1 the GPR157 of Plants?

Aditi Gotkhindikar[1], David Chakravorty[2], Durba Sengupta[3,4], Manali Joshi[1*], Sarah M. Assmann[2*]

1. Bioinformatics Centre, S. P. Pune University, Pune 411007
2. Biology Department, Pennsylvania State University, University Park, Pennsylvania 16802
3. Physical and Materials Chemistry Division, National Chemical Laboratory, Pune 411008
4. Academy of Scientific and Innovative Research (AcSIR), Ghaziabad, India

*Authors for correspondence
manalijoshi@unipune.ac.in, sma3@psu.edu

Supplemental Table S1-A: Sequence comparison results of AtGCR1 with non-redundant (nr) mammalian protein sequences using BLASTp (Altschul et al. 1990) search tool.

Description	Scientific Name	Max Score	Total Score	Query Coverage (%)	E value	Percent Identity (%)	Accession Length	Accession ID
G-protein coupled receptor 157	*Camelus dromedarius*	67.4	67.4	51	7.00e-10	28.09	316	KAB1268120.1
G-protein coupled receptor 157	*Camelus bactrianus*	66.2	66.2	51	2.00e-09	28.09	332	XP_010955888.1
G-protein coupled receptor 157 isoform X1	*Camelus dromedarius*	66.2	66.2	51	2.00e-09	28.09	419	XP_064350157.1
G-protein coupled receptor 157	*Camelus dromedarius*	65.9	65.9	51	3.00e-09	28.09	366	KAB1268121.1
G-protein coupled receptor 157 isoform X2	*Camelus dromedarius*	65.5	65.5	51	3.00e-09	28.09	331	XP_031319861.2
G-protein coupled receptor 157 isoform X2	*Camelus ferus*	65.1	65.1	51	4.00e-09	28.09	331	XP_032351017.1
G-protein coupled receptor 157	*Vicugna pacos*	45.8	45.8	30	0.011	29.52	435	XP_031539442.1
PREDICTED: probable G-protein coupled receptor 157	*Chrysochloris asiatica*	45.1	45.1	30	0.016	30.48	334	XP_006873156.1
G-protein coupled receptor 157	*Myodes glareolus*	44.3	44.3	28	0.024	30.00	332	XP_048271334.1

Supplemental Table S1-B: Pairwise sequence alignment of AtGCR1 with representative GPR157 protein sequences

Accession ID	Protein	Species	Percent Identity (%)	Percent similarity (%)
KAB1268121.1	G-protein coupled receptor 157	*Camelus dromedarius*	22.4	38.8
KAB1268120.1	G-protein coupled receptor 157	*Camelus dromedarius*	21.2	37.0
XP_031319861.2	G-protein coupled receptor 157 isoform X2	*Camelus dromedarius*	23.7	41.3
XP_064350157.1	G-protein coupled receptor 157 isoform X1	*Camelus dromedarius*	19.0	32.0
XP_010955888.1	G-protein coupled receptor 157	*Camelus bactrianus*	23.6	41.4
XP_032351017.1	G-protein coupled receptor 157 isoform X2	*Camelus ferus*	23.7	41.5
AAI17997.1	G-protein coupled receptor 157	*Mus musculus*	23.1	39.4
AAV35060.1	G-protein coupled receptor 157	*Homo sapiens*	24.0	39.1
XP_048271334.1	G-protein coupled receptor 157	*Myodes glareolus*	22.2	37.1
NP_001012107.1	G-protein coupled receptor 157	*Rattus norvegicus*	21.1	36.6
XP_006873156.1	PREDICTED: probable G-protein coupled receptor 157	*Chrysochloris asiatica*	20.1	34.4
XP_031539442.1	G-protein coupled receptor 157	*Vicugna pacos*	18.1	31.0

Supplemental Table S2: Limited sequence homology between AtGCR1and GPR proteins other than GPR157. Homology between human GPR157 and AtGCR1 is included as a reference for comparison of the next most similar set of mammalian GPR proteins to AtGCR1.

Sr. No.	Protein	UniprotKB ID	Sequence Length	Alignment Length	Percent Identity (%)	Percent Similarity (%)
**Ref**	**GPR157 *Homo sapiens***	**Q5UAW9**	**335**	**363**	**24.0**	**39.1**
1	GPR112 (Adhesion G-protein coupled receptor G4)	Q8IZF6	3080	3134	2.2	4.2
2	GPR126 (Adhesion G-protein coupled receptor G6)	Q86SQ4	1221	1272	5.4	10.3
3	GPR125 (Probable G-protein coupled receptor 125)	A0A9B0GNW0	1359	1375	6.0	10.0
4	GPR124 (G-protein coupled receptor 124)	A0A340WFL1	1276	1320	5.6	9.5
5	GPR110 (G protein-coupled receptor 110)	A6JJ42	460	505	16.8	26.7

Supplemental Table S3: Model plant species with ≥ two chromosome level assemblies that were chosen for comparison of AtGCR1 orthologs with GPR157 protein sequences.

Name of plant species	Number of genomes available in public domain	Assembly level				URL
		Chromosome	Contig	Scaffold	Complete	
Brachypodium distachyon as a grass (monocot) model	5	2	3	0	0	https://www.ncbi.nlm.nih.gov/datasets/genome/?taxon=15368
Physcomitrium patens as a model for mosses	3	2	0	0	1	https://www.ncbi.nlm.nih.gov/datasets/genome/?taxon=3218
Medicago truncatula as a model for legumes	6	2	0	4	0	https://www.ncbi.nlm.nih.gov/datasets/genome/?taxon=3880
Populus trichocarpa as a model for trees	4	2	0	2	0	https://www.ncbi.nlm.nih.gov/datasets/genome/?taxon=3694
Marchantia polymorpha as a model for land plant evolution	9	3	4	2	0	https://www.ncbi.nlm.nih.gov/datasets/genome/?taxon=3197
Setaria viridis as a model for C4 photosynthesis	3	3	0	0	0	https://www.ncbi.nlm.nih.gov/datasets/genome/?taxon=4556
Phragmites australis as a model for invasive plants	4	2	1	1	0	https://www.ncbi.nlm.nih.gov/datasets/genome/?taxon=29695
Eutrema salsugineum as a model for salt tolerance	3	2	0	1	0	https://www.ncbi.nlm.nih.gov/datasets/genome/?taxon=72664
Pisum sativum as a model for legume crops	9	5	2	2	0	https://www.ncbi.nlm.nih.gov/datasets/genome/?taxon=3888
Oryza sativa as the first sequenced crop species	114	49	38	23	4	https://www.ncbi.nlm.nih.gov/datasets/genome/?taxon=4530

```
sp|O04714|GCR1_ARATH           ----AINTGASSL----SFVGSAFIVLCYCLLVFYLALSDMLCS---FFLIV-AQGYTTHFFCVASFLWTTTIAF---M-FHLYVWGTSLVVTV----IRSFG---FLTFYAPLWGAILYNGFTYF--WGYYPLILIGSWAFGTINRI-WLSVLD--VGTAALMGLFNSIAYGF-----
tr|A0A8J7NNB6|A0A8J7NNB6_ATRSP VVILT----SCLL----SFLGSCLIIVTYVALLVFLSAADLLSAVSYFYGVLVLQGAVSTFANTSSFFWTVAIAVLVLF-FHIISWGVPLGITVAAVAL----VLWML-LTGKIWELLAYLALPVLYVLTLIPIIFIALRVWSTVRFFLVLVTLH------------------VSTSPG
tr|A0A8C4RYT6|A0A8C4RYT6_ERPCA VLILV----SCLL----SFLGSCVIILTYVLLLVFLSVTDLLSALSYFYGVMVIQGAVSTFANTSSFFWTVSVALLLW--FHILSWGLPLGITVAAVSL----ILWML-LTGKLWEIVAYLILPLLYLLTLIPLIFIFLRIWSTIRFFLGLMILH-GIGN-TFQGGANCIMFVLFTQVV
tr|A0A8X8BMG4|A0A8X8BMG4_POLSE VLILV----SCLL----SFLGSCVIICTYVLLLVFLSVTDLLSALSYFYGVLVIQGAVSTFANTSSFFWTVSVALLLW--FHILSWGVPLGITVAAVAL----ILWML-LTGKLWEIMAYLILPLLYLLTLIPVIFIFLRIWSTIRFFLVLMILH-GIGN-TFQGGANCIMFVLFTQVV
tr|A0A0P7UVM4|A0A0P7UVM4_SCLFO AAVLL----SCAL----SVLGSALIIILTYAALLVYLSVTDLLSALAFSYGAWVVQGALSTLANTSSFFWTVAIAVLVSW-FHLVSWGLPLGITVAAVSL----VLWIL-LTGKIWEFLAYLILPVLYIMTLIPIIFIGLRIWSTLRFLLVLVTLH-GIGN-TFQGAANCTMFVLFTKVV
tr|A0A8C4C413|A0A8C4C413_9TELE VATLI----SCGL----SVLGSLLIIILTYALLLAFLSIADLLSAGSYFYGACVAQGAISTFSNTSSFFWTVAIALLVQY-FHLVSWGVPLGITVAAVAL----VAWML-LTGKVWEILAYFTLPVLYILTLIPIIFIFLRMWSTIRFILVLVTLH-GIGN-TFQGAANCIMFVLFTQPI
tr|A0A8U1EU97|A0A8U1EU97_SALNM IVILI----SCAL----SFLGSLLIICTYIILLVYLSVADFLSAGSYAYGVWVVQGAISTFANTSSFFWTVAIAIFVLC-FHVISWGLPLGITVAAVSL----VLWML-LTGKIWEFLAYLTLPVLYVLTLIPIIFIALRIWSTVRFLLVLVTLH-GIGN-TFQGAANCIMFVLFTQPI
tr|A0A1S3MIQ3|A0A1S3MIQ3_SALSA IVILI----SCAL----SFLGSLLIICTYIILLVYLSVADFLSAGSYAYGVWVVQGAISTFANTSSFFWTVAIAIFVLC-FHVISWGVPLGITVAAVAL----VLWML-LTGKIWEFLAYLTLPVLYVLTLIPIIFIALRIWSTVRFLLVLVTLH-GIGN-TFQGAANCIMFVLFTQPI
tr|A0A673Y6F4|A0A673Y6F4_SALTR IVILI----SCAL----SFLGSLLIICTYIILLVYLSVADFLSAGSYAYGVWVVQGAISTFANTSSFFWTVAIAIFVLC-FHVISWGLPLGITVAAVSL----VLWML-LTGKIWEFLAYLTLPVLYVLTLIPIIFIALRIWSTVRFLLVLVTLH-GIGN-TFQGAANCIMFVLFTQPI
tr|A0A8C7FGQ3|A0A8C7FGQ3_ONCKI IVILI----SCAL----SFLGSLLIIFTYIILLVYLSVADLLSAGSYAYGVWVVQGAISTFSNTSSFFWTVAIAIFVLC-FHVISWGVPLGITVAAVAL----VLWML-LTGKIWEFLAYLTLPVLYVLTLIPIIFIALRIWSTVRFLLVLVTLH-GIGN-TFQGAANCIMFVLFTQPI
tr|A0A8C7LRT9|A0A8C7LRT9_ONCMY IVILI----SCAL----SFLGSLLIIFTYIILLVYLSVADLLSAGSYAYGVWVVQGAISTFANTSSFFWTVAIAIFVLC-FHVISWGLPLGITVAAVSL----VLWML-LTGKIWEFLAYLTLPVLYVLTLIPIIFIALRIWSTVRFLLVLVTLH-GIGN-TFQGAANCIMFVLFTQPI
tr|A0A8J4XDN7|A0A8J4XDN7_CLAMG VVVLF----SCVL----SALGSALVMGTYALLLVFLSVADLLSAVSYAYGVYVTQGAVSTFSNTSSFFWTVAIAILVLY-FHLISWGVPLGITVAAVAL----VMWML-LTGKIWELLAYLILPVLYILTLIPIIFIALRIWSTVRFVLVLVILH-GIGN-TFQGAANCIMFVLCTRPV
tr|A0A2D0SJZ1|A0A2D0SJZ1_ICTPU VVVLL----SCAL----SALGSVLVVSTYAKLLVFLSVADFLSAVSYSYGVYVTQGAISTFSNTSSFFWTVAIAVLVLY-FHLISWGLPLGITVAAVSL----VLWML-LTGKIWELLAYVILPLLYILTLIPIIFIALRIWSTVRFVLVLVTLH-GIGN-TFQGAANCIMFVLCTRPV
tr|W5UER8|W5UER8_ICTPU         VVVLL----SCAL----SALGSVLVVSTYAKLLVFLSVADFLSAVSYSYGVYVTQGAISTFSNTSSFFWTVAIAVLVLY-FHLISWGVPLGITVAAVAL----VLWML-LTGKIWELLAYVILPLLYILTLIPIIFIALRIWSTVRFVLVLVTLH-GIGN-TFQGAANCIMFVLCTRPV
tr|Q498W7|Q498W7_DANRE         VVILT----SCVL----SFFGSLLIISTYVSLLVYLSVSDRLSALSYFYGVCIAQGAVSTFANTSSFFWTVAIAILVLY-FHLISWGLPLGITVAAVSL----VLWML-LTGKIWEFIAYVTLPVLYILTLIPIIFIMLRVWSTVRFLLVLVTLH-GIGN-TFQGAANCIMFVLFTPSI
tr|A0A5C1D729|A0A5C1D729_CTEID VVILA----SCIL----SFFGSLLIICTYVHLLVYLSVSDLLSALSYFYGVVIAQGAISTFANTSSFFWTVAIAILVLY-FHLISWGVPLGITVAAVAL----VLWML-LTGKIWEFIAYVTLPVLYILTLIPIIFIMLRMWSTIRFLLVLVTLH-GIGN-TFQGAANCIMFVLLTPSI
tr|A0A9Q9XQD4|A0A9Q9XQD4_CYPCA VVILT----SCVL----SFFGSLLIICTYAKLLMYLSVTDLLSALSYFYGVLIAQGAISTFANTSSFFWTVAIAILVLY-FHLISWGLPLGITVAAVSL----VLWML-LTGKIWEFIAYVTLPVLYILTLIPIIFIMLRMWSTIRFLLVLVTLH-GIGN-TFQGAANCIMFVLLTPSI
tr|A0A8B9HN74|A0A8B9HN74_ASTMX VVILL----SCVF----SFLGSLLIICTYATLLVFLSVSDLLSALSYFYGVLIAQGAISTFANTSSFFWTVAIAILVLY-FHLISWGVPLGITVAAVAL----VLWML-LTGKIWEFLAYLTLPVLYILTLIPIIFIALRMWSTVRFLLVLVILH-GIGN-TFQGAANCIMFVLCTRPV
tr|A0A4W4G075|A0A4W4G075_ELEEL VVILM----SCAV----SFLGSLLIICTYATLLVFLSVTDLLSAASYLYGVWVVQGAISTFANTSSFFWTVAIAILVLY-FHLISWGLPLGITVAAVSL----VLWML-LTGKIWEFLAYVTLPVLYALTLIPIIFIVLRVWSTVRFLLVLVTLH-GVGN-TFQGAANCIMFTLCTRAV
tr|A0A8C5I0P8|A0A8C5I0P8_9TELE VVVLC----SCVL----SLVGSTLIITTFVLLLVYLSVSDWLSAASYAFGVWVVQGALSTFSNTSSFFWTVAIAVLVLY-FHLISWGVPLGITVAAVAL----ILWML-LTGKIWEFLAYLTLPILYILTLIPVIFILLRIWSTVRFILVLVTLH-GIGN-TSQGAANCIMFVLFTRPI
tr|H2LMC9|H2LMC9_ORYLA         VVVLC----TCVL----SLAGSTLIFLTFVLLLVYLSVSDWLSAASYAYGVGVAQGAISTFANTSSFFWTVTIAVLVLS-FHLVSWGLPLGITVAAVSL----ILWML-LTGKIWEFLAYLTLPVLYILTLIPIIFIFLRIWSTVRFILALVMLH-GIGN-TSQGAANCIMFVLFTQPI
tr|A0A6J2V3A0|A0A6J2V3A0_CHACN IVILI----SCAL----SLLGSLLIICTYIILLVFLSVTDLLSALSYSYGVGITQGAISTFANTSSFFWTVAIAVLVLY-FHLISWGVPLGITVAAVAL----VLWML-LTGKIWEILAYVILPVLYILTLIPIIFISLRIWSTVRFLLVLVTLH-GIGN-TFQGAANCIMFVLCTKPV
tr|A0A6P3VVK8|A0A6P3VVK8_CLUHA VVILI----SCAL----SFLGSLLIIITYIILLVFMSVADLLSAISYSFGVWIVQGAISTFANTSSFFWTVAIAILVLY-FHLVSWGLPLGITVAAVSL----VLWML-LTGKIWELLAYVMLPVLYLLTLIPIIFIFLRIWSTVRFILVLVTLH-GVGN-TFQGAANCIMFVLCTQPV
tr|A0A665UGT5|A0A665UGT5_ECHNA VVVLV----SCGL----SLAGSSLIIVTFLLLLVYLSLSDWLSAASYAFGVWVVQGSVSTFANTSSFFWTVSIAVLEPL-FHLVSWGVPLGITVAAVAL----VLWML-LTGKIWEFLAYLILPVLYVLTLIPVIFIALRIWSTVRFILVLVTLH-GIGN-TSQGAANCIMFVLFTQPL
tr|I3J2G8|I3J2G8_ORENI         VIVLL----SCAL----SFLGSSLIILTYIILLVYLSVSDWLSAVSYAFGVWVAQGAISTFANTSSFFWTVAIAVLVLV-FHLVSWGLPLGITVAAVSL----VLWML-LTGKIWEFLAYLTLPFLYILTLIPIIFILLRIWSTVRFILALVTLH-GIGN-TLQGAANCVMFVLFTQPI
tr|A0A6P7MXH1|A0A6P7MXH1_BETSP VVVLL----SCAL----SFVGSSLILLTYCVLLVFLSVADWLSAVSYAFGVWVVQGAISTFANTSSFFWTVAIAVLVLI-FHVVSWGVPLGITVAAVAL----VLWML-LTGKIWEFLAYITLPVLYILTLIPIMFIILRIWSTVRFVLVLVTLH-GIGN-TLQGAANCIMFVLCTQPI
tr|A0A672FLS9|A0A672FLS9_SALFA VVVLC----SCAL----SFLGSTLIIATYILLLVYLSVADWLSAVSYAYGAWVAQGAVSTFSNTSSFFWTVAIAVLVLP-FHLVSWGLPLGITVAAVSL----VLWML-LTGKIWEFLAYVTLPVLYVLTLIPIIFIALRMWSTVRFVLVLVTLH-GIGN-TSQGAANCIMFVLFTRPI
tr|A0A3B5K5F4|A0A3B5K5F4_TAKRU VVVLF----TCAL----SFVGSSLIILTFIVLLVYLSVSDWLSAVSYAFGVWTIQGAISTFANSSSFFWTVAIAVLVLF-FHLVSWGVPLGITVAAVAL----VLWML-LTGKLWEFMAYLTLPVLYILTLIPIIFIALRIWSTVRFVLVLVTLH-GIGN-TSQGAANCIMFVLLTGPI
tr|A0A672YWH8|A0A672YWH8_9TELE VVVLC----SCGL----SFVGSTLIILTYIMLLVFLSVSDWLSAVSYAFGVWVVQGAVSTFANTSSFFWTVAIAVLVLF-FHLISWGLPLGITVAAVSL----VLWML-LTGKIWEFLAYLTLPVLYILTLIPIIFIILRIWSTVRFILVLVTLH-GIGN-TSQGAANCIMFVLFTQPV
tr|A0A6P7IUZ2|A0A6P7IUZ2_9TELE VVVLC----SCAL----SFLGSSLIVLTFIILLVFLSVADWLSAVSYAFGVWVVQGAISTFANTSSFFWTVAIAVLVLF-FHLVSWGVPLGITVAAVAL----VLWML-LTGKIWEFLAYLTLPILYILTLIPIIFIVLRIWSTVRFILVLVTLH-GIGN-TSQGAANCIMFVLFTQPI
tr|A0A671TMK6|A0A671TMK6_SPAAU VIVLF----SCAL----SFLGSSLIILTYIILLVYLSVSDWLSAVSYAFGAWIVQGAISTFANTSSFFWTVAIAVLVLF-FHLVSWGLPLGITVAAVSL----VLWML-LTGKIWEFMAYLTLPVLYILTLIPIIFIVLRIWSTVRFILLLVTLH-GIGN-TSQGAANCIMFVLCTQPI
tr|A0A2U9BZ00|A0A2U9BZ00_SCOMX VVVLC----SCAL----SFVGSSLIILTYALLLVFLSVSDWLSAVSYAFGVWVAQGAISTFANTSSFFWTVAIAVLVLV-FHLVSWGVPLGITVAAVAL----VLWML-LTGKIWEFLAYLTLPVLYVLTLIPIIFIVLRIWSTVRFILVLVTLH-GIGN-TSQGAANCIMFVLFTRPI
tr|A0A4Z2IYT7|A0A4Z2IYT7_9TELE VVVLC----SCAL----SFVGSSLIILTYVMLLVYLSVADWLSAVSYAVGVWVAQGAISTFANTSSFFWTVAIAVLVWV-FHLVSWGLPLGITVAAVSL----VLWML-LTGKIWEFLAYLTLPVLYILTLIPIIFIVLRIWSTVRFILVLVTLH-GIGN-TSQGAANCIMFVLLTQPI
tr|A0A8C9X3U2|A0A8C9X3U2_SANLU VVVLC----SCGL----SFLGSSLIIFTYIILLVYLSISDWLSAVSYAFGVWVVQGAISTFANTSSFFWTVAIAVLVLF-FHLVSWGVPLGITVAAVAL----VLWML-LTGKIWEFLAYLTLPVLYILTLIPIIFIVLRIWSTVRFILVLVTLH-GIGN-TSQGAANCIMFVLLTRPI
tr|A0A6J2Q3Q3|A0A6J2Q3Q3_COTGO VVVLC----SCAL----SFLGSSLIILTYIILLVFLSVSDWLSAVSYAFGVWIAQGAVSTFANTSSFFWTVAIAVLVLF-FHFVSWGLPLGITVAAVSL----VLWML-LTGKIWEFLAYLTLPVLYILTLIPIIFIVLRIWSTVRFILVLVTLH-GIGN-TSQGAANCIMFVLLTRPI
tr|A0A6I9PDZ1|A0A6I9PDZ1_9TELE VVVLC----SCAL----SFLGSSLIILTYIILLVFLSVSDWLSAVSYAYGVWIAQGAVSTFANTSSFFWTVAIAVLVWC-FHIVSWGVPLGITVAAVAL----ILWML-LTGKIWEFLAYLTLPVLYILTLIPIIFIVLRIWSTVRFILVLVTLH-GIGN-TSQGAANCIMFVLLTRPL
tr|A0A6P8UEH0|A0A6P8UEH0_GYMAC VVVLC----SCAL----SFLGSSLIILTYIILLVFLSVSDWLSAVSYAYGVWIAQGAVSTFANTSSFFWTVAIAVLVWC-FHIVSWGLPLGITVAAVSL----ILWML-LTGKIWEFLAYLTLPVLYILTLIPIIFIVLRIWSTVRFILVLVTLH-GIGN-TSQGAANCIMFVLLTRPL
tr|A0A2I4C719|A0A2I4C719_AUSLI VVVLC----TCAL----SFLGSSLIVLTYLVLLVYLSVTDWLSAVSYAVGVWVVQGAVSTFANTSSFFWTVAIAVLVLS-FHLVSWGVPLGITVAAVAL----VLWML-LTGKIWEFLAYLTLPVLYILTLIPIIFIILRIWSTVRFVLVLVTLH-GIGN-TSQGAANCIMFVLFTQPI
tr|A0A1A7W719|A0A1A7W719_9TELE VVVLC----TCVL----SFLGSSLIIITYIILLVYLSVTDLLSAVSYAYGVWVVQGAISTFANTSSFFWTVAIALLVLL-FHLVSWGLPLGITVAAVSL----VLWML-LTGKIWEFLAYLTLPVLYILTLIPIIFIFLRIWSTVRFLLVLVTLH-GIGN-TSQGAANCIMFVLFTQPV
tr|A0A9D2XXV1|A0A9D2XXV1_NOTFU VVVLC----TCVL----SFLGSSLIIITYIILLAYLSVTDLLSAVSYAYGVWVVQGAISTFANTSSFFWTVAIAVLVTL-FHLVSWGVPLGITVAAVAL----VLWML-LTGKIWEFLAYLTLPVLYILTLIPIIFIFLRIWSTVRFLLVLVTLH-GIGN-TSQGAANCIMFVLFTQPI
tr|A0A1A8J6J0|A0A1A8J6J0_NOTKU VVVLC----TCVL----SFLGSSLIIITYIILLAYLSVTDLLSAVSYAYGVWVVQGAISTFANTSSFFWTVAIAVLVTL-FHLVSWGLPLGITVAAVSL----VLWML-LTGKIWEFLAYLTLPVLYILTLIPIIFIFLRIWSTVRFLLVLVTLH-GIGN-TSQGAANCIMFVLFTQPI
tr|A0A4W3GN33|A0A4W3GN33_CALMI VIISI----SCCC----SFLGSTLIIATHGLLLVYLSVADLLSAVSYLYGVLVLQGALSTFANTSSFFWTVAIAISLCVLFHAVSWGVPLGITVAAVAL----FLWML-LTGKIWEFIAYITLPILYILTLIPIIFILLRIWSTVRFILLLVTLH-GVGN-TFQGAANCIMFILCTKPI
tr|A0A974CG21|A0A974CG21_XENLA VIVLL----SCVL----SFLGSALIIATYIFLLLFLSVADLLSAISYFYGVLVTQGAISTFSNTSSFFWTVAVAVIPW--LHLISWGLPLGITVAAVSL----LLWML-LAGKAWEILAYLTLPVLYILVFIPVIFIFLRVWSTIRFILILVTLH-GIGN-TFQGGANCIMFVLCTPVI
tr|Q6GQ05|Q6GQ05_XENLA         VIVLL----SCVL----SFLGSALIIATYIFLLLFLSVADLLSAISYFYGVLVTQGAISTFSNTSSFFWTVAVAVIPW--LHLISWGVPLGITVAAVAL----LLWML-LAGKAWEILAYLTLPVLYILVFIPVIFIFLRVWSTIRFILILVTLH-GIGN-TFQGGANCIMFVLCTPVI
tr|B4F750|B4F750_XENTR         VIVLL----SCVL----SFLGSALIIVTYILLLLFLSVADLLSALSYFYGVLVTQGAISTFSNTSSFFWTVAVAVIPW--LHLISWGLPLGITVAAVSL----LLWML-LAGKVWEILAYLILPVLYILVFIPIIFIFLRVWSTIRFILILVTLH-GIGN-TFQGGANCIMFVLCTPVI
tr|A0A8C5MKY8|A0A8C5MKY8_9ANUR VIVLL----SCAL----SFLGSVLIIFTHIRLLLFLSVADLLSAGSYFYGVLVAQGAVSTFSNTSSFFWTVSVALMVYW-FHLVSWGVPLGITVAAVAL----LLWML-LTGKVWEILAYIILPVLYVLVFIPVIFIVLRIWSTIRFILFLVILH-GIGN-TFQGGANCIMFVLCTRAI
tr|A0A6P7ZWE1|A0A6P7ZWE1_9AMPH VIVLL----SCAL----SFVGSSLIIFTHVLLLLYLSVADLLSATSYFYGVLVLQGALSTFSNTSSFFWTVAVALLLCW-IHLISWGLPLGITVAAVSL----ILWML-LTGKVWEMLAYIILPIFYLLVIIPLIFIFLRIWSTVRFFLVLVILH-GVGN-TFQGGANCILFVLCTEVI
tr|A0A6P8PU42|A0A6P8PU42_GEOSA VTVLL----SCAL----SFVGSSLIICTHVLLLLYLSVADLLSAVSYFYGVLVLQGALSTFSNTSSFFWTVAVALLLCW-IHLISWGVPLGITVAAVAL----LLWML-LTGKVWEMLAYIILPIFYLLLIIPLIFILLRIWSTVRFFLVLVILH-GVGN-TFQGGANCILFVLCTDVI
tr|F7D2G3|F7D2G3_ORNAN         AVVLL----SCVL----SFLGSGLLVATHALLLLYLSLADLLSAASYFYGVFRLQGALSTFANTSSFFWTVAVAFLLYC-FHVVSWGLPLGITVAAVSL----VLWML-LTGKLWEILAYILLPLLYILILIPIIFICLRIWSTVRFILVLVVLH-GIGN-TFQGGANCIMFVLFTRVI
tr|A0A286XRX7|A0A286XRX7_CAVPO VAVLL----SCTL----SALGCGLLVATHALLLLFLSLADLLSAASYFYGALVLQGALSSFANTSSFFWTMAIALLFWV-FHVVSWGVPLGITVAAVAL----LLWLL-LTGKLWELLAYVTLPVLYLLVLVPLVFIGLRVWSTVRFVLALVLLH-GVGN-TFQGGANCILFVLATRAV
tr|A0A8C2V544|A0A8C2V544_CHILA -AVLL----SCVL----SALGCGLLVATHALLLLFLSLADLLSAASYFYGALVLQGALSSFANTSSFFWTVAIALLFWA-FHAVSWGLPLGITVAAVSL----LLWLL-LTGKLWEMLAYVTLPVLYLLVLIPLIFICLRVWSTVRFVLVLVLLH-GIGN-TFQGGANCIMFVLGTRAI
tr|A0A8C0MI61|A0A8C0MI61_CANLF AVVLL----SCAL----SALGSGLLVATHALLLLFLSLADLLSAASYFYGVLVLQGALSTFANTSSFFWTVAIALLLWA-FHVVSWGVPLGITVAAVAL----ILWML-LTGKLWEMLAYVTLPVLYLLILIPLIFICLRVWSTVRFVLVLVVLH-GLDP-PLPKPPSPTKWVHPSTPA
tr|A0A8I3Q1G7|A0A8I3Q1G7_CANLF AVVLL----SCAL----SALGSGLLVATHALLLLFLSLADLLSAASYFYGVLVLQGALSTFANTSSFFWTVAIALLLWA-FHVVSWGLPLGITVAAVSL----ILWML-LTGKLWEMLAYVTLPVLYLLILIPLIFICLRVWSTVRFVLVLVVLH-GLDP-PLPKPPSPTKWVHPSTPA
tr|G3T4P0|G3T4P0_LOXAF         VVVLL----SCAL----SALGSGLLVATHALLLLFLSLADLLSAASYFYGVLVLHGALSTFANTSSFFWTVAIALLVLX-XXFLSWGVPLGITVAAVAL----ALWML-LTGKLWELLAYVTLPVIYILVLIPLIFICLRVWSTVRFILVLVVLH-GIGN-TFQGGANCIMFVFCTRTV
```

```
tr|A0A8B6ZYS4|A0A8B6ZYS4_ORYAF AVVLL----SCAL----SALGSGLLVATHALLLLFLSLADLLSAVSYFYGVLVLQGALSTFANTSSFFWTVAIALLLWT-FHVVSWGLPLGITVAAVSL----ALWML-LTGKLWEILAYITLPVLYILVLIPLIFICLRVWSTVRFVLVLVVLH-GIGN-TFQGGANCIMFVLCTHTI
tr|A0A9B0TXE0|A0A9B0TXE0_CHRAS AVVLL----SCVL----SALGSGLLVATHALLLLFLSLADLLSAASYFYGVLVLQGALSTFANTSSFFWTVAIALLLWA-FHIISWGVPLGITVAAVAL----ALWML-LTGKLWEILAYITLPVIYILILIPLIFICLRVWSTVRFILVLVVLH-GIGN-TFQGGANCIMFVFCTQTV
tr|A0A287AEG7|A0A287AEG7_PIG   IVVLL----SCVL----SALGSGLLVATHALLLLFLSLADLLSATSYFYGVLVLQGALSTFANTSSFFWTVAIALLFWA-FHIVSWGLPLGITVAAVSL----VLWML-LTGKLWEMLAYIMLPLLYLLVLIPLIFICLRVWSTVRFVLVLVVLH-GVGN-TFQGGANCIMFALCTQAV
tr|A0A8C3VZL6|A0A8C3VZL6_9CETA IVVLL----SCVL----SALGSGLLVATHALLLLFLSLADLLSATSYFYGVLVLQGALSTFANTSSFFWTVAIALLFWA-FHIVSWGVPLGITVAAVAL----VLWML-LTGKLWEMLAYVTLPLLYLLVLIPLIFICLRVWSTVRFVLVLVVLH-GIGN-TFQGGANCIMFALCTRAV
tr|A0A8B8U9S9|A0A8B8U9S9_CAMFR AVVLL----SCAL----SALGSGLLVATHALLLFFLSLADLLSAASYFYGVLVLQGAVSTFASTSSFFWTVAIALLLWV-FHIVSWGLPLGITVAAVSL----VLWML-LTGKLWELLAYVTLPVLYLLVLIPLIFIFLRVWSTVRFILVLVVLH-GIGN-TFQGGANCITFALCTRAV
tr|A0A9W3ETC7|A0A9W3ETC7_CAMBA AVVLL----SCAL----SALGSGLLVATHALLLFFLSLADLLSAASYFYGVLVLQGAVSTFASTSSFFWTVAIALLLWV-FHIVSWGVPLGITVAAVAL----VLWML-LTGKLWELLAYVTLPVLYLLVLIPLIFIFLRVWSTVRFILVLVVLH-GIGN-TFQGGANCITFALCTRAV
tr|A0A250XYS6|A0A250XYS6_CASCN AVVLL----SCVL----SALGSGLLVVTHALLLLFLSLADLLSAASYFYGVLVLQGALSTFANTSSFFWTVAIALLLWA-FHVVSWGLPLGITVAAVSL----VLWML-LTGKLWEMLAYVLLPLLYLLVLIPLIFICLRVWSTVRFVLVLVVLH-GIGN-TFQGGANCIMFVLCTRAV
tr|A0A2Y9Q9T3|A0A2Y9Q9T3_DELLE AVVLL----SCAL----SALGSGLLVATHALLLLFLSLADLLSAASYFYGVLVLQGALSTFANTSSFFWTVAIALLLWA-FHVVSWGVPLGITVAAVAL----VLWML-LTGKLWEMLAYVTLPVIYLLALIPLIFVCLRIWSTVRFVLVLVVLH--VGDREHLSGGRQLHHVRPLHPH
tr|A0A1S3AFM1|A0A1S3AFM1_ERIEU AVVLL----SCAL----SALGSSLLVATHALLLLFLSLADLLSAASYFYGVLVLQGALSTFANTSSFFWTVAIALLFWA-FHIVSWGLPLGITVAAVSL----LLWML-LTGKLWELLAYITLPLLYLLIFIPLIFICLRVWSTVRFVLVLVVLH-GIGN-TFQGGANCIMFVFCTQAV
tr|A0A8C5KG65|A0A8C5KG65_JACJA AVVLL----SCAL----SALGSGLLVATHALLLLFLSLADLLSAASYFYGVLVLQGALSTFANTSSFFWTVAIALLLWA-FHVVSWGVPLGITVAAVAL----VLWML-LTGKLWEMLAYILLPLLYLLVLIPLIFICLRVWSTVRFALVLVILH-GIGN-TFQGGANCIMFVLCTHAI
tr|A0A8C6QFL7|A0A8C6QFL7_NANGA AVVLL----SCAL----SALGSGLLVATHALLLLFLSLADLLSASSYFYGVLVLQGALSTFANTSSFFWTVAIALLLWV-FHVVSWGLPLGITVAAVSL----VLWML-LTGKLWEMLAYILLPLLYLLVLIPLIFICLRVWSTVRFVLVLVVLH-GIGN-TFQGGANCIMFVLCTRVV
sp|Q5FVG1|GP157_RAT            AVVLL----SCVL----SALGSGLLVATHALLLLFLSLADLLSAASYFYGVLVLQGALSTFANTSSFFWTVAIALLVWA-FHLISWGVPLGITVAAVAL----VLWML-LTGKLWEMLAYILLPLLYLLILIPFIFICLRVWSTVRFVLVLVVLH-GIGN-TFQGGANCIMFVLCTRAV
sp|Q8C206|GP157_MOUSE          AVVLL----SCAL----SALGSGLLVATHALLLLFLSLADLLSAASYFYGVLVLQGALSTFANTSSFFWTVAIALLIWA-FHLISWGLPLGITVAAVSL----VLWML-LTGKLWEMLAYILLPLLYLLVLIPLIFICLRVWSTVRFVLVLVVLH-GIGN-TFQGGANCIMFVLCTRAV
tr|Q148S2|Q148S2_MOUSE         AVVLL----SCAL----SALGSGLLVATHALLLLFLSLADLLSAASYFYGVLVLQGALSTFANTSSFFWTVAIALLIWA-FHLISWGVPLGITVAAVAL----VLWML-LTGKLWEMLAYILLPLLYLLVLIPLIFICLRVWSTVRFVLVLVVLH-GIGN-TFQGGANCIMFVLCTRAV
tr|A0A6P5PLA1|A0A6P5PLA1_MUSCR AVVLL----SCAL----SALGSGLLVATHALLLLFLSLADLLSAASYFYGVLVLQGALSTFANTSSFFWTVAIALLIWA-FHLISWGLPLGITVAAVSL----VLWML-LTGKLWEMLAYILLPLLYLLVLIPLIFICLRVWSTVRFVLVLVVLH-GIGN-TFQGGANCIMFVLCTRAV
tr|A0A6I9L1J8|A0A6I9L1J8_PERMB AVVLL----SCVL----SALGSGLLVATHALLLLFLSLTDLLSAASYFYGVLVLQGALSTFANTSSFFWTVAIALLLWA-FHLVSWGVPLGITVAAVAL----VLWML-LTGKLWEMLAYVLLPLLYLLILIPLIFICLRVWSTVRFALVLVVLH-GIGN-TFQGGANCIMFVLCTRAV
tr|A0A1U7QRH5|A0A1U7QRH5_MESAU AVVLL----SCVL----SALGSGLLVATHALLLLFLSLADLLSAASYFYGVLVLQGALSTFANTSSFFWTVAIALLLWA-FHLISWGLPLGITVAAVSL----VMWML-LTGKLWEMLAYVLLPLLYLLILIPLIFICLRVWSTVRFVLVLVVLH-GIGN-TFQGGANCIMFVLCTRAV
tr|A0A8C2M3J7|A0A8C2M3J7_CRIGR AVVLL----SCVL----SALGSGLLVATHALLLLFLSLADLLSAASYFYGVLVLQGALSTFANTSSFFWTVAIALLLWA-FHLISWGVPLGITVAAVAL----VMWML-LTGKLWEMLAYVLLPLLYLLILIPLIFICLRVWSTVRFVLVLVALH-GIGN-TFQGGANCIMFALCTRAV
tr|A0A8B8XUW0|A0A8B8XUW0_BALMU AVVLL----SCAL----SALGSGLLVATHGLLLLFLSLADLLSAASYFYGVLVLQGALSTFANTSSFFWTVAIALLLCA-FHVVSWGLPLGITVAAVSL----VLWML-LTGKVWEMLAYVTLPVLYLLVLIPIIFICLRVWSTVRFVLVLVVLH-GIGN-TFQGGANCIMFVLCTRTV
tr|A0A340X0F8|A0A340X0F8_LIPVE AVVLL----SCAL----SALGSGLLVATHALLLLFLSLADLLSAASYFYGVLVLQGALSTFANTSSFFWTVAIALLLWA-FHVVSWGVPLGITVAAVAL----VLWML-LTGKVWEMLAYVTLPVIYLLALIPLIFICLRVWSTVRFVLVLVVLH-GIGN-TFQGGANCITFVLCTRTV
tr|A0A8C6B6V7|A0A8C6B6V7_MONMO AVVLL----SCAL----SALGSGLLVATHALLLLFLSLADLLSAASYFYGVLVLQGALSTFANTSSFFWTVAIALLLWA-FHVVSWGLPLGITVAAVSL----VLWML-LTGKLWEMLAYVTLPVIYLLALIPLIFVCLRIWSTVRFVLVLVVLH-GIGN-TFQGGANCITFVLCTRTV
tr|A0A341BV22|A0A341BV22_NEOAA AVVLL----SCAL----SALGSGLLVATHALLLLFLSLADLLSAASYFYGVLVLQGALSTFANTSSFFWTVAIALLLWA-FHVVSWGVPLGITVAAVAL----VLWML-LTGKLWEMLAYVTLPVIYLLALIPLIFVCLRIWSTVRFVLVLVVLH-GIGN-TFQGGANCITFVLCTRTV
tr|A0A8C9BRT0|A0A8C9BRT0_PHOSS AVVLL----SCAL----SALGSGLLVATHALLLLFLSLADLLSAASYFYGVLVLQGALSTFANTSSFFWTVAIALLLWA-FHVVSWGLPLGITVAAVSL----VLWML-LTGKLWEMLAYVTLPVIYLLALIPLIFVCLRIWSTVRFVLVLVVLH-GIGN-TFQGGANCITFILCTRTV
tr|A0A2Y9Q9T0|A0A2Y9Q9T0_DELLE AVVLL----SCAL----SALGSGLLVATHALLLLFLSLADLLSAASYFYGVLVLQGALSTFANTSSFFWTVAIALLLWA-FHVVSWGVPLGITVAAVAL----VLWML-LTGKLWEMLAYVTLPVIYLLALIPLIFVCLRIWSTVRFVLVLVVLH-GIGN-TFQGGANCITFVLCTRTV
tr|A0A8D2AWY5|A0A8D2AWY5_SCIVU AVVLL----SCAL----SALGSGLLVATHALLLLFLSLADLLSAASYFYGVLVLQGALSTFANTSSFFWTVAIALLLWA-FHAVSWGLPLGITVAAVSL----VLWML-LTGKLWEMLAYIMLPLLYLLVLIPLIFICLRVWSTVRFVLVLVVLH-GIGN-TFQGGANCIMFVLCTPVV
tr|A0A8D2KC75|A0A8D2KC75_UROPR AVVLL----SCAL----SALGSGLLVATHALLLLFLSLADLLSAASYFYGVLVLQGAVSTFANTSSFFWTVAIALLLWA-FHAVSWGVPLGITVAAVAL----LLWML-LTGKLWEMLAYILLPLLYLLVLIPLIFICLRVWSTVRFVLVLVVLH-GIGN-TFQGGANCIMFVLCTRAV
tr|I3NH70|I3NH70_ICTTR         AVVLL----SCAL----SALGSGLLVATHALLLLFLSLADLLSAASYFYGVLVLQGAVSTFANTSSFFWTVAIALLLWA-FHAVSWGLPLGITVAAVSL----LLWML-LTGKLWEMLAYILLPLLYLLVLIPLIFICLRVWSTVRFVLVLVVLH-GIGN-TFQGGANCIMFVLCTRAV
tr|A0A6J0WE95|A0A6J0WE95_ODOVR AVVLL----SCVL----SAVGSGLLITTHALLLLFLSLADLLSAASYFYGVLVLQGALSTFANTSSFFWTVAIALLLWT-FHVVSWGVPLGITVAAVAL----VLWML-LTGKLWELLAYATLPVLYLLVLIPLIFICLRVWSTVRFVLLLVVLH-GIGN-TFQGGANCIMFVFCTRAV
tr|A0A452FBX1|A0A452FBX1_CAPHI AVVLL----SCVL----SALGSGLLMATHALLLLFLSLADLLSAASYFYGVLVLQGALSTFANTSSFFWTVAIALLLWT-FHVVSWGLPLGITVAAVSL----VLWML-LTGKLWELLAYVTLPVLYLLVLIPLIFICLRVWSTVRFVLVLVALH-GIGN-TFQGGANCVMFVFCTRAV
tr|A0A452FBX7|A0A452FBX7_CAPHI AVVLL----SCVL----SALGSGLLMATHALLLLFLSLADLLSAASYFYGVLVLQGALSTFANTSSFFWTVAIALLLWT-FHVVSWGVPLGITVAAVAL----VLWML-LTGKLWELLAYVTLPVLYLLVLIPLIFICLRVWSTVRFVLVLVALH-GIGN-TFQGGANCVMFVFCTRAV
tr|A0A8C6DLL2|A0A8C6DLL2_MOSMO AVVLL----SCVL----SALGSGLLMATHALLLLFLSLADLLSAASYFYGVLVLQGALSTFANTSSFFWTVAIALLLWA-FHVVSWGLPLGITVAAVSL----VLWML-LTGKLWELLAYVTLPVLYLLVLIPLIFICLRVWSTVRFVLVLVVLH-GIGN-TFQGGANCIMFVFCTRAV
tr|A0A4W2HZG6|A0A4W2HZG6_BOBOX AVVLL----SCVL----SALGSGLLMATHALLLLFLSLADLLSAASYFYGVLVLQGALSTFANTSSFFWTVAIALLLWA-FHVISWGVPLGITVAAVAL----VLWML-LTGKLWELLAYVTLPVLYLLVLIPLIFICLRVWSTVRFVLVLVVLH-GIGN-TFQGGANCIMFVFCTRVV
tr|A0A6P5D3W5|A0A6P5D3W5_BOSIN AVVLL----SCVL----SALGSGLLMATHALLLLFLSLADLLSAASYFYGVLVLQGALSTFANTSSFFWTVAIALLLWA-FHVISWGLPLGITVAAVSL----VLWML-LTGKLWELLAYVTLPVLYLLVLIPLIFICLRVWSTVRFVLVLVVLH-GIGN-TFQGGANCIMFVFCTRVV
tr|F1MUY8|F1MUY8_BOVIN         AVVLL----SCVL----SALGSGLLMATHALLLLFLSLADLLSAASYFYGVLVLQGALSTFANTSSFFWTVAIALLLWA-FHVISWGVPLGITVAAVAL----VLWML-LTGKLWELLAYVTLPVLYLLVLIPLIFICLRVWSTVRFVLVLVVLH-GIGN-TFQGGANCIMFVFCTRVV
tr|A0A8B9X996|A0A8B9X996_BOSMU AVVLL----SCVL----SALGSGLLMATHALLLLFLSLADLLSAASYFYGVLVLQGALSTFANTSSFFWTVAIALLLWA-FHVISWGLPLGITVAAVSL----VLWML-LTGKLWELLAYVTLPVLYLLVLIPLIFICLRVWSTVRFILVLVVLH-GIGN-TFQGGANCIMFVFCTRVV
tr|A0A6P6BZ89|A0A6P6BZ89_PTEVA AVVLL----SCVL----SALGSGLLVATHALLLLFLSLADLLSAASYFYGVLVLQGALSTFANTSSFFWTVAIALLLWA-FHVVSWGVPLGITVAAVAL----VLWML-LTGKLWELLAYVALPALYLLVLIPLIFICLRVWSTVRFVLVLVILH-GIGN-TFQGGANCIMFVLCTRVV
tr|A0A6J2LN53|A0A6J2LN53_9CHIR AVVLL----SCVL----SALGSGLLVATHALLLLFLSLADLLSAVSYFYGVLVLQGALSTFANTSSFFWTVAIALLLWA-FHVVSWGLPLGITVAAVSL----VLWML-LTGKLWEMLAYVTLPVLYLLVLIPVIFICLRVWSTVRFVLVLVVLH-GIGN-TFQGGANCIMFVLCTRAV
tr|A0A671F9D5|A0A671F9D5_RHIFE AVVLL----SCVL----SALGSGLLVATHALLLLFLSLADFLSAVSYFYGVLVLQGAVSTFANTSSFFWTVAIALLLWA-FHIVSWGVPLGITVAAVAL----VLWML-LTGKLWELLAYVTLPVLYLLVVIPLIFICLRVWSTVRFVLVLVVLH-GIGN-TFQGGANCIMFVFCTRVV
tr|H0WS24|H0WS24_OTOGA         AVVLL----SCAL----SALGSGLLVATHALLLLFLSLADLLSAASYFYGVLVLQGALSTFANTSSFFWTVAIALLLWV-FHVVSWGLPLGITVAAVSL----VLWML-LTGKLWEMLAYITLPLLYLLVLIPLIFICLRVWSTVRFVLVLVVLH-GIGN-TFQGGANCIMFVLCTRAV
tr|A0A8B7IAR3|A0A8B7IAR3_MICMU AVVLL----SCAL----SALGSGLLVATHALLLLFLSLADLLSAASYFYGVLVLQGALSTFANTSSFFWTVAIALLLWG-FHVVSWGVPLGITVAAVAL----ILWML-LAGKLWEMLAYVTLPLLYLLVLIPLIFICLRVWSTVRFVLVLVILH-GIGN-TFQGGANCIMFVLCTRAV
tr|A0A2K6GR83|A0A2K6GR83_PROCO AVVLL----SCAL----SALGSGLLVATHALLLLFLSLADLLSAASYFYGVLVLQGALSTFANTSSFFWTVAIALLFWI-FHVVSWGLPLGITVAAVSL----VLWML-LTGKLWEMLAYVTLPLLYLLVLIPLIFICLRVWSTVRFVLVLVVLH-GIGN-TFQGGANCIMFVLCTRAV
tr|A0A8C9AIR7|A0A8C9AIR7_PROSS AVVLL----SCAL----SALGSGLLVATHALLLLFLSLADLLSAASYFYGVLVLQGALSTFANTSSFFWTVAIALLLWV-FHVVSWGVPLGITVAAVAL----VLWML-LTGKLWEMLAYVTLPLLYLLVLIPLIFICLRVWSTVRFVLVLVVLH-GIGN-TFQGGANCIMFVLCTRAV
tr|A0A8I3WPH6|A0A8I3WPH6_CALJA AVVLL----SCAL----SAFGSGLLVATHALLLLFLSLADLLSAASYFYGVLVLQGALSTFANTSSFFWTVAVALLLWI-FHVVSWGLPLGITVAAVSL----LLWML-LTGKLWEMLAYVLLPLLYVLVLIPLIFICLRVWSTVRFVLVLVVLH-GIGN-TFQGGANCIMFVLCTRAV
tr|A0A2K5DZ75|A0A2K5DZ75_AOTNA AVVLL----SCAL----SAFGSGLLVATHALLLLFLSLADLLSAASYFYGVLVLQGALSTFANTSSFFWTVAIALLLWV-FHVVSWGVPLGITVAAVAL----LLWML-LTGKLWEMLAYVLLPLLYLLVLIPLIFICLRVWSTVRFVLVLVVLH-GIGN-TFQGGANCIMFVLCTRAV
tr|A0A6J3JP52|A0A6J3JP52_SAPAP AVVLL----SCAL----SAFGSGLLVATHALLLLFLSLADLLSAASYFYGVLVLQGALSTFANTSSFFWTVAIALLLWV-FHIVSWGLPLGITVAAVSL----LLWML-LTGKLWEMLAYVLLPLLYLLVLIPLIFICLRVWSTVRFVLVLVVLH-GIGN-TFQGGANCIMFVLCTRAV
tr|A0A2K6KZA9|A0A2K6KZA9_RHIBE AVVLL----SCAL----SALGSGLLVATHALLLLFLSLADLLSAASYFYGVLVLQGALSTFANTSSFFWTVAIAFLFWA-FHVVSWGVPLGITVAAVAL----VLWML-LTGKLWEMLAYILLPLLYLLVLIPLIFICLRVWSTVRFVLVLVVLH-GIGN-TFQGGANCIMFVLCTRAV
tr|A0A2K6RR93|A0A2K6RR93_RHIRO AVVLL----SCAL----SALGSGLLVATHALLLLFLSLADLLSAASYFYGVLVLQGALSTFANTSSFFWTVAIAFLFWA-FHVVSWGLPLGITVAAVSL----VLWML-LTGKLWEMLAYILLPLLYLLVLIPLIFICLRVWSTVRFVLVLVVLH-GIGN-TFQGGANCIMFVLCTRAV
tr|A0A5F7ZT31|A0A5F7ZT31_MACMU TVVLL----SCAL----SALGSGLLVATHALLLLFLSLADLLSAASYFYGVLVLQGALSTFANTSSFFWTVAIAFLLWA-FHVVSWGVPLGITVAAVAL----VLWML-LTGKLWEMLAYILLPLLYLLVLIPLIFICLRVWSTVRFVLVLVVLH-GIGN-TFQGGANCIMFVLCTRAV
tr|A0A2K6BG10|A0A2K6BG10_MACNE TVVLL----SCAL----SALGSGLLVATHALLLLFLSLADLLSAASYFYGVLVLQGALSTFANTSSFFWTVAIAFLLWA-FHVVSWGLPLGITVAAVSL----VLWML-LTGKLWEMLAYILLPLLYLLVLIPLIFICLRVWSTVRFVLVLVVLH-GIGN-TFQGGANCIMFVLCTRAV
tr|A0A2K5TW36|A0A2K5TW36_MACFA TVVLL----SCAL----SALGSGLLVATHALLLLFLSLADLLSAASYFYGVLVLQGALSTFANTSSFFWTVAIAFLLWA-FHVVSWGVPLGITVAAVAL----VLWML-LTGKLWEMLAYILLPLLYLLVLIPLIFICLRVWSTVRFVLVLVVLH-GIGN-TFQGGANCIMFVLCTRAV
tr|A0A8C9GBZ9|A0A8C9GBZ9_9PRIM AVVLL----SCAL----SALGSGLLVATHALLLLFLSLADLLSAASYFYGVLVLQGALSTFANTSSFFWTVAIAFLFWA-FHVVSWGLPLGITVAAVSL----VLWML-LTGKLWEMLAYILLPLLYLLVLIPLIFICLRVWSTVRFVLVLVVLH-GIGN-TFQGGANCIMFVLCTRAV
tr|A0A096N790|A0A096N790_PAPAN AVVLL----SCAL----SALGSGLLVATHALLLLFLSLADLLSAASYFYGVLVLQGALSTFANTSSFFWTVAIAFLLWA-FHVVSWGVPLGITVAAVAL----VLWML-LTGKLWEMLAYILLPLLYLLVLIPLIFICLRVWSTVRFVLVLVVLH-GIGN-TFQGGANCIMFVLCTRAV
tr|A0A2K5NDS0|A0A2K5NDS0_CERAT AVVLL----SCAL----SALGSGLLVATHALLLLFLSLADLLSAASYFYGVLVLQGALSTFANTSSFFWTVAIAFLLWA-FHVVSWGLPLGITVAAVSL----VLWML-LTGKLWEMLAYILLPLLYLLVLIPLIFICLRVWSTVRFVLVLVVLH-GIGN-TFQGGANCIMFVLCTRAV
tr|A0A0D9S8Q2|A0A0D9S8Q2_CHLSB AVVLL----SCAL----SALGSGLLVATHALLLLFLSLADLLSAASYFYGVLVLQGALSTFANTSSFFWTVAIAFLLWA-FHVVSWGVPLGITVAAVAL----VLWML-LTGKLWEMLAYILLPLLYLLVLIPLIFICLRVWSTVRFVLVLVVLH-GIGN-TFQGGANCIMFVLCTRAV
tr|G1RDI8|G1RDI8_NOMLE         AVVLL----SCAL----SALGSGLLVATHALLLLFLSLADLLSAASYFYGVLVLQGALSTFANTSSFFWTVAIALLLWA-FHVVSWGLPLGITVAAVSL----VLWML-LTGKLWELLAYVLLPLLYLLVLIPLIFICLRVWSTVRFVLVLVVLH-GIGN-TFQGGANCIMFVLCTRAV
tr|H2N977|H2N977_PONAB         AVVLL----SCAL----SALGSGLLVATHALLLLFLSLADLLSAASYFYGVLVLQGALSTFANTSSFFWTVAIALLLWA-FHVVSWGVPLGITVAAVAL----VLWML-LTGKLWEMLAYVLLPLLYLLVLIPLIFICLRVWSTVRFVLVLVVLH-GIGN-TFQGGANCIMFVLCTHTV
sp|Q5UAW9|GP157_HUMAN          AVVLL----SCAL----SALGSGLLVATHALLLLFLSLADLLSAASYFYGVLVLQGALSTFANTSSFFWTVAIALLLWA-FHVVSWGLPLGITVAAVSL----VLWML-LTGKLWEMLAYVLLPLLYLLVLIPLIFIGLRVWSTVRFVLVLVVLH-GIGN-TFQGGANCIMFVLCTRAV
```

```
tr|A8KA23|A8KA23_HUMAN         AVVLL----SCAL----SALGSGLLVATHALLLLFLSLADLLSAASYFYGVLVLQGALSTFANTSSFFWTVAIALLLWA-FHVVSWGVPLGITVAAVAL----VLWML-LTGKLWEMLAYVLLPLLYLLVLIPLIFIGLRVWSTVRFVLVLVVLH-GIGN-TFQGGANCIMFVLCTRAV
tr|A0A2R9AW71|A0A2R9AW71_PANPA AVVLL----SCVL----SALGSGLLVATHALLLLFLSLADLLSAASYFYGVLVLQGALSTFANTSSFFWTVAIALLLWA-FHVVSWGLPLGITVAAVSL----VLWML-LTGKLWEMLAYVLLPLLYLLVLIPLIFIGLRVWSTVRFVLVLVVLH-GIGN-TFQGGANCIMFVLCTRAI
tr|A0A6D2X9H8|A0A6D2X9H8_PANTR AVVLL----SCVL----SALGSGLLVATHALLLLFLSLADLLSAASYFYGVLVLQGALSTFANTSSFFWTVAIALLLWA-FHVVSWGVPLGITVAAVAL----VLWML-LTGKLWEMLAYVLLPLLYLLVLIPLIFIGLRVWSTVRFVLVLVVLH-GIGN-TFQGGANCIMFVLCTRAV
tr|H2R9I7|H2R9I7_PANTR         AVVLL----SCVL----SALGSGLLVATHALLLLFLSLADLLSAASYFYGVLVLQGALSTFANTSSFFWTVAIALLLWA-FHVVSWGLPLGITVAAVSL----VLWML-LTGKLWEMLAYVLLPLLYLLVLIPLIFIGLRVWSTVRFVLVLVVLH-GIGN-TFQGGANCIMFVLCTRAV
tr|A0A8C4N2Q3|A0A8C4N2Q3_EQUAS AVVLL----SCAL----SALGSGLLVATHALLLLFLSLADLLSAASYFYGVLVLQGALSTFANTSSFFWTVAIALLLWA-FHIVSWGVPLGITVAAVAL----ILWML-LTGKLWEMLAYVTLPLLYLLVLIPLIFIFLRVWSTVRFVLVLVVLH-GIGN-TFQGGANCIMFVLCTRAV
tr|A0A9L0K136|A0A9L0K136_EQUAS AVVLL----SCAL----SALGSGLLVATHALLLLFLSLADLLSAASYFYGVLVLQGALSTFANTSSFFWTVAIALLLWA-FHIVSWGLPLGITVAAVSL----ILWML-LTGKLWEMLAYVTLPLLYLLVLIPLIFIFLRVWSTVRFVLVLVVLH-GIGN-TFQGGANCIMFVLCTRAV
tr|A0A6G1AYR8|A0A6G1AYR8_CROCR ----------CAL----SALGSGLLVATHALLLLFLSLADLLSAASYFYGVLVLQGALSTFANTSSFFWTVAIALLLWA-FHVVSWGVPLGITVAAVAL----VLWML-LTGKLWEMLAYVTLPVLYLLVLIPLIFICLRVWSTVRFVLVLVVLH-GIGN-TFQGGANCIMFVLCTRAV
tr|A0A8U0N6W8|A0A8U0N6W8_MUSPF AVVLL----SCVL----SALGSGLLVATHALLLLFLSLADLLSAASYFYGVLVLQGALSTFANTSSFFWTVAIALLLWA-FHVVSWGLPLGITVAAVSL----LLWML-LTGKLWEMLAYVTLPVLYFLIFIPLIFICLRVWSTVRFVLVLVVLH-GIGN-TFQGGANCIMFVLCTQAV
tr|M3Y4L6|M3Y4L6_MUSPF         AVVLL----SCVL----SALGSGLLVATHALLLLFLSLADLLSAASYFYGVLVLQGALSTFANTSSFFWTVAIALLLWA-FHVVSWGVPLGITVAAVAL----LLWML-LTGKLWEMLAYVTLPVLYFLIFIPLIFICLRVWSTVRFVLVLVVLH-GIGN-TFQGGANCIMFVLCTQAV
tr|G1LBD9|G1LBD9_AILME         AVVLL----SCAL----SALGSGLLVATHALLLLFLSLADLLSAASYFYGVLVLQGALSTFANTSSFFWTVAIALLLWA-FHVVSWGLPLGITVAAVSL----VLWML-LTGKLWEMLAYVTLPVLYFLILIPLIFICLRVWSTVRFVLVLVVLH-GIGN-TFQGGANCIMFALCTQAV
tr|A0A384CFC9|A0A384CFC9_URSMA AVVLL----SCAL----SALGSGLLVATHALLLLFLSLADLLSAASYFYGVLVLQGALSTFANTSSFFWTVAIALLLWA-FHVVSWGVPLGITVAAVAL----VLWML-LTGKLWEMLAYVSLPVLYFLILIPLIFICLRVWSTVRFVLVLVVLH-GIGN-TFQGGANCIMFVLCTQAV
tr|A0A452SG29|A0A452SG29_URSAM AVVLL----SCAL----SALGSGLLVATHALLLLFLSLADLLSAASYFYGVLVLQGALSTFANTSSFFWTVAIALLLWA-FHVVSWGLPLGITVAAVSL----VLWML-LTGKLWEMLAYVSLPVLYFLILIPLIFICLRVWSTVRFVLVLVVLH-GIGN-TFQGGANCIMFVLCTQAV
tr|A0A8C8X5H6|A0A8C8X5H6_PANLE AVVLL----SCAL----SALGSGLLVATHALLLLFLSLADLLSAASYFYGVLVLQGALSTFANTSSFFWTVAIALLLWA-FHVVSWGVPLGITVAAVAL----VLWML-LTGKLWEMLAYVTLPVLYLLVLIPLIFICLRVWSTVRFVLVLVVLH-GIGN-TFQGGANCIMFVLCTHAV
tr|A0A9V1G7L6|A0A9V1G7L6_PANPR AVVLL----SCAL----SALGSGLLVATHALLLLFLSLADLLSAASYFYGVLVLQGALSTFANTSSFFWTVAIALLLWA-FHVVSWGLPLGITVAAVSL----VLWML-LTGKLWEMLAYVTLPVLYLLVLIPLIFICLRVWSTVRFVLVLVVLH-GIGN-TFQGGANCIMFVLCTHAV
tr|A0A5F5XE30|A0A5F5XE30_FELCA AVVLL----SCAL----SALGSGLLVATHALLLLFLSLADLLSAASYFYGVLVLQGALSTFANTSSFFWTVAIALLLWA-FHVVSWGVPLGITVAAVAL----VLWML-LTGKLWEMLAYVTLPVLYLLVLIPLIFICLRVWSTVRFVLVLVVLH-GIGN-TFQGGANCIMFVLCTHAV
tr|A0A667HHU7|A0A667HHU7_LYNCA AVVLL----SCAL----SALGSGLLVATHALLLLFLSLADLLSAASYFYGVLVLQGALSTFANTSSFFWTVAIALLLWA-FHVVSWGLPLGITVAAVSL----VLWML-LTGKLWEMLAYVTLPVLYLLVLIPLIFICLRVWSTVRFVLVLVVLH-GIGN-TFQGGANCIMFVLCTHAV
tr|A0A3Q7SY12|A0A3Q7SY12_VULVU AVVLL----SCAL----SALGSGLLVATHALLLLFLSLADLLSAASYFYGVLVLQGALSTFANTSSFFWTVAIALLLWA-FHVVSWGVPLGITVAAVAL----ILWML-LTGKLWEMLAYVMLPVLYLLILIPLIFICLRVWSTVRFVLVLVVLH-GIGN-TFQGGANCIMFVLCTQAV
tr|A0A8C0SI91|A0A8C0SI91_CANLF AVVLL----SCAL----SALGSGLLVATHALLLLFLSLADLLSAASYFYGVLVLQGALSTFANTSSFFWTVAIALLLWA-FHVVSWGLPLGITVAAVSL----ILWML-LTGKLWEMLAYVTLPVLYLLILIPLIFICLRVWSTVRFVLVLVVLH-GIGN-TFQGGANCIMFVLCTQAV
tr|A0A8I3Q553|A0A8I3Q553_CANLF AVVLL----SCAL----SALGSGLLVATHALLLLFLSLADLLSAASYFYGVLVLQGALSTFANTSSFFWTVAIALLLWA-FHVVSWGVPLGITVAAVAL----ILWML-LTGKLWEMLAYVTLPVLYLLILIPLIFICLRVWSTVRFVLVLVVLH-GIGN-TFQGGANCIMFVLCTQAV
tr|A0A8C0JZV9|A0A8C0JZV9_CANLU AVVLL----SCAL----SALGSGLLVATHALLLLFLSLADLLSA-----------GALSTFANTSSFFWTVAIALLLWA-FHVVSWGLPLGITVAAVSL----ILWML-LTGKLWEMLAYVTLPVLYLLILIPLIFICLRVWSTVRFVLVLVVLH-GIGN-TFQGGANCIMFVLCTQAV
tr|A0A2U3VR77|A0A2U3VR77_ODORO AVVLL----SCAL----SALGSGLLVATHALLLLFLSLADLLSAASYFYGVLVLQGALSTFANTSSFFWTVAIALLLWA-FHVVSWGVPLGITVAAVAL----ILWML-LTGKLWEMLAYVTLPVIYCLMLIPLTFICLRVWSTVRFVLVLVVLH-GIGN-TFQGGANCIMFVLCTQAV
tr|A0A3Q7MWU7|A0A3Q7MWU7_CALUR AVVLL----SCAL----SALGSGLLVATHALLLLFLSLADLLSAASYFYGVLVLQGALSTFANTSSFFWTVAIALLLWA-FHVVSWGLPLGITVAAVSL----ILWML-LTGKLWEMLAYVMLPVIYCLMLIPLTFICLRVWSTVRFVLVLVVLH-GIGN-TFQGGANCIMFVLCTQAV
tr|A0A6J2FAF4|A0A6J2FAF4_ZALCA AVVLL----SCAL----SALGSGLLVATHALLLLFLSLADLLSAASYFYGVLVLQGALSTFANTSSFFWTVAIALLLWA-FHVVSWGVPLGITVAAVAL----ILWML-LTGKLWEMLAYVALPVIYCLMLIPLTFICLRVWSTVRFVLVLVVLH-GIGN-TFQGGANCIMFVLCTQAV
tr|A0A2U3XTE7|A0A2U3XTE7_LEPWE AVVLL----SCAL----SALGSGLLVATHALLLLFLSLADLLSAASYFYGVLVLQGALSTFANTSSFFWTVAIALLLWA-FHVVSWGLPLGITVAAVSL----VLWML-LTGKLWEMLAYVTLPVLYCLILIPLIFICLRVWSTVRFVLVLVVLH-GIGN-TFQGGANCIMFVLCTQAV
tr|A0A2Y9GKK8|A0A2Y9GKK8_NEOSC AVVLL----SCAL----SALGSGLLVATHALLLLFLSLADLLSAASYFYGVLVLQGALSTFANTSSFFWTVAIALLLWA-FHVVSWGVPLGITVAAVAL----VLWML-LTGKLWEMLAYVTLPVLYCLILIPLIFICLRVWSTVRFVLVLVVLH-GIGN-TFQGGANCIMFVLCTQAV
tr|A0A8C6XWN3|A0A8C6XWN3_NAJNA GLVLL----SCAL----SVLGCCLLLGTQARLLSCLSGADLLSAASYAYGVLVAQGALSTFANTSSFFWTVAIALLLCC-FHLVSWGLPLGITVAAVSL----LLWML-LAGKLWEILAYVTLPFFYILTLIPIIFIFLRIWSTIRFVLLLVVLH-GVGN-TFQGAANCILFVFCTRVV
tr|A0A670YGI2|A0A670YGI2_PSETE GMVLL----SCAL----SVLGCCLLLGTQARLLSCLSGADLLSAASYAYGVLVAQGALSTFANTSSFFWTVAIALLLCC-FHLVSWGVPLGITVAAVAL----LLWML-LAGKLWEILAYVTLPFFYILTLIPIVFIFLRIWSTIRFVLLLVVLH-GVGN-TFQGAANCILFVFCTRVV
tr|A0A6J1VNE1|A0A6J1VNE1_9SAUR GMVLL----SCAL----SVLGCCLLLGTQACLLSCLSGADLLSAASYAYGVLVAQGALSTFANTSSFFWTVAIALLLCC-FHLVSWGLPLGITVAAVSL----LLWML-LAGKLWEILAYVTLPFFYILTLIPIVFIFLRIWSTIRFVLLLVVLH-GVGN-TFQGAANCILFVFCTRVV
tr|A0A8C5RK72|A0A8C5RK72_LATLA GMVLL----SCAL----SVLGCCLLLGTQARLLSCLSGADLLSAASYAYGVLVAQGALSTFANTSSFFWTVAIALLLCC-FHLVSWGVPLGITVAAVAL----LLWML-LAGKLWEILAYVTLPFFYILTLIPIVFIFLRIWSTIRFVLLLVVLH-GVGN-TFQGAANCILFVFCTRVV
tr|A0A670YMU6|A0A670YMU6_PSETE GMVLL----SCAL----SVLGCCLLLGTQARLLSCLSGADLLSAASYAYGVLVAQGALSTFANTSSFFWTVAIALLLCC-FHLVSWGLPLGITVAAVSL----LLWML-LAGKLWEILAYVTLPFFYILTLIPIVFIFLRIWSTIRFVLLLVVLH-GVGN-TFQGAANCILFVFCTRVV
tr|A0A7N4NQ56|A0A7N4NQ56_SARHA IVVLL----SCVL----SFLGSSLLIGTHALLLLFLSLADLLSAASYFYGVLVVQGALSTFANTSSFFWTVAIAILLYC-FHIISWGVPLGITVAAVAL----LLWML-LAGKVWELLAYLVLPILYILILIPLIFICLRVWSTVRFILVLVVLH-GIGN-TFQGGANCIMFVLCTQAI
tr|A0A4X2MCH8|A0A4X2MCH8_VOMUR IVVLL----SCVL----SFLGSSLLIGTHALLLLFLSLADLLSAASYFYGVLVVQGALSTFANTSSFFWTVAIAILLYC-FHLISWGLPLGITVAAVSL----LLWML-LAGKVWEMLAYLVLPVLYILILIPLIFICLRVWSTVRFILVLVVLH-GIGN-TFQGGANCIMFVLCTQAV
tr|A0A6P5JG96|A0A6P5JG96_PHACI IVVLL----SCVL----SFLGSSLLIGTHALLLLFLSLADLLSAASYFYGVLVVQGALSTFANTSSFFWTVAIAILLYC-FHIISWGVPLGITVAAVAL----LLWML-LAGKVWEILAYLVLPVLYVLILIPLIFICLRVWSTVRFILVLVVLH-GIGN-TFQGGANCIMFVLCTQAV
tr|A0A6J0SRV4|A0A6J0SRV4_9SAUR SIRQD----QSAL----SGLGCVLLAGTHARLLLWLSLADLLSAAAYFYGALVAQGALSTFANTSSFFWTVAIALLLGL-FHAVSWGLPLGITVAAVSL----VLWML-LAGKMWEILAYVTLPIFYILVLIPVIFIFLRIWSTVRFVLILVVLH-GIGN-TFQGGANCIMFVFCTRVV
tr|A0A8D0BT79|A0A8D0BT79_SALMN IVVLV----SCTL----SFLGAGLLVGTHALLLLYLSLADLFSAASYFYGVLVAQGAISTFANTSSFFWTMAIALLLCF-FHVVSWGVPLGITVAAVAL----VLWML-LAGKVWEILAYITLPIFYILALIPVIFISLRIWSTVRFILLLVVLH-GIGN-TFQGGANCILFVLCTRVV
tr|A0A670JAZ3|A0A670JAZ3_PODMU VTVLV----SCAL----SFLGSSLLVGTHALLLLYLSLADLLSAASYFYGALVAQGAVSTFSNTSSFFWTMAIALLLCF-FHVVSWGLPLGITVAAVSL----ILWML-LAGKLWEILAYVTLPVFYILILIPVIFIFLRIWSTIRFVLLLVVLH-GIGN-TFQGGANCIMFVLCTRVV
tr|A0A7K6BKJ8|A0A7K6BKJ8_UPUEP GVVLV----SCVF----SFVGSSLLVGTHALLLLYLSLADLLSSISYFYGVLVLQGALSTFANTSSFFWTMAIAILLCC-FHGVSWGVPLGITVAAVAL----LFWML-LTGKVWEILAYVTLPVLYILILIPVIFIILRIWSTVRFVLLLVVLH-GIGN-TFQGGANCIMFVLCTRVV
tr|A0A7L1NE03|A0A7L1NE03_RHICY GVVLV----SCVL----SFVGSSLLVGTHALLLLYLSLADLLSAISYFYGVLVLQGALSTFANTSSFFWTMAIALLLCC-FHGVSWGLPLGITVAAVSL----LFWML-LTGKVWEILAYVTLPVLYILILIPVIFIILRIWSTVRFVLLLVVLH-GIGN-TFQGGANCIMFVLCTRVV
tr|A0A7K7WPR8|A0A7K7WPR8_9AVES VVVLL----SCAL----SFVGSSILVGTHALLLLYLSLADLLSALSYFYGVLVLQGALSTFSNTSSFFWTMAIALLLCC-FHVVSWGVPLGITVAAVAL----LLWML-LTGKIWEILAYVTLPVLYILILIPVIFIILRIWSTVRFILVLVVLH-GIGN-TFQGGANCIMFVLCTRVV
tr|A0A851T4U9|A0A851T4U9_9AVES VVVLL----SCAL----SFVGSSILVGTHALLLLYLSLADLLSALSYFYGVLVLQGALSTFSNTSSFFWTMAIALLLCC-FHVVSWGLPLGITVAAVSL----LLWML-LTGKIWEISAYVTLPVLYILVLIPVIFIILRIWSTVRFILVLVVLH-GIGN-TFQGGANCIMFVLCTRVV
tr|A0A7K7VIS8|A0A7K7VIS8_EUDEL VVVLL----SCAL----SSAGSGLLVGTHALLLLYLSLADLLSALSYFYGVLVLQGALSTFANTSSFFWTMAIALLLCC-FHVVSWGVPLGITVAAVAL----VLWML-LTGKVWEILAYVTLPVLYVLILIPVIFIILRIWSTVRFILVLVVLH-GIGN-TFQGGANCIMFVLCTRAV
tr|A0A8C6YW20|A0A8C6YW20_NOTPE VAVLL----SCAL----SFVGSGLLVGTHALLLLYLSLADLLSALSYFYGVLVLQGALSTFSNTSSFFWTMAIALLLCC-FHVVSWGLPLGITVAAVSL----VLWML-LTGKVWEILAYVTLPVLYLLILIPVIFIILRIWSTVRFILVLVVLH-GIGN-TFQGGANCIMFVLCTRVV
tr|A0A7K6ZU36|A0A7K6ZU36_9AVES VAVLL----SCAL----SFVGSGLLVGTHALLLLYLSLADLLSALSYFYGVLVLQGALSTFSNTSSFFWTMAIALLLCC-FHVVSWGVPLGITVAAVAL----VLWML-LTGKVWEILAYVTLPVLYLLILIPVIFIVLRIWSTVRFILVLVVLH-GIGN-TFQGGANCIMFVLCTRVV
tr|A0A7K7AW82|A0A7K7AW82_9AVES VAVLL----SCAL----SFVGSGLLVGTHALLLLYLSLADLLSALSYFYGVLVLQGALSTFSNTSSFFWTMAIALLLCC-FHVVSWGLPLGITVAAVSL----VLWML-LTGKVWEILAYVTLPVLYLLILIPVIFIVLRIWSTVRFILVLVVLH-GIGN-TFQGGANCIMFVLCTRVV
tr|A0A7K4KF62|A0A7K4KF62_9AVES --VLL----SCAL----SCVGSGLLVGTHALLLLYLSLADLLSALSYFYGVLVLQGALSTFSNTSSFFWTMAIALLLCC-FHAVSWGVPLGITVAAVAL----VLWML-LTGKVWEILAYVTLPVLYILILIPVIFIVLRIWSTVRFILVLVVLH-GIGN-TFQGGANCIMFVLCTRAV
tr|A0A7K4LZG7|A0A7K4LZG7_9AVES AVVLL----SCAL----SFVGSGLLVGTHALLLLYLSLADLLSALSYFYGVLVLQGALSTFSNTSSFFWTMAIALLLCC-FHAVSWGLPLGITVAAVSL----VLWML-LTGKVWEILAYVTLPVLYILILIPVIFIVLRIWSTVRFILVLVVLH-GIGN-TFQGGANCIMFVLCTRAV
tr|A0A7K8ZYG6|A0A7K8ZYG6_9PASS AVVLL----SCLL----SCVGSALLLCSHALLLLYLSLADLLSALSYFYGVLVLQGALSTFANTSSFFWTMAIALLLCC-FHAVSWGVPLGITVAAVAL----VLWML-LTGKIWEILAYVTLPVLYILVLIPVIFIFLRIWSTVRFVLLLVVLH-GIGN-TFQGGANCILFVLCTQVV
tr|A0A7L0K1T5|A0A7L0K1T5_CHATO AVVLG----SCLL----SCLGSGLLLCTHALLLLYLSLADLLSALSYFYGVLVLQGALSTFSNTSSFFWTMAIALLLCC-FHVVSWGLPLGITVAAVSL----VLWLL-LTGKIWEILAYVTLPVLYILILIPVIFIILRIWSTVRFILILVVLH-GIGN-TFQGGANCIMFVLCTRVV
tr|A0A7K9V276|A0A7K9V276_ANSSE AVVLA----SCLL----SCLGSGLLLCTHALLLLYLSVADLLSALSYFYGVLVLQGALSTFSNTSSFFWTTAVALLLCC-FHAVSWGVPLGITVAAVAL----VLWML-LTGKVWEILAYVTLPVLYILILIPVIFIFLRIWSTVRFILVLVVLH-GIGN-TFQGGANCIMFVFCTRVV
tr|A0A8B9BSW1|A0A8B9BSW1_9AVES AVVLA----SCLL----SCLGSSLLLCTHALLLLYLSLADLLSALSYFYGVLVLQGALSTFANTSSFFWTMAIALLLCC-FHAVSWGLPLGITVAAVSL----LLWML-LTGKVWEILAYVTLPVLYILILIPVIFIILRIWSTVRFILVLVVLH-GIGN-TFQGGANCIMFVLCTRVV
tr|A0A8B9TUD3|A0A8B9TUD3_ANAPL AVVLV----SCLL----SCLGSCLLLCTHALLLLYLSLADLLSALSYFYGVLVLQGALSTFSNTSSFFWTMAIALLLCC-FHAVSWGVPLGITVAAVAL----VLWML-LTGKVWEILAYVTLPVLYILILIPVIFIILRIWSTVRFILVLVVLH-GIGN-TFQGGANCIMFVLCTRVV
tr|A0A6J3E586|A0A6J3E586_AYTFU AVVLV----SCLL----SCLGSSLLLCTHALLLLYLSLADLLSALSYFYGVLVLQGALSTFSNTSSFFWTMAIALLLCC-FHAVSWGLPLGITVAAVSL----VLWML-LTGKVWEILAYVTLPVLYILILIPVIFIILRIWSTVRFILVLVVLH-GIGN-TFQGGANCIMFVLCTRVV
tr|A0A7K7KXL4|A0A7K7KXL4_9AVES AVVLV----SCLL----SCLGSSLLLCTHALLLLYLSLADLLSALSYFYGVLVLQGALSTFSNTSSFFWTMAIALLLCC-FHAVSWGVPLGITVAAVAL----VLWML-LTGKVWEILAYVTLPVLYILILIPVIFIILRIWSTVRFILVLVVLH-GIGN-TFQGGANCIMFVLCTRVV
tr|A0A7K9YAC5|A0A7K9YAC5_9GALL AAVLT----SCVL----SSLGSGLLVGTHALLLLYLSLADLLSALSYFYGVLVLQGALSTFSNTSSFFWTMAVALLLWC-FHVVSWGLPLGITVAAVSL----VLWML-LTGKVWEILAYVTLPVLYILILIPVIFIILRIWSTVRFVLVLVVLH-GIGN-TFQGGANCIMFVLCTRVV
tr|A0A8C2TJ68|A0A8C2TJ68_COTJA AAVLT----SCVL----SSLGSGLLVGTHALLLLYLSLADLLSALSYFYGVLVLQGALSTFSNTSSFFWTMAVALLLCC-FHVVSWGVPLGITVAAVAL----VLWML-LTGKVWEILAYVTLPVLYILILIPVIFIILRIWSTVRFILVLVVLH-GIGN-TFQGGANCIMFVLCTRVV
tr|A0A7L0WFJ9|A0A7L0WFJ9_ALELA AVVLA----SCLL----SSLGSGLLVGTHALLLLYLSLADLLSAVSYFYGVLVLQGAVSTFSNTSSFFWTMAIALLLCC-FHVVSWGLPLGITVAAVSL----VLWML-LTGKIWEILAYVTLPVLYILILIPVIFIILRIWSTVRFILVLVVLH-GIGN-TFQGGANCIMFVLCTRVV
tr|A0A851N7Y6|A0A851N7Y6_9GALL AAVLT----SCVL----SSLGSGLLVGTHALLLLYLSLADLLSALSYFYGVLVLQGALSTFSNTSSFFWTMAIALLLCF-FHVVSWGVPLGITVAAVAL----VLWML-LTGKVWEILAYVTLPVLYILILIPVIFIILRIWSTVRFILILVVLH-GIGN-TFQGGANCIMFVLCTRVV
```

```
tr|A0A7K5HQ34|A0A7K5HQ34_CROSL VAVLV----SCAL----SALGSALLGCTHALLLLYLSLADLLSALSYFYGVLVLQGALSTFSNTSSFFWTMAIALLLCC-FHAVSWGLPLGITVAAVSL----LLWML-LTGKLWEMLAKKNLPVLYILILIPVIFIILRIWSTVRFVLVLVVLH-GIGN-TFQGGANCIMFVLCTRAV
tr|A0A7K7F0X9|A0A7K7F0X9_CHIMN AAVLA----SCAL----SALGSALLVCAHALLLLYLSLADLLSALSYFYGALVLQGALSTFANTSSFFWTMAVALLLCG-FHVVSWGVPLGITVAAVAL----VLWML-LTGKAWEILAYVTLPVLYMLVLIPVIFIILRIWSTVRFILVLVVLH-GIGN-TFQGGANCIMFVLCTRVV
tr|A0A7L3DK44|A0A7L3DK44_PLUSO AAVLA----SCAL----SALGSALLVCAHALLLLYLSLADLLSALSYFYGALVLQGALSTFANTSSFFWTMAVALLFCG-FHVVSWGVPLGITVAAVSL----VLWML-LTGKVWEILAYVTLPVLYMLVLIPVIFIILRIWSTVRFILVLVVLH-GIGN-TFQGGANCIMFVLCTRVV
tr|A0A7L3L147|A0A7L3L147_9CHAR GVVLG----SCLL----SCLGSALLLGTHARLLLYLSLADLLSALSYFYGVLVLQGALSTFSNTSSFFWTMAIALLLCC-FHIVSWGVPLGITVAAVAL----VLWML-LAGKVWEILAYVTLPVFYILILIPVIFIILRIWSTVRFILVLVVLH-GIGN-TFQGGANCIMFVLCTRVV
tr|A0A7K9DR99|A0A7K9DR99_9AVES VVVLL----SCVL----SFLGSSLLVCTHALLLLYLSLADLLSALSYFYGVLVLQGALSTFSNTSSFFWTMAIAVLLCC-FHAVSWGLPLGITVAAVSL----VLWML-ITGKVWEILAYVILPVLYILILIPVIFIILRIWSTVRFILVLVVLH-GIGN-TFQGGANCIMFVLCTRAV
tr|A0A7K8YTD8|A0A7K8YTD8_9PASS AVVLL----SCVL----SCVGSALLLCSHALLLLYLSLADLLSALSYFYGVLELQGALSTFANTSSFFWTMAIALLLCC-FHAVSWGVPLGITVAAVAL----VLWML-LTGKVWEILAYVTLPVLYVLILIPVIFIFLRIWSTVRFILVLVVLH-GIGN-TFQGGANCIMFVLCTRVV
tr|A0A973YHI2|A0A973YHI2_9PASS AVVLL----SCVL----SCVGSALLLCSHALLLLYLSLADLLSALSYFYGVLELQGALSTFANTSSFFWTMAIALLLCC-FHAVSWGVPLGITVAAVSL----VLWML-LTGKVWEILAYVTLPVLYLLILIPVIFIFLRIWSTVRFILVLVVLH-GIGN-TFQGGANCIMFVLCTRVV
tr|A0A7L0NB58|A0A7L0NB58_9PASS AVVLL----SCVL----SCVGSALLLCSHALLLLYLSLADLLSALSYFYGVLVLQGALSTFANTSSFFWTMAVALLLCC-FHAVSWGVPLGITVAAVAL----VLWML-LTGKLWEILAYVTLPVLYILVLIPVIFIFLRIWSTVRFILVLVVLH-GIGN-TFQGGANCIMFVLCTRVV
tr|A0A7K5AR09|A0A7K5AR09_9FURN AVVLL----SCVL----SCVGSALLLCSHALLLLYLSLADLLSALSYFYGVLVLQGALSTFANTSSFFWTVAIALLLCC-FHAVSWGLPLGITVAAVSL----VLWML-LTGKVWEILAYVTLPVLYILILIPVIFIFLRIWSTVRFILALVVLH-GIGN-TFQGGANCIMFVLCTRVV
tr|A0A851F6U2|A0A851F6U2_PITSO AVVLL----SCVL----SCVGSGLLLCSHALLLLYLSLADLLSALSYFYGVLVLQGALSTFSNTSSFFWTMAIALLLCC-FHVVSWGVPLGITVAAVAL----VLWML-LTGKVWEILAYVTLPVLYILVLIPVIFIALRIWSTVRFILVLVVLH-GIGN-TFQGGANCIMFVLCTRVV
tr|A0A7L2RF30|A0A7L2RF30_9PASS VVVLV----SCVL----SCVGCGLLLCSHALLLLYLSLADLLSALSYFYGVLVLQGALSTFSNTSSFFWTMAIALLLCC-FHVVSWGVPLGITVAAVAL----VLWML-LTGKVWEILAYVTLPVLYILVLIPVIFIILRIWSTVRFILVLVVLH-GIGN-TFQGGANCIMFVLCTRVV
tr|A0A7L1DH58|A0A7L1DH58_9PASS VVVLV----SCVL----SCVGSGLLLCSHALLLLYLSLADLLSALSYFYGVLVLQGALSTFSNTSSFFWTMAIALLLCC-FHVVSWGVPLGITVAAVAL----VLWML-LTGKIWEILAYVTLPVLYILVLIPVIFIFLRIWSTVRFILVLVVLH-GIGN-TFQGGANCIMFVLCTRVV
tr|A0A851BXJ2|A0A851BXJ2_CALVR VVVLV----SCVL----SCVGSGLLLCSHALLLLYLSLADLLSALSYFYGVLVLQGALSTFSNTSSFFWTMAIALLLCC-FHVVSWGVPLGITVAAVSL----VLWML-LTGKVWEILAYVTLPVLYILVLIPVIFIILRIWSTVRFILVLVVLH-GIGN-TFQGGANCIMFVLCTRVV
tr|A0A7K7SH10|A0A7K7SH10_9TYRA VVVLV----SCVL----SCVGSGLLLCSHALLLLYLSLADLLSALSYFYGVLVLQGALSTFSNTSSFFWTMAIALLLCC-FHVVSWGVPLGITVAAVAL----VLWML-LTGKVWEILAYVTLPVLYILVLIPVIFIFLRIWSTVRFILVLVVLH-GIGN-TFQGGANCIMFVLCTRVV
tr|A0A7K8QIA0|A0A7K8QIA0_9PASS VVVLV----SCVL----SCVGSGLLLCSHALLLLYLSLADLLSALSYFYGVLVLQGALSTFSNTSSFFWTMAIALLLCC-FHIVSWGVPLGITVAAVSL----VLWML-LTGKVWEILAYVTLPVLYILVLIPVIFIFLRIWSTVRFILVLVVLH-GIGN-TFQGGANCIMFVLCTRVV
tr|A0A7L3NYG6|A0A7L3NYG6_9DEND AVVLL----SCVL----SCVGSALLLCSHALLLLYLSLADLLSALSYFYGVLVLQGALSTFANTSSFFWTMAIALLLCC-FHAVSWGVPLGITVAAVAL----VLWML-LTGKAWEILAYVTLPVLYILILIPVIFIFLRIWSTVRFILVLVVLH-GIGN-TFQGGANCIMFVLCTRVV
tr|A0A851MIB8|A0A851MIB8_9DEND AVVLL----SCVL----SCVGSALLLCSHALLLLYLSLADLLSALSYFYGVLVLQGALSTFANTSSFFWTMAIALLLCC-FHAVSWGVPLGITVAAVSL----VLWML-LTGKAWEILAYVTLPVLYILILIPVIFIFLRIWSTVRFILVLVVLH-GIGN-TFQGGANCIMFVLCTRVV
tr|A0A7K8WIE9|A0A7K8WIE9_9FURN AVVLL----SCVL----SCVGSALLLCSHALLLLYLSLADLLSALSYFYGVLVLQGALSTFANTSSFFWTMAIALLLCC-FHAVSWGVPLGITVAAVSL----VLWML-LTGKVWEILAYVTLPVLYILILIPVIFIFLRIWSTVRFILVLVVLH-GIGN-TFQGGANCIMFVLCTRVV
tr|A0A7K4W4T1|A0A7K4W4T1_9TYRA AVVLV----SCVL----SCVGSSLLLCSHALLLLYLSLADLLSALSYFYGVLVLQGALSTFANTSSFFWTMAIALLLCC-FHAVSWGLPLGITVAAVSL----VLWML-LTGKVWEILAYVTLPVLYILILIPVIFIFLRIWSTVRFILVLVVLH-GIGN-TFQGGANCIMFVVCTRVV
tr|A0A7K4QDC8|A0A7K4QDC8_9TYRA AVVLV----SCVL----SCVGSSLLLCSHALLLLYLSLADLLSALSYFYGVLVLQGALSTFANTSSFFWTMAIALLLCC-FHAVSWGVPLGITVAAVAL----VLWML-LTGKVWEILAYVTLPVLYILILIPVIFIFLRIWSTVRFILVLVVLH-GIGN-TFQGGANCIMFVLCTRVV
tr|A0A6J0I3E0|A0A6J0I3E0_9PASS AVVLV----SCVL----SCVGSSLLLCSQALLLLYLSLADLLSALSYFYGVLVLQGALSTFANTSSFFWTMAIALLLCC-FHAVSWGLPLGITVAAVSL----VLWML-LTGKVWEILAYVTLPVLYILILIPVIFIFLRIWSTVRFILALVVLH-GIGN-TFQGGANCIMFVLCTRVV
tr|A0A6J2H9V8|A0A6J2H9V8_9PASS AVVLV----SCVL----SCVGSSLLLCSQALLLLYLSLADLLSALSYFYGVLVLQGALSTFANTSSFFWTMAIALLLCC-FHAVSWGVPLGITVAAVAL----VLWML-LTGKVWEILAYVTLPVLYILILIPVIFIFLRIWSTVRFVLALVVLH-GIGN-TFQGGANCIMFVLCTRVV
tr|A0A7K5ZP87|A0A7K5ZP87_ONYCO AVVLV----SCVL----SCVGSSILLCSHALLLLYLSLADLLSALSYFYGVLVLQGALSTFANTSSFFWTMAIALLLCC-FHAVSWGLPLGITVAAVSL----VLWML-LTGKVWEILAYVTLPVLYLLILIPVIFIFLRIWSTVRFILVLVVLH-GIGN-TFQGGANCIMFVLCTRVV
tr|A0A7K5KBJ1|A0A7K5KBJ1_9TYRA AVVLV----SCVL----SCVGSSLLLCSHALLLLYLSLADLLSALSYFYGVLVLQGALSTFANTSSFFWTMAIALLLCC-FHAVSWGLPLGITVAAVSL----VLWML-LTGKVWEILAYVTLPVLYILVLIPVIFIFLRIWSTVRFILVLVVLH-GIGN-TFQGGANCIMFVLCTRVV
tr|A0A7L0XBW0|A0A7L0XBW0_TYRSA AVVLV----SCVL----SCVGSSLLLCSHALLLLYLSLADLLSALSYFYGVLVLQGALSTFANTSSFFWTMAIALLLCC-FHAVSWGLPLGITVAAVSL----VLWML-LTGKVWEILAYVTLPVLYILVLIPVIFIFLRIWSTVRFILVLVVLH-GIGN-TFQGGANCIMFVLCTRVV
tr|A0A7K5CYF0|A0A7K5CYF0_9TYRA AVVLV----SCVL----SCVGSSLLLCSHALLLLYLSLADLLSALSYFYGVLVLQGALSTFANTSSFFWTMAIALLLCC-FHGVSWGVPLGITVAAVAL----VLWML-LTGKAWEILAYITLPVLYILILIPVIFIFLRIWSTVRFILILVVLH-GIGN-TFQGGANCIMFVLCTRVV
tr|A0A7K5U8Y7|A0A7K5U8Y7_CEPOR AVVLV----SCVL----SCVGSSLLLCSHALLLLYLSLADLLSALSYFYGVLVLQGALSTFANTSSFFWTMAIALLLCC-FHGVSWGLPLGITVAAVSL----VLWML-LTGKVWEILAYVTLPVLYILILIPVIFIFLRIWSTVRFILVLVVLH-GIGN-TFQGGANCIMFVLCTRVV
tr|A0A7L0ZPK6|A0A7L0ZPK6_9PASS AVVLV----SCVL----SCVGSSLLLCSHALLLLYLSLADLLSALSYFYGVLVLQGALSTFANTSSFFWTMAIALLLCC-FHAVSWGLPLGITVAAVSL----VLWML-LTGKVWEILAYVTLPVLYILILIPVIFIFLRIWSTVRFILVLVVLH-GIGN-TFQGGANCIMFVLCTRVV
tr|A0A7L0IYK5|A0A7L0IYK5_PIPCL AVVLV----SCVL----SCVGSSLLLCSHALLLLYLSLADLLSALSYFYGVLVLQGALSTFANTSSFFWTMAIALLLCC-FHAVSWGLPLGITVAAVSL----VLWML-LTGKVWEILAYVTLPVLYILILIPVIFIFLRIWSTVRFILVLVVLH-GIGN-TFQGGANCIMFVLCTRVV
tr|A0A8B7JZ32|A0A8B7JZ32_9AVES IVVLL----SCVL----SFLGSSLLVCTHALLLLYLSLADLLSALSYFYGVLVLQGALSTFSNTSSFFWTMAIALLLCC-FHVVSWGVPLGITVAAVAL----VLWML-LTGKVWEILAYVTLPVLYILILIPVIFIILRIWSTVRFILVLVVLH-GIGN-TFQGGANCIMFVLCTRVV
tr|A0A7K8NIL2|A0A7K8NIL2_CASCA IAVLV----SCVL----SFLGSSLLVGTHALLLLYLSLADLLSALSYFYGVLVLQGALSTFSNTSSFFWTMAIALLLCC-FHVVSWGLPLGITVAAVSL----VLWML-LTGKVWEILAYVTLPVLYILILIPVIFIILRIWSTVRFILVLVVLH-GIGN-TFQGGANCIMFVLCTRVV
tr|A0A7K9AXP4|A0A7K9AXP4_DRONO IVVLA----SCVL----SFLGSSLLVCTHALLLLYLSLADLLSALSYFYGVLVLQGALSTFSNTSSFFWTMAIALLLCC-FHVVSWGVPLGITVAAVAL----VLWML-LTGKVWEILAYVTLPVLYILILIPVIFIILRIWSTVRFILVLVVLH-GIGN-TFQGGANCIMFVLCTRVV
tr|A0A8C4PD72|A0A8C4PD72_DRONO IVVLA----SCVL----SFLGSSLLVCTHALLLLYLSLADLLSALSYFYGVLVLQGALSTFSNTSSFFWTMAIALLLCC-FHVVSWGVPLGITVAAVSL----VLWML-LTGKVWEILAYVTLPVLYILILIPVIFIILRIWSTVRFILVLVVLH-GIGN-TFQGGANCIMFVLCTRVV
tr|A0A7L3NK92|A0A7L3NK92_9AVES --VLI----SCVL----SFLGSSLLVCTHALLLLYLSLADLLSALSYFYGVLVLQGALSTFSNTSSFFWTMAIAVLLCC-FHVVSWGVPLGITVAAVAL----VMWML-LTGKVWEMLAYLILPVLYILVLIPVIFIILRIWSTVRFILVLVVLH-GIGN-TFQGGANCVMFVLCTRVV
tr|A0A7K5YD28|A0A7K5YD28_9AVES VVVLV----SCVF----SFVGSSLLVCTHALLLLYLSLADLLSALSYFYGVLVLQGALSTFANTSSFFWTMAIALLLCC-FHVVSWGLPLGITVAAVSL----VLWML-LTGKVWEILAYVTLPVLYLLILIPVIFIILRIWSTVRFILVLVVLH-GIGN-TFQGGANCIMFVLCTRVV
tr|A0A672UUG2|A0A672UUG2_STRHB VVVLV----SCGL----SFLGSSLLVCTHALLLLYLSLADLLSALSYFYGVLVLQGALSTFSNTSSFFWTMAIALLVCC-FHVMSWGVPLGITVAAVAL----VLWML-LTGKVWEILAYVTLPVLYLLILIPVIFIILRIWSTVRFILVLVVLH-GIGN-TFQGGANCIMFVLCTRVI
tr|A0A7L0MKY9|A0A7L0MKY9_9PSIT VVVLL----SCAL----SFLGSSLLVCTHALLLLYLSLADLLSALSYFYGVLVLQGALSTFSNTSSFFWTMAIAVLLCC-FHVMSWGLPLGITVAAVSL----LLWML-LTGKVWEILAYVTLPVLYLLILIPVIFIILRIWSTVRFILVLVVLH-GIGN-TFQGGANCIMFVLCTRVV
tr|A0A7K5FQI6|A0A7K5FQI6_PROAR VVVLV----SCVL----SFLGSSLLVCTHALLLLYLSLADLLSALSYFYGVLVLQGALSTFSNTSSFFWTMAVAVLLCC-FHVMSWGVPLGITVAAVAL----VLWML-LTGKVWEILAYVTLPVLYLLILIPVIFIILRIWSTVRFILVLVVLH-GIGN-TFQGGANCIMFVLCTRVV
tr|A0A851X6T1|A0A851X6T1_EOLRO VVVLV----SCVL----SFLGSSLLVCTHALLLLYLSLADLLSALSYFYGVLVLQGALSTFSNTSSFFWTMAIAVLLCC-FHVMSWGVPLGITVAAVSL----VLWML-LTGKVWEILAYVTLPVLYLLILIPVIFIILRIWSTVRFILVLVVLH-GIGN-TFQGGANCIMFVLCTRVV
tr|A0A7L4NCZ8|A0A7L4NCZ8_9AVES VVVLV----SCVL----SFLGSSLLVCTHALLLLYLSLADLLSSLSYFYGVLVLQGALSTFSNTSSFFWTMAIAFLLCC-FHVVSWGVPLGITVAAVAL----VLWML-LTGKVWEILAYVTLPVLYILILIPVIFIILRIWSTVRFILLVVLH-GIGN-TFQGGANCIMFVLCTRAV
tr|A0A7L4DUI6|A0A7L4DUI6_9AVES AVVLV----SCVL----SCLGSSLLVCSHALLLCLSLADLLSALSYFYGVLVLQGALSTFSNTSSFFWTMAIALLLCC-FHAVSWGLPLGITVAAVSL----VLWML-LTGKLWEILAYVTLPVLYILILIPVIFIILRIWSTVRFILVLVVLH-GIGN-TFQGGANCIMFVLCTRAV
tr|A0A7K9C157|A0A7K9C157_9PICI AVVLV----SCVL----SSLGSSLLVCTHALLLLYLSLADLLSALSYFYGVLVLQGALSTFSNTSSFFWTMAIALLLCC-FHVVSWGVPLGITVAAVAL----LLWML-LTGKVWEMLAYVTLPVLYILILIPVIFIVLRIWSTVRFILVLVVLH-GIGN-TFQGGANCIMFVLCTRLV
tr|A0A7L1GGQ4|A0A7L1GGQ4_9PICI AVVLV----SCVL----SSLGSSLLVCTHALLLLYLSLADLLSALSYFYGVLVLQGALSTFSNTSSFFWTMAIALLLCC-FHVVSWGLPLGITVAAVSL----LLWML-LTGKVWEILAYVTLPVLYILILIPVIFIILRIWSTVRFILVLVVLH-GIGN-TFQGGANCIMFVLCTRVV
tr|A0A7L2IIN1|A0A7L2IIN1_9PICI AVVLV----SCVL----SSLGSSLLVCTHALLLLYLSLADLLSALAYFYGVLVLQGALSTFSNTSSFFWTMAIALLLCC-FHVVSWGVPLGITVAAVAL----LLWML-LTGKVWEILAYVTLPVLYILILIPVIFIVLRIWSTVRFILVLVVLH-GIGN-TFQGGANCIMFVLCTRLV
tr|A0A852BRM2|A0A852BRM2_9PICI AVVLV----SCVL----SSLGSSLLVCTHALLLLYLSLADLLSALSYFYGVLVLQGALSTFSNTSSFFWTMAIALLLCC-FHVVSWGLPLGITVAAVSL----LLWML-LTGKVWEILAYVTLPVLYILILIPVIFIVLRIWSTVRFILVLVVLH-GIGN-TFQGGANCIMFVLCTRLV
tr|A0A852IQ55|A0A852IQ55_9PICI AVVLV----SCVL----SSLGSSLLVCTHALLLLYLSLADLLSALSYFYGVLVLQGALSTFSNTSSFFWTMAIALLLCC-FHVVSWGVPLGITVAAVAL----LLWML-LTGKVWEILAYVTLPVLYILILIPVIFIVLRIWSTIRFILVLVVLH-GIGN-TFQGGANCIMFVLCTRLV
tr|A0A852LW28|A0A852LW28_9AVES VVVLV----SCVL----SFLGSSLLVCTHALLLLYLSLADLLSALSYFYGVLVLQGALSTFSNTSSFFWTMAIALLLCC-FHAVSWGLPLGITVAAVSL----VLWML-LTGKVWEILAYVTLPVLYILILIPVIFIILRIWSTVRFILVLVVLH-GIGN-TFQGGANCIMFVLCTRAV
tr|A0A7K4ZMV4|A0A7K4ZMV4_9AVES VVVLV----SCVL----SFLGSSLLVCTHALLLLYLSLADLLSALSYFYGVLVLQGALSTFSNTSSFFWTMAIALLLCC-FHAVSWGVPLGITVAAVAL----VLWML-LTGKVWEILAYVTLPVLYILILIPVIFIILRIWSTVRFILVLVVLH-GIGN-TFQGGANCIMFVLCTRAV
tr|A0A850X701|A0A850X701_PIACA VIVLV----SCVL----SFLGSSLLVCTHALLLLYLSLADLLSALSYFYGVLVLQGALSTFSNTSSFFWTMAIALLLCC-FHVVSWGLPLGITVAAVSL----VLWML-LTGKVWEILAYVTLPVLYILILIPVIFIILRIWSTVRFILVLVVLH-GIGN-TFQGGANCIMFVLCTRAV
tr|A0A7L4JV63|A0A7L4JV63_9AVES VIVLV----SCVL----SFLGSSLLVCTHALLLLYLSLADLLSALSYFYGVLVLQGALSTFSNTSSFFWTMAIALLLCC-FHVVSWGVPLGITVAAVAL----VLWML-LTGKVWEILAYVTLPVLYILILIPVIFIILRIWSTVRFILVLVVLH-GIGN-TFQGGANCIMFVLCTRAV
tr|A0A7L2VIM5|A0A7L2VIM5_9AVES VVVLV----SCVL----SFLGSSLLVCTHALLLLYLSLADLLSALSYFYGVLVLQGALSTFSNTSSFFWTMAIALLLCC-FHVVSWGLPLGITVAAVSL----VLWML-LTGKVWEILAYVTLPVLYILILIPVIFIILRIWSTVRFILVLVVLH-GIGN-TFQGGANCIMFVLCTRVV
tr|A0A7K6TQI9|A0A7K6TQI9_9AVES VVVLV----SCVL----SFLGSSLLVCTHALLLLYLSLADLLSALSYFYGVLVLQGALSTFSNTSSFFWTMAIAVLLCS-FHAVSWGVPLGITVAAVAL----VLWML-LTGKVWEMLAYVTLPVLYVLILIPAIFIILRIWSTVRFILVLVVLH-GIGN-TFQGGANCIMFVLCTRVV
tr|A0A852K9Z2|A0A852K9Z2_UROIN VVVLV----SCVL----SFLGSSLLVCTHAMLLLYLSLADLLSALSYFYGVLVLQGALSTFSNTSSFFWTMAIALLLCC-FHVVSWGLPLGITVAAVSL----VLWML-LTGKVWEILAYVTLPVLYILILIPIIFIILRIWSTVRFILVLVVLH-GIGN-TFQGGANCIMFVLCTRVV
tr|A0A7K9HYD0|A0A7K9HYD0_9AVES VVVLV----SCVL----SFLGSSLLVCTHALLLLYLSLADLLSALSYFYGVLVLQGALSTFSNTSSFFWTMAIALLLCC-FHGVSWGVPLGITVAAVAL----LLWML-LTGKVWEILAYVTLPVLYILILIPVIFIILRIWSTVRFILVLVVLH-GIGN-TFQGGANCIMFVLCTRLV
tr|A0A7K5G9K2|A0A7K5G9K2_9AVES VVVLV----SCGL----SFLGSSLLVCTHALLLLYLSLADLLSALSYFYGVLVLQGALSTFSNTSSFFWTMAIALLLCC-FHVVSWGLPLGITVAAVSL----VLWML-LTGKVWEILAYVTLPVLYILILIPIIFIILRIWSTVRFVLVLVVLH-GIGN-TFQGGANCIMFVLCTRVV
tr|A0A7L4GLY7|A0A7L4GLY7_PODST VAVLV----SCAL----SFLGSGLLVCTHALLLLYLSLADLLSALSYFYGVLVLQGALSTFSNTSSFFWTMAIALLLCC-FHAVSWGVPLGITVAAVAL----VLWML-LTGKVWEILAYVTLPILYVLILIPVIFIILRIWSTVRFVLVLVVLH-GIGN-TFQGGANCIMFVLCTRVV
tr|A0A7K9XW40|A0A7K9XW40_9GRUI VVVLV----SCVL----SFLGSSLLVCTHALLLLYLSLADLLSALSYFYGVLVLQGALSTFSNTSSFFWTMAIALLLCG-FHVVSWGLPLGITVAAVSL----VVWML-LTGKVWEILAYVTLPVLYILILIPVIFIILRIWSTVRFILVLVWLH-GIGN-TFQGGANCIMFVLCTRVV
```

```
tr|A0A7L0DUR1|A0A7L0DUR1_TROML VVVLV----SCVL----SFLGSSLLVCTHALLLLYLSLADLLSALSYFYGVLVLQGALSTFSNTSSFFWTMAVALLLCL-FHAVSWGVPLGITVAAVAL----VLWML-LTGKIWEMLAYVILPVLYILILIPVIFIVLRIWSTVRFILVLVVLH-GIGN-TFQGGANCIMFVLCTRVV
tr|A0A7L1IZU0|A0A7L1IZU0_SMUAF AVVLG----SCVL----SLLGSSLLVCTHALLLLYLSLADLLSALSYFYGVLVLQGALSTFSNTSSFFWTMAVALLLCC-FHVVSWGLPLGITVAAVSL----VLWML-LTGKVWEILAYVTLPVLYILILIPVIFIILRIWSTVRFILLVVLH-GIGN-TFQGGANCIMFVLCTRVV
tr|A0A7L4LUZ2|A0A7L4LUZ2_GLAPT AVVLV----SCVL----SFLGSSLLVCTHALLLLYLSLADLLSALSYFYGVLVLQGALSTFANTSSFFWTMAVALLLCC-FHVVSWGVPLGITVAAVAL----VLWML-LTGKVWEILAYVTLPVLYILILIPVIFIVLRIWSTVRFILLLVVLH-GIGN-TFQGGANCIMFVLCTRVV
tr|A0A7K5X3P1|A0A7K5X3P1_9CHAR VVVLV----SCAL----SFLGSSLLVCSHALLLLYLSLADLLSALSYFYGVLVLQGALSTFSNTSSFFWTMAVALLLCC-FHVVSWGLPLGITVAAVSL----VLWML-LTGKVWEILAYVTLPVLYILILIPVIFIVLRIWSTVRFILLLVVLH-GIGN-TFQGGANCIMFVLCTRVV
tr|A0A7L1JE25|A0A7L1JE25_RYNNI GVVLV----SCVL----SFLGSSLLVCTHALLLLYLSLADLLSALSYFYGVLVLQGALSTFSNTSSFFWTMAVALLLCC-FHIVSWGVPLGITVAAVAL----VLWML-LTGKVWEILAYVTLPVLYVLILIPVIFIVLRIWSTVRFILLLVVLH-GIGN-TFQGGANCIMFVLCTRVV
tr|A0A7K5N527|A0A7K5N527_CHRMC GVVLV----SCVL----SFLGSSLLVCTHALLLLYLSLADLLSALSYFYGVLVLQGALSTFSNTSSFFWTMAVALLLCC-FHVVSWGLPLGITVAAVSL----VLWML-LTGKVWEILAYVTLPVLYILILIPVIFIVLRIWSTVRFILLLVVLH-GIGN-TFQGGANCIMFVLCTRVV
tr|A0A7L3T5E0|A0A7L3T5E0_RISTR GVVLV----SCVL----SFLGSSLLVCTHALLLLYLSLADLLSALSYFYGVLVLQGALSTFSNTSSFFWTMAVALLLCC-FHIVSWGVPLGITVAAVAL----VLWML-LTGKVWEILAYVTLPVLYILILIPVIFIVLRIWSTVRFILLLVVLH-GIGN-TFQGGANCIMFVLCTRVV
tr|A0A852GCP3|A0A852GCP3_9CHAR GVVLV----SCVL----SFLGSSLLVCTHALLLLYLSLADLLSALSYFYGVLVLQGALSTFSNTSSFFWTMAVALLLCC-FHVVSWGLPLGITVAAVSL----VLWML-LTGKVWEILAYVTLPVLYILILIPVIFIVLRIWSTVRFILLLVVLH-GIGN-TFQGGANCIMFVLCTRVV
tr|A0A7L3RX08|A0A7L3RX08_CEPGR GVVLV----SCVL----SFLGSSLLVCTHALLLLYLSLADLLSALSYFYGVLVLQGALSTFSNTSSFFWTMAVALLLCC-FHVVSWGVPLGITVAAVAL----VLWML-LTGKVWEILAYVTLPVLYILILIPVIFIVLRIWSTVRFILLLVVLH-GIGN-TFQGGANCIMFVLCTRVV
tr|A0A7L3TQC0|A0A7L3TQC0_URIAL GVVLV----SCVL----SFLGSSLLVCTHALLLLYLSLADLLSALSYFYGVLVLQGALSTFSNTSSFFWTMAVALLLCC-FHVVSWGLPLGITVAAVSL----VLWML-LTGKVWEILAYVTLPVLYILILIPVIFIVLRIWSTVRFILLLVVLH-GIGN-TFQGGANCIMFVLCTRAV
tr|A0A7K6YAQ1|A0A7K6YAQ1_ALCTO GVVLV----SCVL----SFLGSSLLVCTHALLLLYLSLADLLSALSYFYGVLVLQGALSTFSNTSSFFWTMAVALLLCC-FHVVSWGVPLGITVAAVAL----VLWML-LTGKVWEILAYVTLPVLYILILIPVIFIVLRIWSTVRFILLLVVLH-GIGN-TFQGGANCIMFVLCTRVV
tr|A0A7K9FBD4|A0A7K9FBD4_STEPR GVVLV----SCVL----SFLGSSLLVCTHALLLLYLSLADLLSALSYFYGVLVLQGALSTFSNTSSFFWTMAVALLLCC-FHVVSWGLPLGITVAAVSL----VLWML-LTGKVWEILAYVTLPVLYILILIPVIFIVLRIWSTVRFILLLVVLH-GIGN-TFQGGANCIMFVLCTRVV
tr|A0A7L4F5C6|A0A7L4F5C6_9COLU VVVLV----SCVL----SFLGSSLLVCTHALLLLYLSLADLLSALSYFYGVLVLQGALSTFSNTSSFFWTMAIALLLCC-FHAVSWGVPLGITVAAVAL----VLWML-LAGKVWEILAYVTLPVLYILILIPVIFIILRIWSTVRFVLVLVVLH-GIGN-TFQGGANCIMFVLCTRVV
tr|A0A7K6T0D1|A0A7K6T0D1_CALNI VVVLL----SCVL----SFLGSSLLVCTHALLLLYLSLADLLSALSYFYGVLVLQGALSTFSNTSSFFWTMAIALLLCC-FHAVSWGLPLGITVAAVSL----VLWML-LAGKVWEILAYVTLPVLYILILIPIIFIILRIWSTVRFVLVLVVLH-GIGN-TFQGGANCITFVLCTRVV
tr|A0A1V4KNW4|A0A1V4KNW4_PATFA VVVLL----SCVL----SFLGSSLLVCTHALLLLYLSLADLLSALSYFYGVLVLQGALSTFSNTSSFFWTMAIALLLCC-FHAVSWGVPLGITVAAVAL----VLWML-LAGKVWEILAYVTLPVLYILILIPIIFIILRIWSTVRFVLVLVVLH-GIGN-TFQGGANCIMFVLCTRVV
tr|A0A2I0M3E7|A0A2I0M3E7_COLLI VVVLV----SCVL----SFLGSSLLVCTHALLLLYLSLADLLSALSYFYGVLVLQGALSTFSNTSSFFWTMAIALLLCC-FHAVSWGLPLGITVAAVSL----VLWML-LAGKVWEILAYVTLPVLYILILIPIIFIILRIWSTVRFVLVLVVLH-GIGN-TFQGGANCIMFVLCTRVV
tr|A0A7L0TYT9|A0A7L0TYT9_CHOAC VVVLI----SCVL----SFLGSSLLLCTHALLLLYLSLADLLSAISYFYGVLVLQGALSTFSNTSSFFWTMAIALLLCC-FHVVSWGVPLGITVAAVAL----VLWML-LTGKVWEILAYVTLPVLYILILIPIIFIILRIWSTVRFILFLVVLH-GIGN-TFQGGANCIMFVLCTRVV
tr|A0A7L4C7U0|A0A7L4C7U0_9AVES VVVLI----SCVL----SFLGSSLLVCTHALLLLYLSLADLLSAISYFYGVLVLQGALSTFSNTSSFFWTMAIALLLCF-FHVVSWGLPLGITVAAVSL----VLWML-LTGKVWEILAYVTLPVLYILILIPVIFIILRIWSTVRFILFLVVLH-GIGN-TFQGGANCIMFVLCTRVV
tr|A0A7L2AL05|A0A7L2AL05_9GRUI VVVLV----SCVL----SFLGSSLLVCTHALLLLYLSLADLLSALSYFYGVLVLQGALSTFSNTSSFFWTMAIALLLCC-FHVVSWGVPLGITVAAVAL----LLWML-LTGKVWEILAYVTLPVLYILILIPVIFIILRIWSTVRFILVLVVLH-GIGN-TFQGGANCIMFVLCTRVV
tr|A0A851YTY8|A0A851YTY8_9AVES VVVLV----SCVL----SFLGSSLLVCTHALLLLYLSLADLLSALSYFYGVLVLQGALSTFSNTSSFFWTMAIALLLCC-FHIVSWGLPLGITVAAVSL----VLWML-LTGKIWEILAYVTLPVLYILILIPVIFIILRIWSTVRFILLVVLH-GIGN-TFQGGANCIMFVLCTRVV
tr|A0A7L1X4Q8|A0A7L1X4Q8_9AVES VVVLV----SCVL----SFLGSSLLVCTHALLLLYLSLADLLSALSYFYGVLVLQGALSTFSNTSSFFWTMAIALLWC-FHVVSWGVPLGITVAAVAL----LLWML-LTGKVWEILAYVTLPVLYILILIPVIFIILRIWSTVRFILVLVVLH-GIGN-TFQGGANCIMFVLCTRLV
tr|A0A7K6NCC0|A0A7K6NCC0_PEDTO VVVLV----SCVL----SSLGSSLLVCTHALLLLYLSLADLLSALSYFYGVLVLQGALSTFSNTSSFFWTMAIALLLCC-FHVVSWGLPLGITVAAVSL----LLWML-LTGKVWEILAYVTLPVLYILVLIPVIFIILRIWSTVRFILVLVVLH-GIGN-TFQGGANCIMFVLCTRVV
tr|A0A7L0PZV3|A0A7L0PZV3_9AVES VVVLV----SCVL----SFLGSSLLVCTHALLLLYLSLADLLSALSYFYGVLVLQGALSTFSNTSSFFWTMAIAFLLCC-FHAVSWGVPLGITVAAVAL----VLWML-LTGKVWEILAYVTLPVLYILILIPVIFIILRIWSTVRFILLLVVLH-GIGN-TFQGGANCIMFVLCTRVV
tr|A0A7K6W6E6|A0A7K6W6E6_STECA VVVLV----SCVL----SFLGSSLLVCTHALLLLYLSLADLLSALSYFYGVLVLQGALSTFSNTSSFFWTMAIALLLCC-FHVVSWGLPLGITVAAVSL----VLWML-LTGKVWEILAYVTLPVLYILILIPVIFIILRIWSTVRFILVLVVLH-GIGN-TFQGGANCIMFVLCTRVV
tr|A0A7K4YKT9|A0A7K4YKT9_BUCAB AVVLV----SCVL----SFLGSGLLVCTHALLLLYLSLADLLSALSYFYGVLVLQGALSTFSNTSSFFWTMAIALLLCC-FHVVSWGVPLGITVAAVAL----LLWML-LTGKVWEILAYVTLPVLYILILIPVIFIILRIWSTVRFILVLVVLH-GIGN-TFQGGANCIMFVLCTRVV
tr|A0A7K8SNR4|A0A7K8SNR4_9AVES VVVLV----SCVL----SFLGSSLLVCTHALLLLYLSLADLLSALSYFYGVLVLQGALSTFSNTSSFFWTMAIAFLLCC-FHAVSWGLPLGITVAAVSL----VLWML-LTGKVWEILAYVTLPVLYILILIPVIFIILRIWSTVRFVLVLVVLH-GIGN-TFQGGANCIMFVLCTRVV
tr|A0A7L2FZE3|A0A7L2FZE3_NYCGR VIVLV----SCVL----SFLGSSLLVCTHALLLLYLSLADLLSALSYFYGVLVLQGALSTFSNTSSFFWTMAIALLLCC-FHTVSWGVPLGITVAAVAL----VLWML-LTGKVWEILAYVTLPVLYILILIPVIFIILRIWSTVRFILVLVVLH-GIGN-TFQGGANCIMFVLCTRVV
tr|A0A7L0RBY2|A0A7L0RBY2_GLABR ALVLL----SCAL----SFLGSSLLVCTHALLLLYLSLADLLSALSYFYGVLVLQGAMSTFSNTSSFFWTMAIALLLCC-FHIVSWGLPLGITVAAVSL----VLWML-LTGKVWEILAYVILPVLYILILIPVIFIILRVWSTVRFILVLVVLH-GIGN-TFQGGANCIMFVLCTRVV
tr|A0A7L0CYF1|A0A7L0CYF1_9CHAR VVVLV----SCVL----SFLGSSLLVCTHALLLLYLSLADLLSALSYFYGVLVLQGALSTFSNTSSFFWTMAIALLLCC-FHVVSWGVPLGITVAAVAL----LLWML-LTGKVWEILAYVTLPVLYILILIPVIFIILRIWSTVRFILVLVVLH-GIGN-TFQGGANCIMFVLCTRLV
tr|A0A7K4J3P6|A0A7K4J3P6_GEOCA VAVLV----SCVL----SFLGSSLLVCTHALLLLYLSLADLLSALSYFYGVLVLQGALSTFSNTSSFFWTMAIALLLCC-FHGVSWGLPLGITVAAVSL----VLWML-LTGKVWEILAYVTLPVLYILILIPVIFIILRIWSTVRFILVLVVLH-GIGN-TFQGGANCIMFVLCTRAV
tr|A0A7K7UH61|A0A7K7UH61_9CHAR AVVLV----SCAL----SLLGSGLLVGTHALLLLYLSLADLLSALSYFYGVLVLQGALSTFANTSSFFWTMAVALLLCC-FHVVSWGVPLGITVAAVAL----VLWML-LTGKVWEILAYVTLPVLYILILIPVIFIILRIWSTVRFILVLVVLH-GIGN-TFQGGANCIMFVLCTRVV
tr|A0A7L1L632|A0A7L1L632_HIMHI AVVLV----SCVL----SFLGSGLLVGTHALLLLYLSLADLLSALSYFYGVLVLQGALSTFSNTSSFFWTMAVALLLCC-FHVVSWGLPLGITVAAVSL----VLWML-LTGKVWEILAYVTLPVLYILILIPVIFIILRIWSTVRFILVLVVLH-GIGN-TFQGGANCIMFVLCTRVV
tr|A0A7K8K1V0|A0A7K8K1V0_9GRUI VVVLI----SCVL----SFLGSSLLVCTHALLLLYLSLADLLSALSYFYGVLVLQGALSTFSNTSSFFWTMAIALLLCG-FHVVSWGVPLGITVAAVAL----VLWML-LTGKVWEILAYVTLPVLYILILIPVIFIVLRIWSTVRFILVLVVLH-GIGN-TFQGGANCIMFVLCTRVV
tr|A0A7K8KNS1|A0A7K8KNS1_9GRUI VVVLI----SCVL----SFLGSSLLVCTHALLLLYLSLADLLSALSYFYGVLVLQGALSTFSNTSSFFWTMAIALLLCG-FHIVSWGLPLGITVAAVSL----VLWML-LTGKVWEILAYVTLPVLYILILIPVIFIVLRIWSTVRFILVLVVLH-GIGN-TFQGGANCIMFVLCTRVV
tr|A0A663LSS8|A0A663LSS8_ATHCN VVVLV----SCAL----SFLGSSLLVCTHALLLLYLSLADLLSALSYFYGVLVLQGAMSTFSNTSSFFWTMAIALLLCC-FHVVSWGVPLGITVAAVAL----VLWML-LTGKVWEILAYVILPVLYILILIPVIFIILRVWSTVRFILVLVVLH-GIGN-TFQGGANCIMFVLCTRVV
tr|A0A7K9T4N3|A0A7K9T4N3_9AVES VVVLV----SCVL----SFLGSSLLVCTHALLLLYLSLADLFSALSYFYGVLVLQGALSTFSNTSSFFWTMAIAILLCC-FHVVSWGLPLGITVAAVSL----VLWML-LTGKVWEILAYVTLPVLYILILIPVIFIILRIWSTVRFILVLVVLH-GIGN-TFQGGANCIMFVLCTRVV
tr|A0A7L0GHN9|A0A7L0GHN9_HERCA AVVLV----SCVI----SFLGSSLLVCTHALLLLYLSLADLLSALSYFYGVLVLQGALSTFSNTSSFFWTMAIALLLCC-FHVVSWGVPLGITVAAVAL----LLWML-LTGKVWEILAYVTLPVLYILILIPVIFIILRIWSTVRFILVLVVLH-GIGN-TFQGGANCIMFVLCTRVV
tr|A0A7K6S823|A0A7K6S823_9GRUI VVVLV----SCVL----SFLGSSLLVCTHALLLLYLSLADLLSALSYFYGVLVLQGALSTFSNTSSFFWTMAVALLLCC-FHVVSWGLPLGITVAAVSL----VLWML-LTGKVWEILAYVTLPVLYILILIPVIFIILRIWSTVRFILVLVVLH-GIGN-TFQGGANCIMFVLCTRVV
tr|A0A7L0H9A8|A0A7L0H9A8_AREIN VVVLV----SCVL----SFLGSSLLVCTHALLLLYLSLADLLSALSYFYGVLVLQGALSTFSNTSSFFWTMAVALLLWC-FHAVSWGVPLGITVAAVAL----LLWML-LTGKVWEILAYVTLPVLYILILIPVIFIILRIWSTVRFILVLVVLH-GIGN-TFQGGANCIMFVLCTRVV
tr|A0A7K4SVJ7|A0A7K4SVJ7_9CHAR VVVLV----SCVL----SFLGSSLLVCTHALLLLYLSLADLLSALSYFYGVLVLQGALSTFSNTSSFFWTMAVALLLCC-FHVVSWGLPLGITVAAVSL----VLWML-LTGKAWEILAYVTLPVLYILVLIPVIFIILRIWSTVRFILVLVVLH-GIGN-TFQGGANCIMFVLCTRVV
tr|A0A7L3GII4|A0A7L3GII4_9AVES AVVLV----SCVL----SFLGSSLLVCTHALLLLYLSLADLLSALSYFYGVLVLQGALSTFANTSSFFWTMAIALLLCC-FHVVSWGVPLGITVAAVAL----VLWML-LTGKVWEILAYVTLPVLYILILIPVIFIFLRIWSTVRFILVLVVLH-GIGN-TFQGGANCIMFVLCTRAV
tr|A0A851Q5Y8|A0A851Q5Y8_ANHAN VVVLV----SCVL----SFLGSSLLVCTHALLLLYLSLADLLSALSYFYGVLVLQGALSTFANTSSFFWTMAIALLLCC-FHVVSWGLPLGITVAAVSL----VLWML-LTGKVWEILAYVTLPALYILILIPVIFIFLRIWSTVRFILVLVVLH-GIGN-TFQGGANCIMFVLCTRAV
tr|A0A7L4HW88|A0A7L4HW88_SCOUM VIVLL----SCVL----SFLGSSLLVCTHALLLLYLSLADLLSALSYFYGVLVLQGALSTFSNTSSFFWTMAVALLLCC-FHVVSWGVPLGITVAAVAL----VLWML-LTGKVWEILAYVTLPVLYILILIPVIFIILRIWSTVRFILVLVVLH-GIGN-TFQGGANCIMFVLCTRVV
tr|A0A850WM85|A0A850WM85_FREMA VVVLV----SCVL----SFLGSSLLVCTHALLLLYLSLADLLSALSYFYGVLVLQGALSTFSNTSSFFWTMAVALLLCC-FHVVSWGLPLGITVAAVSL----VLWML-LTGKVWEILAYVTLPVLYILILIPVIFIILRIWSTVRFILVLVVLH-GIGN-TFQGGANCIMFVLCTRVV
tr|A0A7L3FFZ2|A0A7L3FFZ2_9GRUI VVVLV----SCVL----SFLGSSLLVCTHALLLLYLSLADLFSALSYFYGVLVLQGALSTFSNTSSFFWTMAIALLLCC-FHVVSWGVPLGITVAAVAL----VLWML-LTGKVWEILAYVTLPVLYILILIPVIFIILRIWSTVRFILVLVVLH-GIGN-TFQGGANCIMFVLCTRVV
tr|A0A851AAC3|A0A851AAC3_SULDA VVVLV----SCVL----SFLGSSLLVCTHALLLLYLSLADLLSALSYFYGVLVLQGALSTFSNTSSFFWTMAIALLLCC-FHVVSWGLPLGITVAAVSL----VLWML-LTGKVWEILAYVTLPVLYILILIPVIFIILRIWSTVRFILVLVVLH-GIGN-TFQGGANCIMFVLCTRAV
tr|A0A7K8PDV3|A0A7K8PDV3_COCCO VVVLV----SCVL----SFLGSSLLVCTHALLLLYLSLADLLSALSYFYGVLVLQGALSTFSNTSSFFWTMAIALLLCC-FHAVSWGVPLGITVAAVAL----VLWML-LTGKVWEILAYVTLPVLYILILIPVIFIILRIWSTVRFILVLVVLH-GIGN-TFQGGANCIMFVLCTRVV
tr|A0A7L0ALK9|A0A7L0ALK9_9AVES VVVLV----SCVL----SFLGSSLLVCTHALLLLYLSLADLLSALSYFYGVLVLQGALSTFSNTSSFFWTMAIALLLCC-FHIVSWGLPLGITVAAVSL----VLWML-LTGKVWEILAYVTLPVLYILILIPVIFIILRIWSTVRFILVLVVLH-GIGN-TFQGGANCIMFVLCTRVV
tr|A0A7L0SJ14|A0A7L0SJ14_PODPO VVVLV----SCVL----SFLGSSLLVCTHALLLLYLSLADLLSALSYFYGVLVLQGALSTFSNTSSFFWTMAIALLLCC-FHVVSWGVPLGITVAAVAL----VLWML-LTGKVWEILAYVALPVLYILILIPVIFIILRIWSTVRFILVLVVLH-GIGN-TFQGGANCIMFVLCTRVV
tr|A0A7L2UMK3|A0A7L2UMK3_BALRX VVVLV----SCVL----SFLGSSLLVCTHALLLLYLSLADLLSALSYFYGVLVLQGALSTFSNTSSFFWTMAIALLLCC-FHVVSWGLPLGITVAAVSL----VLWML-LTGKVWEILAYVTLPVLYILILIPVIFIILRIWSTVRFILVLVVLH-GIGN-TFQGGANCIMFVLCTRVV
tr|A0A7K8U9P8|A0A7K8U9P8_OCEOC VVVLV----SCVL----SFLGSSLLVCTHALLLLYLSLADLLSALSYFYGVLVLQGALSTFSNTSSFFWTMAIALLLCC-FHGVSWGVPLGITVAAVAL----VLWML-LTGKVWEILAYVTLPVLYILILIPVIFIILRIWSTVRFILVLVVLH-GIGN-TFQGGANCIMFVLCTRVV
tr|A0A7L3YN65|A0A7L3YN65_FREGA VVVLV----SCVL----SFLGSSLLVCTHALLLLYLSLADLLSALSYFYGVLVLQGALSTFSNTSSFFWTMAIALLLCC-FHVVSWGLPLGITVAAVSL----VLWML-LTGKVWEILAYVTLPVLYILILIPVIFIILRIWSTVRFILVLVVLH-GIGN-TFQGGANCIMFVLCTRVV
tr|A0A7L0F5E5|A0A7L0F5E5_CORCN VVVLL----SCVL----SFLGSSLLVCTHALLLLYLSLADLLSALSYFYGVLVLQGALSTFSNTSSFFWTMAIALLLCC-FHVVSWGVPLGITVAAVAL----VLWML-LTGKVWEILAYVTLPVLYILILIPVIFIILRIWSTVRFILVLVVLH-GIGN-TFQGGANCIMFVLCTRVV
tr|A0A851LHY2|A0A851LHY2_CORCR VVVLL----SCVL----SFLGSSLLVCTHALLLLYLSLADLLSALSYFYGVLVLQGALSTFSNTSSFFWTMAIALLLCC-FHVVSWGLPLGITVAAVSL----VLWML-LTGKVWEILAYVTLPVLYILILIPVIFIILRIWSTVRFILVLVVLH-GIGN-TFQGGANCIMFVLCTRVV
tr|A0A7K9M5F5|A0A7K9M5F5_OCETE VVVLV----SCVL----SFLGSSLLVCTHALLLLYLSLADLLSALSYFYGVLVLQGALSTFSNTSSFFWTMAIALLLCC-FHVVSWGVPLGITVAAVAL----VLWML-LTGKVWEILAYVTLPVLYILILIPVIFIILRIWSTVRFILVLVVLH-GIGN-TFQGGANCIMFVLCTRVV
tr|A0A7L2W6B9|A0A7L2W6B9_PANHA VVVLV----SCVL----SFLGSSLLVCTHALLLLYLSLADLLSALSYFYGVLVLQGALSTFSNTSSFFWTMAVALLLCC-FHVVSWGLPLGITVAAVSL----VLWML-LTGKVWEILAYVTLPVLYILILIPVIFIILRIWSTVRFILVLVVLH-GIGN-TFQGGANCIMFVLCTRAV
tr|A0A7L1T047|A0A7L1T047_ARAGA VVVLV----SCVL----SFLGSSLLVCTHALLLLYLSLADLLSALSYFYGVLVLQGALSTFSNTSSFFWTMAIALLLCC-FHVVSWGVPLGITVAAVAL----VLWML-LTGKVWEILAYVTLPVLYILILIPVIFIILRIWSTVRFILVLVVLH-GIGN-TFQGGANCIMFVLCTRVV
```

```
tr|A0A850U114|A0A850U114_GRUAM VVVLV----SCVL----SFLGSSLLVCTHALLLLYLSLADLLSALSYFYGVLVLQGALSTFSNTSSFFWTMAIALLLCC-FHVVSWGLPLGITVAAVSL----VLWML-LTGKVWEILAYVTLPVLYILILIPVIFIILRIWSTVRFILVLVVLH-GIGN-TFQGGANCIMFVLCTRVV
tr|A0A7L3JD34|A0A7L3JD34_THACH VVVLV----SCVL----SFLGSGLLVCTHALLLLYLSLADLLSALSYFYGVLVLQGALSTFSNTSSFFWTMAIALLLCC-FHVVSWGVPLGITVAAVAL----VLWML-LTGKVWEILAYVTLPVLYILILIPVIFIILRIWSTVRFILVLVVLH-GIGN-TFQGGANCIMFVLCTRVV
tr|A0A7K7N8D4|A0A7K7N8D4_HALAL VVVLA----SCVL----SFVGSSLLVCTHALLLLYLSLADLLSALSYFYGVLVLQGALSTFSNTSSFFWTMAIALLLCC-FHVVSWGLPLGITVAAVSL----VLWML-LTGKVWEILAYVTLPVLYILILIPVIFIILRIWSTVRFILVLVVLH-GIGN-TFQGGANCIMFVLCTRVV
tr|A0A663F470|A0A663F470_AQUCH VVVLV----SCVL----SFVGSSLLVCTHALLLLYLSLADLLSALSYFYGVLVLQGALSTFSNTSSFFWTMAIALLLCC-FHVVSWGVPLGITVAAVAL----VLWML-LTGKVWEILAYVTLPVLYILILIPVIFIILRIWSTVRFILVLVVLH-GIGN-TFQGGANCIMFVLCTRVV
tr|A0A7L0BPI0|A0A7L0BPI0_9AVES VVVLV----SCVL----SFVGSSLLVCTHALLLLYLSLADLLSALSYFYGVLVLQGALSTFSNTSSFFWTMAIALLLCC-FHVVSWGLPLGITVAAVSL----VLWML-LTGKVWEILAYVTLPVLYILILIPVIFIILRIWSTVRFILVLVVLH-GIGN-TFQGGANCIMFVLCTRVV
tr|A0A7L3ZM84|A0A7L3ZM84_9AVES VVVLV----SCVL----SFVGSSLLVCTHALLLLYLSLADLLSALSYFYGVLVLQGALSTFSNTSSFFWTMAIALLLCC-FHVVSWGVPLGITVAAVAL----VLWML-LTGKVWEILAYVTLPVLYILILIPVIFIILRIWSTVRFILVLVVLH-GIGN-TFQGGANCIMFVLCTRVV
tr|A0A7L2HRV5|A0A7L2HRV5_SAGSE VVVLV----SCAL----SFLGSSLLVCTHALLLLYLSLADLLSALSYFYGVLVLQGALSTFSNTSSFFWTMAIALLLCC-FHVVSWGLPLGITVAAVSL----VLWML-LTGKVWEILAYVTLPVLYILILIPVIFIILRIWSTIRFILVLVVLH-GIGN-TFQGGANCIMFVLCTRVV
tr|A0A7L3CDS3|A0A7L3CDS3_PELUR VVVLV----SCVL----SFLGSSLLVCTHALLLLYLSLADLLSALSYFYGVLVLQGALSTFSNTSSFFWTMAVALLLCC-FHVVSWGVPLGITVAAVAL----VLWML-LTGKVWEILAYVTLPVLYILILIPVIFIILRIWSTVRFILVLVVLH-GIGN-TFQGGANCIMFVLCTRVV
tr|A0A7L3XM08|A0A7L3XM08_9AVES VVVLV----SCVL----SFLGSSLLVCTHALLLLYLSLADLLSALSYFYGVLVLQGALSTFSNTSSFFWTMAIALLLCC-FHVVSWGLPLGITVAAVSL----VLWML-LTGKVWEILAYVTLPVLYILILIPVIFIILRIWSTVRFILVLVVLH-GIGN-TFQGGANCIMFVLCTRVV
tr|A0A7L1Q8E3|A0A7L1Q8E3_9PASS APVLV----SCAL----SCAGSALLLCTQARLLLYLSLADLLSALSYLYGVLVLQGALSTFANTSSFFWTMAIALLLWS-FHAVSWGVPLGITVAAVAL----LLWML-LAGKAWEMLAYVTLPVLYILILIPVIFIFLRIWSTVRFVLVLVVLH-GIGN-TFQGGANCILFVLGTREV
tr|A0A7L2KDK2|A0A7L2KDK2_9PASS VAVLL----SCAV----SCLGSVLLLLSQARLLLYLSLADLLSALSYFYGVLVLQGAVSTFANTSSFFWTMAVALLLWA-FHAVSWGLPLGITVAAVSL----LLWML-LAGKAWEILAYVTLPVLYILILIPIIFIFLRVWSTVRFVLLLVVLH-GIGN-TFQGGANCILFVLCTRVV
tr|A0A7L0LBF4|A0A7L0LBF4_9SYLV GAVLV----SCAL----SCLGSALLLCTHARLLLYLSLADLLSALSYFYGVLVLQGALSTFANTSSFFWTMAIALLLWC-FHAVSWGVPLGITVAAVAL----LLWML-LTGKLWEMLAYVTLPVLYILTLIPLIFIFLRVWSTVRFVLVLVVLH-GFGN-TFQGGANCILFVLCTRAV
tr|A0A7K6R5F0|A0A7K6R5F0_9PASS VLVLL----SCAL----SCAGSALLLCTHARLLLYLSLADLLSALSYFYGVLVLQGALSTFANTSSFFWTMAVALALWA-FHAVSWGLPLGITVAAVSL----LLWML-LTGKVWEILAYVTLPVLYILILIPVIFIFLRIWSTVRFVLLLVVLH-GIGN-TFQGGANCIMFVLCTRVV
tr|A0A7L2BNP2|A0A7L2BNP2_9PASS GAVLV----SCAL----SCAGSALLLCSQARLLLYLSLADLLSALSYFYGALVLQGALSTFANTSSFFWTMAIALLLWC-FHAVSWGVPLGITVAAVAL----LLWML-LAGKAWEMLAYVTLPVLYILILIPLIFISLRIWSTVRFILVLVVLH-GIGN-TFQGGANCILFVLCTRAV
tr|A0A7K6GWL0|A0A7K6GWL0_9PASS VAVLV----SCAL----SCVGSALLLCTHAQLLLYLSLADLLSALSYFYGVLVLQGALSTFANTSSFFWTMAIALVLWC-FHAVSWGLPLGITVAAVSL----LLWML-LAGKLWEMLAYVTLPVLYILILIPLIFIFLRIWSTVRFVLVLVVLH-GIGN-TFQGGANCIMFVLCTGVV
tr|A0A7K7G8G4|A0A7K7G8G4_ERIRU SVVLL----SCAV----SCVGSALLLCTHACLLLHLSLADLLSALSYFYGVLVLQGALSTFANTSSFFWTTAIALWFWG-FHAVSWGVPLGITVAAVAL----LLWML-LTGKLWEMLAYVTLPVLYILILIPVIFIFLRIWSTVRFILVLVVLH-GIGN-TFQGGANCILFVLCTGVV
tr|A0A7K5R1W9|A0A7K5R1W9_9PASE GAVLL----SCAL----SCAGSALLLGSQARLLLHLSLADLLSALSYFYGVLVLQGAVSTFANTSSFFWTMAVALLLWG-FHAVSWGLPLGITVAAVSL----LLWML-LTGKLWEILAYVTLPVLYILILIPIIFIFLRIWSTVRFVLVLVVLH-GVGN-TFQGGANCIMFVLCTRVV
tr|A0A852AKA3|A0A852AKA3_CALOR VPVLL----SCAL----SCAGSALLLCSQALLLLHLSLADLLSALSYFYGVLVLQGALSTFANTSSFFWTMAIALLLWG-FHAVSWGVPLGITVAAVAL----LLWML-LAGKLWEILAYVTLPVLYLLILIPLIFIFLRIWSTVRFVLVLVVLH-GIGN-TFQGGANCILFVLCTRAV
tr|A0A7L0QLR1|A0A7L0QLR1_SETKR VPVLL----SCAL----SCAGSALLLCSQALLLLHLSLADLLSALSYFYGVLVLQGALSTFANTSSFFWTMAIALLLWG-FHAISWGLPLGITVAAVSL----LLWML-LAGKLWEILAYITLPVLYLLILIPLIFIFLRIWSTVRFVLVLVVLH-GIGN-TFQGGANCILFVLCTRAV
tr|A0A7K4Q456|A0A7K4Q456_MELMO VPVLL----SCAL----SCAGSALLLCSQALLLLHLSLADLLSALSYFYGVLVLQGALSTFANTSSFFWTMAIALLLWG-FHAVSWGVPLGITVAAVAL----LLWML-LAGKLWEILAYVTLPVLYLLILIPLIFIFLRIWSTVRFVLVLVVLH-GIGN-TFQGGANCILFMLCTRAV
tr|A0A7K8SBA5|A0A7K8SBA5_9PASS VPVLL----SCAL----SCAGSALLLCSQALLLLHLSLADLLSALSYFYGVLVLQGALSTFANTSSFFWTMAIALLLWG-FHAVSWGLPLGITVAAVSL----LLWML-LAGKLWEILAYVTLPVLYLLILIPLIFIFLRIWSTVRFVLVLVVLH-GIGN-TFQGGANCILFVLCTRAV
tr|A0A7L3VEY4|A0A7L3VEY4_MOLAT VPVLL----SCAL----SCAGSALLLCSQALLLLHLSLADLLSALSYFYGVLVLQGALSTFANTSSFFWTMAIALLLWG-FHAVSWGVPLGITVAAVAL----LLWML-LAGKLWEILAYVTLPVLYLLILIPLIFIFLRIWSTVRFVLVLVVLH-GIGN-TFQGGANCILFVLCTRAV
tr|A0A7K7RET7|A0A7K7RET7_9PASS VPVLL----SCAL----SCAGSVLLLCSQALLLLHLSLADLLSALSYFYGVLVLQGALSTFANTSSFFWTMAIALLLWG-FHAVSWGLPLGITVAAVSL----LLWML-LAGKLWEILAYVTLPVLYLLILIPLIFIFLRIWSTVRFVLVLVVLH-GIGN-TFQGGANCILFVLCTRAV
tr|A0A7K5MAV1|A0A7K5MAV1_CARCD VPVLL----SCAL----SCAGSALLLCSQALLLLHLSLADLLSALSYFYGVLVLQGALSTFANTSSFFWTMAIALLLWG-FHAVSWGVPLGITVAAVAL----LLWML-LAGKLWEILAYVTLPVLYLLILIPLIFIFLRVWSTVRFVLVLVVLH-GIGN-TFQGGANCILFVLCTRAV
tr|A0A6I9HPS3|A0A6I9HPS3_GEOFO VPVLL----SCAL----SCAGSALLLCSQALLLLHLSLADLLSALSYFYGVLVLQGALSTFANTSSFFWTMAIALLLWG-FHAVSWGLPLGITVAAVSL----LLWML-LAGKLWEILAYVTLPVLYLLILIPLIFIFLRIWSTVRFVLVLVVLH-GIGN-TFQGGANCILFVLCTRAV
tr|A0A8C3N8J7|A0A8C3N8J7_GEOPR VPVLL----SCAL----SCAGSALLLCSQALLLLHLSLADLLSALSYFYGVLVLQGALSTFANTSSFFWTMAIALLLWG-FHAVSWGVPLGITVAAVAL----LLWML-LAGKLWEILAYVTLPVLYLLILIPLIFIFLRIWSTVRFVLVLVVLH-GIGN-TFQGGANCILFVLCTRAV
tr|A0A7K7IZA0|A0A7K7IZA0_LOXCU VPVLL----SCAL----SCAGSALLLCSQARLLLHLSLADLLSALSYFYGVLVLQGALSTFANTSSFFWTMAIALLLWG-FHAVSWGLPLGITVAAVSL----LLWML-LTGKLWEILAYVTLPVLYILILIPLIFIFLRIWSTVRFVLVLVVLH-GIGN-TFQGGANCILFVLCTRAV
tr|A0A7K9G6B5|A0A7K9G6B5_LOXLE VPVLL----SCAL----SCAGSALLLCSQARLLLHLSLADLLSALSYFYGVLVLQGALSTFANTSSFFWTMAIALLLWG-FHAVSWGVPLGITVAAVAL----LLWML-LTGKLWEILAYVTLPVLYILILIPLIFIFLRIWSTVRFVLVLVVLH-GIGN-TFQGGANCILFVLCTRAV
tr|A0A8C9NWA0|A0A8C9NWA0_SERCA AVLLL----PCTC---------------SARLLLHLSLADLLSALSYFYGVLVLQGALSTFANTSSFFWTMAIALLLWG-FHAVSWGLPLGITVAAVSL----LLWML-LTGKLWEILAYVTLPVLYILILIPLIFIFLRIWSTVRFVLVLVVLH-GIGN-TFQGGANCILFVLCTRAV
tr|A0A7K7ECV2|A0A7K7ECV2_9SYLV VAVLL----SCAV----SCGGSALLLASQARLLLYLSLADLLSALSYFYGVLVLQGALSTFANTSSFFWTMAIALLLWA-FHAVSWGVPLGITVAAVAL----LLWML-LTGKAWEMLAYVTLPVLYILILIPLIFIFLRIWSTVRFVLVLVVLH-GIGN-TFQGGANCIMFVLCTRVV
tr|A0A7L2P2W4|A0A7L2P2W4_PYCJO GAVLV----SCSL----SCLGSALLLCSHALLLLHLSLADLLSALSYFYGALVLQGALSTFANTSSFFWTMAIALLLWG-FHAVSWGLPLGITVAAVSL----LLWML-LTGKLWEILAYVTLPVLYILIFIPVIFIFLRVWSTVRFVLVLVVLH-GIGN-TFQGGANCIMFVLCTRVV
tr|A0A7K4UD90|A0A7K4UD90_9SYLV GAVLV----SCAL----SCAGSALLLCSQARLLLYLSLADLLSALSYFYGVLVLQGALSTFANTSSFFWTMAIALLLWG-FHAVSWGVPLGITVAAVAL----LLWML-LTGKVWEILAYVTLPVLYILILIPLIFIFLRVWSTVRFVLVLVVLH-GIGN-TFQGGANCIMFVLCTRVV
tr|A0A7L1BSX9|A0A7L1BSX9_9PASS GAVLV----SCAL----SCAGSALLLGSHARLLLYLSLADLLSALSYFYGVLVLQGALSTFANTSSFFWTMAIALLLWG-FHAVSWGLPLGITVAAVSL----LLWML-LAGKVWEILAYVTLPVLYILILIPVIFILLRIWSTVRFILVLVVLH-GIGN-TFQGGANCIMFVLCTRVV
tr|A0A7L2A254|A0A7L2A254_LEILU GAVLV----SCAL----SCAGSALLLGSHARLLLYLSLADLLSALSYFYGVLVLQGALSTFANTSSFFWTMAVALLLWG-FHAVSWGVPLGITVAAVAL----LLWML-LAGKVWEILAYVTLPVLYVLILIPVIFIFLRIWSTVRFVLVLVVLH-GIGN-TFQGGANCIMFVLCTRVV
tr|A0A7L1RRL1|A0A7L1RRL1_9PASS CAVLL----SCAL----SCAGSALLLCSQARLLLCLSLADLLSALSYFYGVLVLQGALSTFANTSSFFWTMAVALLLWC-FHAVSWGLPLGITVAAVSL----LLWML-LTGKVWEILAYVTLPVLYILILIPVIFILLRIWSTVRFVLVLVVLH-GIGN-TFQGGANCIMFVLCTRAV
tr|A0A7K6MT25|A0A7K6MT25_PANBI GAVLV----SCAL----SCAGSALLLCTHAWLLLYLSLADLLSALSYFYGVLVVQGALSTFANTSSFFWTMAIALLLWC-FHAVSWGVPLGITVAAVAL----LLWML-LTGKVWEILAYVTLPVLYILILIPVIFIFLRIWSTVRFVLVLVVLH-GIGN-TFQGGANCIMFVLCTRVV
tr|A0A852HSV2|A0A852HSV2_9PASS GAVLV----SCAL----SCAGSALLLCTHARLLLYLSLADLLSALSYFYGVLVLQGALSTFANTSSFFWTMAIALLLWC-FHAVSWGLPLGITVAAVSL----LLWML-LTGKVWEILAYVTLPVLYILILIPVIFIFLRIWSTVRFVLVLVVLH-GIGN-TFQGGANCILFVLCTRVV
tr|A0A851J9J3|A0A851J9J3_9PASS CAVLL----SCAL----SCAGSALLLGAQARLLLYLSLADLLSALSYFYGVLVLQGALSTFANTSSFFWTVAIALLLWC-FHAVSWGVPLGITVAAVAL----LLWML-LTGKVWEILAYVTLPVLYILILIPVIFIFLRVWSTVRFVLVLVVLH-GIGN-TFQGGANCIVFVLCTRVV
tr|A0A7K7PT00|A0A7K7PT00_ACRAR AAVLL----SCAL----SCAGSALLLCTHARLLLHLSLADLLSALSYFYGVLVLQGALSTFANTSSFFWTMAIALLLWC-FHAVSWGLPLGITVAAVSL----LLWML-LTGKVWEILAYVTLPVLYILILIPVIFIFLRIWSTVRLVLVLVVLH-GIGN-TFQGGANCIMFVLCTRVV
tr|A0A7L2MJF9|A0A7L2MJF9_9PASS GAVLL----SCAL----SCAGSALLLCTHARLLLYLSLADLLSALSYFYGVLVLQGALSTFANTSSFFWTMAIALLLWC-FHAVSWGVPLGITVAAVAL----LLWML-LTGKVWEILAYVTLPVLYILILIPVIFIFLRIWSTVRFVLVLVVLH-GIGN-TFQGGANCIMFVLCTRVV
tr|A0A7L4E396|A0A7L4E396_HIRRU GAVLV----SCAL----SCLGSALLLGSHARLLLHLSLADLLSALSYFYGVLVLQGALSTFANTSSFFWTMAIALLLWS-FHAVSWGLPLGITVAAVSL----LLWML-LTGKVWEILAYVTLPVLYVLILIPVIFIFLRVWSTVRFVLVLVVLH-GIGN-TFQGGANCIMFVLCTRLV
tr|A0A7K5Q3X4|A0A7K5Q3X4_9CORV GAVLL----SCAL----SCAGSALLLCTHARLLLYLSLADLLSALSYFYGVLVLQGALSTFANTSSFFWTMAIALLLWG-FHAVSWGVPLGITVAAVAL----LLWML-LTGKLWEMLAYVTLPVLYILILIPVIFIFLRMWSTVRFVLVLVVLH-GIGN-TFQGGANCIMFVLCTRVV
tr|A0A7K5VYT7|A0A7K5VYT7_9SYLV GPVLV----SCAL----SCAGSALLLCSHARLLLCLSLADLLSALSYFYGVLVLQGALSTFANTSSFFWTMAVALLLWG-FHAVSWGLPLGITVAAVSL----LLWML-LTGKLWEILAYVTLPVLYILILIPVIFILLRMWSTVRFVLVLVVLH-GIGN-TFQGGANCIMFVLCTRVV
tr|A0A218UHS4|A0A218UHS4_9PASE GAVLL----SCAL----SCAGSALLLGSQARLLLHLSLADLLSALSYSYGVLVLQGALSTFANTSSFFWTMAVALLLWG-FHAVSWGVPLGITVAAVAL----LLWML-LTGKVWEILAYVTLPVLYILILIPIIFIFLRIWSTVRFVLVLVVLH-GIGN-TFQGGANCILFVLGTRAV
tr|A0A7K5CHT7|A0A7K5CHT7_MOTAL -AVLL----SCAL----SCAGSALLLCSQALLLLHLSLADLLSALSYFYGVLVLQGALSTFANTSSFFWTMAIALVLWA-FHAVSWGLPLGITVAAVSL----LLWML-LTGKVWEILAYVTLPVLYILILIPVIFIFLRIWSTVRFVLVLVVLH-GVGN-TFQGGANCIMFVLCTRAV
tr|A0A851UD49|A0A851UD49_9PASS GAVLL----SCAL----SCAGSSLLLATHARLLLHLSLADLLSALSYFYGVLVLQGALSTFANTSSFFWTMAIALLLWG-FHAVSWGVPLGITVAAVAL----LLWML-LTGKAWEILAYVTLPVLYILILIPIIFIFLRIWSTVRFILVLVVLH-GIGN-TFQGGANCIMFVLCTRVV
tr|A0A851VWP4|A0A851VWP4_9PASS GAVLL----SCAL----SCAGSALLLLAQARLLLHLSLADLLSALSYCYGVLVLQGALSTFANTSSFFWTMAIALWLWG-FHAVSWGLPLGITVAAVSL----LLWML-LTGKLWEMLAYVTLPVLYILILIPIIFIFLRIWSTVRFILLLVVLH-GIGN-SFQGGANCILFVLCTRAV
tr|A0A7L1E4B3|A0A7L1E4B3_OENON AAVLL------------SCAGSALLLGSHALLLLYLSLADLLSALSYFYGVLVLQGALSTFANTSSFFWTTAIALWLWG-FHAVSWGVPLGITVAAVAL----LLWML-LTGKLWEILAYVTLPVLYILILIPIIFIFLRIWSTVRFILVLVVLH-GIGN-TFQGGANCIMFVLCTRVV
tr|U3JY19|U3JY19_FICAL         AAVLV----SCAL----SCSGSALLLCSHARLLLYLSLADLLSALSYFYGVLVLQGAVSTFANTSSFFWTMAIALWLWG-FHAVSWGLPLGITVAAVSL----LLWML-LTGKLWEILAYVTLPLLYILILIPIIFIFLRIWSTVRFILVLVVLH-GIGN-TFQGGANCIMFVLCTRVV
tr|A0A7K6XUA6|A0A7K6XUA6_9PASE GAVLL----SCAL----SCAGSALLLCTHAQLLLYLSLADLLSALSYFYGVLVLQGALSTFANTSSFFWTMAVALLLWC-FHAVSWGVPLGITVAAVAL----LLWML-LTGKAWEILAYVTLPVLYILILIPAIFIFLRVWSTVRFVLVLLVLH-GIGN-TFQGGANCIMFVLCTRVV
tr|A0A851AZK6|A0A851AZK6_PICGY GAVLV----SCAL----SCVGSALLLCTHAQLLLYLSLADLLSALSYFYGVLVLQGALSTFANTSSFFWTMAIALLLWC-FHAVSWGLPLGITVAAVSL----LLWML-LAGKVWELLAYVTLPVLYILILIPVIFIFLRIWSTVRFILVLVVLH-VSVC-QETRGANCIMFVLCTRVV
tr|A0A7K5INP7|A0A7K5INP7_TOXRE GLVLV----SCAL----SCLGSALLLCSQALLLLYLSLADLLSALSYSYGVLVLQGALSTFANTSSFFWTMAIALLLWG-FHAVSWGVPLGITVAAVAL----LLWML-LAGKVWEILAYVTLPVLYILILIPIIFIFLRIWSTVRFILVLVVLH-GVGN-TFQGGANCILFVLCTRAV
tr|A0A7K8EI09|A0A7K8EI09_LEURO GAVLV----SCSVPGAAPCLGAGPLSLSRCRLLLHLSLADLLSALSYFYGVLVLQGALSTFANTSSFFWTMAIALLLWG-FHAVSWGLPLGITVAAVSL----LLWML-LTGKVWEILAYVTLPVLYILILIPIIFIFLRIWSTVRFILVLVVLH-GIGN-TFQGGANCIMFVLCTRVV
tr|H0YZZ5|H0YZZ5_TAEGU         GAVLL----SCAL----SCAGSALLLGSQARLLLQLSLADLLSALSYFYGVLVLQGALSTFANTSSFFWTMAIALLLWG-FHAVSWGVPLGITVAAVAL----LLWML-LTGKVWEILAYVTLPVLYILILIPVIFIFLRIWSTVRFVLVLVVLH-GIGN-TFQGGANCVLFVLCTRAV
tr|A0A7L2EGX1|A0A7L2EGX1_ANTMN GAVLL----SCAL----SCVGSALLLCTHARLLLYLSLADLLSAVSYFYGVLVLQGALSTFANTSSFFWTMAIALLLCC-FHAVSWGLPLGITVAAVSL----LLWML-LTGKVWEILAYVTLPVLYILILIPVIFIFLRIWSTVRFVLVLVVLH-GVGN-TFQGGANCIMFVLCTRAV
tr|A0A7L0UUS9|A0A7L0UUS9_9PASE GAVLL----SCAL----SCGGSALLLCTQARLLLYLSLADLLSALSYFYGVLVLQGALSTFANTSSFFWTMAIALLLWG-FHAVSWGVPLGITVAAVAL----LLWML-LTGKVWEILAYVTLPVLYILILIPVIFIFLRVWSTVRFVLVLVVLH-GIGN-TFQGGANCIMFVLCTRVV
tr|A0A7L1MPL5|A0A7L1MPL5_BOMGA GAVLV----SCAL----SCVGSALLLCTHAQLLLYLSLADLLSALSYFYGVLVLQGALSTFANTSSFFWTMAIALLLWG-FHAVSWGLPLGITVAAVSL----LLWML-LTGKVWEILAYVTLPVLYVLILIPLIFIFLRIWSTVRFILVLVVLH-GIGN-TFQGGANCIMFVLCTRVV
```

```
tr|A0A852NM53|A0A852NM53_9PASS VAVLV----SCAL----SCVGSALLLCTHAQLLLYLSLADLLSALSYFYGVLVLQGALSTFANTSSFFWTMAIALLLWC-FHAVSWGVPLGITVAAVAL----LLWML-LTGKVWEILAYVTLPVLYILILIPVIFIFLRIWSTVRFILVLVVLH-GIGN-TFQGGANCIMFVLCTRVV
tr|A0A7K4XU96|A0A7K4XU96_REGSA GVVLL----SCAL----SCTGSALLLCTHARLLLHLSLADLLSALSYFYGVLVLQGALSTFANTSSFFWTMAIALLLWG-FHAVSWGLPLGITVAAVSL----LLWML-LTGKVWEILAYVTLPVLYILILIPIIFIFLRVWSTVRFVLVLVVLH-GIGN-TFQGGANCIMFVLCTRVV
tr|A0A7L1W919|A0A7L1W919_9PASS GAVLV----SCAL----SCLGSVLLLCTHAQLLLYLSLADLLSALSYFYGVLVLQGALSTFANTSSFFWTMAIALLLWG-FHAVSWGVPLGITVAAVAL----LLWML-LTGKVWEILAYVTLPVLYILILIPIIFIFLRIWSTVRFILILVVLH-GIGN-TFQGGANCIMFVLCTRVV
tr|A0A7K5DY08|A0A7K5DY08_POLCE GAVLV----SCAL----SCLGSALLLCTHAQLLLYLSLADLLSALSYFYGVLVLQGALSTFANTSSFFWTMAIALLLWG-FHAVSWGLPLGITVAAVSL----LLWML-LTGKVWEILAYVTLPVLYILILIPVIFICLRIWSTVRFILVLVVLH-GIGN-TFQGGANCIMFVLCTRVV
tr|A0A7K7YK15|A0A7K7YK15_THRLU GAVLV----SCAL----SCLGSALLLCTHAQLLLYLSLADLLSALSYFYGVLVLQGALSTFANTSSFFWTMAIALLLWC-FHAVSWGVPLGITVAAVAL----LLWML-LTGKVWEILAYVTLPVLYILILIPVIFICLRIWSTVRFILVLVVLH-GIGN-TFQGGANCIMFVLCTRVV
tr|A0A7L2QEW6|A0A7L2QEW6_9PASS GAVLL----SCAL----SCAGSALLLCTHARLLLHLSLADLLSALSYFYGVLVLQGALSTFANTSSFFWTMAIALLLWG-FHAVSWGLPLGITVAAVSL----LLWML-LTGKVWEMLAYVTLPVLYLLILIPVIFIFLRIWSTVRFVLVLVVLH-GIGN-TFQGGANCILFVLCTRAV
tr|A0A852G007|A0A852G007_PEUTA GAVLL----SCAL----SCAGSALLLCSQAQLLLHLSLADLLSALSYFYGVLVLQGALSTFANTSSFFWTMAIALLFWG-FHAVSWGVPLGITVAAVAL----LLWML-LTGKAWEILAYVTLPVLYILILIPVIFIFLRIWSTVRFVLVLVVLH-GIGN-TFQGGANCIMFVLCTRAV
tr|A0A7L0Z1Q0|A0A7L0Z1Q0_9PASE GAVLA----SCAL----SCAGSALLLCTHAQLLLHLSLADLLSALSYFYGVLVLQGALSTFANTSSFFWTMAVALLLWG-FHAVSWGLPLGITVAAVSL----LLWML-LTGKVWEILAYVTLPVLYILILIPIIFIFLRIWSTVRFVLVLVVLH-GIGN-TFQGGANCIMFVLCTRVV
tr|A0A851KL00|A0A851KL00_VIDCH GAVLL----SCAL----SCAGSALLLCTHAQLLLHLSLADLLSALSYFYGVLVLQGALSTFANTSSFFWTMAIALLLWG-FHAVSWGVPLGITVAAVAL----LLWML-LTGKVWEILAYVTLPVLYILILIPVIFIFLRIWSTVRFVLVLVVLH-GIGN-TFQGGANCIMFVLCTRVV
tr|A0A852E2C6|A0A852E2C6_VIDMA --VLL----SCAL----SCAGSALLLCTHAQLLLHLSLADLLSALSYFYGVLVLQGALSTFANTSSFFWTMAIALLLWG-FHAVSWGLPLGITVAAVSL----LLWML-LTGKVWEILAYVTLPVLYILILIPVIFIFLRIWSTVRFVLVLVVLH-GIGN-TFQGGANCIMFVLCTRVV
tr|A0A7L2J498|A0A7L2J498_CINMU WAVLL----SCCL----SCLGSALLLCTHARLLLYLSLADLLSALSYFYGVLVLQGALSTFANTSSFFWTMAIALLLWG-FHAVSWGVPLGITVAAVAL----LLWML-LTGKVWEILAYVTLPVLYVLILIPIIFIFLRIWSTVRFILVLVVLH-GIGN-TFQGGANCIMFVLCTRVV
tr|A0A850ZJQ1|A0A850ZJQ1_9PASS GAVLA----SCAL----SCLGSALLLCTHAQLLLYLSLADLLSALSYFYGVLVLQGALSTFANTSSFFWTMAIALLLWG-FHAVSWGLPLGITVAAVSL----LLWML-LTGKVWEILAYVTLPVLYILILIPVIFIFLRIWSTVRFVLVLVVLH-GIGN-TFQGGANCIMFVLCTRVV
tr|A0A7L3EPV5|A0A7L3EPV5_9PASS GAVLV----SCAL----SCVGSALLLCTHAQLLLYLSLADLLSALSYFYGVLVLQGALSTFANTSSFFWTMAIALLLWC-FHAVSWGVPLGITVAAVAL----LLWML-LAGKIWEILAYVALPVLYILILIPVIFIFLRVWSTVRFVLVLVVLH-GIGN-TFQGGANCIMFVLCTRVV
tr|A0A7K6EX30|A0A7K6EX30_9PASS VAVLV----SCSL----SCVGSALLLCTHAQLLLYLSLADLLSALSYFYGVLVLQGALSTFANTSSFFWTMAIALLLWG-FHAVSWGLPLGITVAAVSL----LLWML-LTGKMWEILAYVTLPVLYILILIPIIFIFLRIWSTVRFILVLVVLH-GIGN-TFQGGANCIMFVLCTRVV
tr|A0A7K6UQZ5|A0A7K6UQZ5_9PASS GAVLA----SCAL----SCVGSALLLCTHAQLLLYLSLADLLSALSYFYGVLVLQGALSTFANTSSFFWTMAIALLLWC-FHAVSWGVPLGITVAAVAL----LLWML-LTGKAWEILAYVTLPVLYILILIPVIFIFLRIWSTVRFILVLVVLH-GIGN-TFQGGANCIMFVLCTRVV
tr|A0A7K6DS12|A0A7K6DS12_9PASS VAVLV----SCAL----SCVGSALLLCTHARLLLYLSLADLLSALSYFYGVLVLQGALSTFANTSSFFWTMAIALLLWC-FHAVSWGLPLGITVAAVSL----LLWML-LTGKVWEILAYVTLPVLYILILIPVIFIFLRIWSTVRFILVLVVLH-GIGN-TFQGGANCIMFVLCTRVV
tr|A0A7K6HZ66|A0A7K6HZ66_9PASS VAVLV----SCAL----SCVGSALLLCTHAQLLLYLSLADLLSAVSYFYGVLVLQGALSTFANTSSFFWTMAIALLLWC-FHVVSWGVPLGITVAAVAL----LLWML-LTGKVWEILAYVTLPVLYILILIPVIFILLRIWSTVRFILILVVLH-GIGN-TFQGGANCIMFVLCTRVV
tr|A0A7L3II69|A0A7L3II69_9PASS VAVLV----SCAL----SCVGSALLLCTHAQLLLYLSLADLLSALSYFYGVLVLQGALSTFANTSSFFWTMAIALLLWC-FHVVSWGLPLGITVAAVSL----LLWML-LTGKVWEILAYVTLPVLYILILIPVIFIFLRIWSTVRFILVLVVLH-GIGN-TFQGGANCIMFVLCTRVV
tr|A0A852N748|A0A852N748_9PASS GAVLV----SCAL----SCVGSALLLCTHAQLLLYLSLADLLSALSYFYGVLVLQGALSTFANTSSFFWTMAIALLLWG-FHAVSWGVPLGITVAAVAL----LLWML-LTGKVWEILAYVTLPVLYILVLIPVIFIFLRIWSTVRFVLILVVLH-GIGN-TFQGGANCIMFVLCTRLV
tr|A0A7L2TXE6|A0A7L2TXE6_POMRU GAVLV----SCAL----SCVGSALLLCTHAQLLLYLSLADLLSALSYFYGVLVLQGALSTFANTSSFFWTMAIALLLWC-FHAVSWGLPLGITVAAVSL----LLWML-LTGKVWEILAYVTLPVLYILILIPVIFIFLRIWSTVRFVLVLVVLH-GIGN-TFQGGANCIMFVLCTRVV
tr|A0A7K6F874|A0A7K6F874_9CORV GAVLL----SCSL----SCAGSALLLCTHAQLLLCLSLADLLSALSYFYGVLVLQGALSTFANTSSFFWTMAIALLLWC-FHAVSWGVPLGITVAAVAL----LLWML-LTGKVWEILAYVTLPVLYILILIPVIFIFLRIWSTVRFILVLVVLH-GIGN-TFQGGANCIMFVLCTRVV
tr|A0A7K9Q4I6|A0A7K9Q4I6_9CORV GAVLV----SCAL----SCVGSALLLCTHAQLLLYLSLADLLSALSYFYGVLVLQGALSTFANTSSFFWTMAIALLLWG-FHAVSWGLPLGITVAAVSL----LLWML-LTGKVWEILAYVTLPVLYILILIPVIFIFLRIWSTVRFILVLVVLH-GIGN-TFQGGANCIMFVLCTRAV
tr|A0A7K9NBQ8|A0A7K9NBQ8_9CORV GAVLV----SCAL----SCVGSALLLCTHAQLLLYLSLADLLSALSYFYGVLVLQGALSTFANTSSFFWTMAIALLLCC-FHAVSWGVPLGITVAAVAL----LLWML-LTGKVWEMLAYVTLPVLYILILIPVIFIFLRIWSTVRFILVLVVLH-GIGN-TFQGGANCIMFVLCTRVV
tr|A0A7K5KZQ1|A0A7K5KZQ1_VIRAL GAVLV----SCAL----SCVGSALLLCTHAQLLLYLSLADLLSALSYFYGVLVLQGALSTFANTSSFFWTMAIALLLWC-FHAVSWGLPLGITVAAVSL----LLWML-LTGKVWEILAYVALPVLYILVLIPIIFIFLRIWSTVRFILVLVVLH-GIGN-TFQGGANCIMFVLCTRLV
tr|A0A7K8MJ15|A0A7K8MJ15_9CORV GAVLV----SCAL----SCVGSALLLCTHAQLLLYLSLADLLSALSYFYGVLVLQGALSTFANTSSFFWTMAIALLFWC-FHTVSWGVPLGITVAAVAL----LLWML-LTGKVWEILAYVTLPVLYILVLIPVIFIFLRIWSTVRFVLVLVVLH-GIGN-TFQGGANCIMFVLCTRVV
tr|A0A7K7ZD28|A0A7K7ZD28_9PASE GAVLV----SCAL----SCVGSALLLCTHAQLLLYLSLADLLSALSYFYGVLVLQGALSTFANTSSFFWTMAIALLFWC-FHAVSWGLPLGITVAAVSL----LLWML-LTGKVWEILAYVTLPVLYILILIPVIFIFLRIWSTVRFVLVLVVLH-GIGN-TFQGGANCIMFVLCTRVV
tr|A0A7K6JP09|A0A7K6JP09_9PASE GAVLV----SCAL----SCVGSALLLCTHAQLLLYLSLADLLSALSYFYGVLVLQGALSTFANTSSFFWTMAIALLLWC-FHAVSWGVPLGITVAAVAL----LLWML-LTGKVWEILAYVTLPVLYILVLIPVIFIFLRIWSTVRFVLVLVVLH-GIGN-TFQGGANCIMFVLCTRVV
tr|A0A7K9WKL7|A0A7K9WKL7_9PASS GAVLV----SCAL----SCVGSALLLCTHAQLLLYLSLADLLSALSYFYGVLVLQGALSTFANTSSFFWTMAIALLLWG-FHAVSWGLPLGITVAAVSL----LLWML-LTGKVWEILAYVTLPVLYILVLIPVIFIFLRIWSTVRFVLVLVVLH-GIGN-TFQGGANCIMFVLCTRVV
tr|A0A7L2XIR5|A0A7L2XIR5_9PASS GAVLV----SCSL----SCVGSALLLCTHAQLLLYLSLADLLSALSYFYGVLVLQGALSTFANTSSFFWTMAIALLLWC-FHAVSWGVPLGITVAAVAL----LLWML-LTGKVWEILAYVALPVLYILVLIPVIFIFLRIWSTVRFILVLVVLH-GIGN-TFQGGANCIMFVLCTRVV
tr|A0A7K8GHZ2|A0A7K8GHZ2_ORTSP GAVLV----SCAL----SCVGSALLLCTHAQLLLYLSLADLLSALSYFYGVLVLQGALSTFANTSSFFWTMAIALLLWC-FHAVSWGLPLGITVAAVSL----LLWML-LTGKVWEILAYVTLPVLYILILIPVIFIFLRIWSTVRFILVLVVLH-GIGN-TFQGGANCIMFVLCTRVV
tr|A0A7K5S101|A0A7K5S101_LANLU --VLV----SCAL----SCVGSALLLCTHAQLLLYLSLADLLSALSYFYGVLVLQGALSTFANTSSFFWTMAIALLLWC-FHAVSWGVPLGITVAAVAL----LLWML-LTGKVWEILAYVTLPVLYILILIPVIFIFLRIWSTVRFILILVVLH-GIGN-TFQGGANCIMFVLCTRVV
tr|A0A7L3JZH4|A0A7L3JZH4_9PASS GAVLV----SCAL----SCVGSALLLCTHAQLLLYLSLADLLSALSYFYGVLVLQGALSTFANTSSFFWTMAIALLLWC-FHAVSWGLPLGITVAAVSL----LLWML-LTGKVWEILAYVTLPVLYILILIPVIFIFLRIWSTVRFILVLVVLH-GIGN-TFQGGANCIMFVLCTRVV
tr|A0A7L4KMS9|A0A7L4KMS9_9CORV GAVLV----SCAL----SCVGSALLLCTHAQLLLYLSLADLLSALSYFYGVLVLQGALSTFANTSSFFWTMAIALLLWS-FHAVSWGVPLGITVAAVAL----LLWML-LTGKVWEILAYVTLPVLYILILIPVIFIFLRIWSTVRFILVLVVLH-GIGN-TFQGGANCIMFVLCTRVV
tr|A0A7K8B2A7|A0A7K8B2A7_9CORV GAVLV----SCVL----SCVGSALLLCTHAQLLLYLSLADLLSALSYFYGVLVLQGALSTFANTSSFFWTMAIALLLWC-FHAVSWGLPLGITVAAVSL----LLWML-LTGKVWEILAYVTLPVLYILILIPIIFIFLRIWSTVRFILVLVVLH-GIGN-TFQGGANCIMFVLCTRVV
tr|A0A7K8IN08|A0A7K8IN08_9PASS GAVLV----SCAL----SCVGSALLLCTHAQLLLYLSLADLLSALSYFYGVLVLQGALSTFANTSSFFWTMAIALLLWG-FHAVSWGVPLGITVAAVAL----LLWML-LTGKAWEILAYVTLPVLYILVLIPVIFIFLRIWSTVRFILVLVVLH-GIGN-TFQGGANCIMFVLCTRVV
tr|A0A7K7X704|A0A7K7X704_9PASS GAVLV----SCAL----SCVGSALLLCTHAQLLLYLSLADLLSALSYFYGVLVLQGALSTFANTSSFFWTMAIALLLWC-FHAVSWGLPLGITVAAVSL----LLWML-LTGKVWEILAYVTLPVLYILILIPVIFIFLRIWSTVRFILVLVVLH-GIGN-TFQGGANCIMFVLCTRVV
tr|A0A7K6LWV7|A0A7K6LWV7_9CORV GAVLV----SCSL----SCVGSALLLCTHAQLLLYLSLADLLSALSYFYGVLVLQGALSTFANTSSFFWTMAIALLLWC-FHAVSWGVPLGITVAAVAL----LLWML-LTGKVWEILAYVTLPVLYILILIPVIFIFLRIWSTVRFVLVLVVLH-GIGN-TFQGGANCIMFVLCTRVV
tr|A0A7K8HNE3|A0A7K8HNE3_9CORV GAVLV----SCAL----SCVGSALLLCTHAQLLLYLSLADLLSALSYFYGVLVLQGALSTFANTSSFFWTMAIALLLWC-FHAVSWGLPLGITVAAVSL----LLWML-LTGKVWEILAYVTLPVLYILILIPVIFIFLRIWSTVRFVLVLVVLH-GIGN-TFQGGANCIMFVLCTRVV
tr|A0A7K9IU42|A0A7K9IU42_9CORV -AVLV----SCAL----SCVGSALLLCTHAQLLLYLSLADLLSALSYFYGVLVLQGALSTFANTSSFFWTMAIALLLWC-FHAVSWGVPLGITVAAVAL----LLWML-LTGKVWETLAYVTLPVLYILILIPVIFIFLRIWSTVRFILVLVVLH-GIGN-TFQGGANCIMFVLCTRVV
tr|A0A7K5UXQ4|A0A7K5UXQ4_9CORV GAVLV----SCAL----SCVGSALLLCTHAQLLLYLSLADLVSALSYFYGVLVLQGALSTFANTSSFFWTMAIALVLWC-FHAVSWGLPLGITVAAVSL----LLWML-LTGKVWEILAYVTLPVLYILILIPVIFIFLRIWSTVRFILVLVVLH-GIGN-TFQGGANCIMFVLCTRVV
tr|A0A7K6PIV5|A0A7K6PIV5_9CORV GAVLV----SCAL----SCVGSALLLCTHAQLLLYLSLADLLSALSYFYGVLVLQGALSTFANTSSFFWTMAVALLLWC-FHAVSWGVPLGITVAAVAL----LLWML-LTGKVWEILAYVTLPVLYILILIPVIFIFLRIWSTVRFILVLVVLH-GIGN-TFQGGANCIMFVLCTRVV
tr|A0A7L1P5B4|A0A7L1P5B4_ORIOR GAVLV----SCAL----SCVGSALLLCTHAQLLLYLSLADLLSALSYFYGVLVLQGALSTFANTSSFFWTMAIALLLWC-FHAVSWGLPLGITVAAVSL----LLWML-LTGKVWEILAYVTLPVLYILILIPVIFIFLRIWSTVRFVLVLVVLH-GIGN-TFQGGANCIMFVLCTRVV
tr|A0A7K8BNE7|A0A7K8BNE7_9CORV GAVLV----SCAL----SCVGSALLLCTHAQLLLYLSLADLLSALSYFYGVLVLQGALSTFANTSSFFWTMAIALLLWC-FHAVSWGVPLGITVAAVAL----LLWML-LTGKVWEILAYVTLPVLYILILIPVIFIFLRIWSTVRFVLVLVVLH-GIGN-TFQGGANCIMFVLCTRVV
tr|A0A7K8EZC6|A0A7K8EZC6_9CORV GAVLV----SCAL----SCVGSALLLCTHAQLLLYLSLADLLSALSYFYGVLVLQGALSTFANTSSFFWTMAIALLLWC-FHAVSWGLPLGITVAAVSL----LLWML-LTGKVWEILAYVTLPVLYILILIPVIFIFLRIWSTVRFILVLVVLH-GIGN-TFQGGANCIMFVLCTRVV
tr|A0A7L1APW1|A0A7L1APW1_GYMTI GAVLV----SCAL----SCVGSALLLCTHAQLLLYLSLADLLSALSYFYGVLVLQGALSTFANTSSFFWTMAIALLLWC-FHAVSWGVPLGITVAAVAL----LLWML-LTGKVWEILAYVTLPVLYILILIPVIFIFLRIWSTVRFVLVLVVLH-GIGN-TFQGGANCIMFVLCTRVV
tr|A0A7K6IUV9|A0A7K6IUV9_9CORV GAVLV----SCAL----SCVGSALLLCTHAQLLLYLSLADVLSALSYFYGVLVLQGALSTFANTSSFFWTMAIALLLWC-FHAVSWGLPLGITVAAVSL----LLWML-LTGKVWEILAYVTLPVLYILILIPVIFIFLRIWSTVRFVLVLVVLH-GIGN-TFQGGANCIMFVLCTRVV
tr|A0A7K9ZVH2|A0A7K9ZVH2_9CORV GAVLV----SCAL----SCVGSALLLCTHAQLLLYLSLADLLSALSYFYGVLVLQGALSTFANTSSFFWTMAIALLLWC-FHAVSWGVPLGITVAAVAL----LLWML-LTGKVWEILAYVTLPVLYILILIPVIFIFLRIWSTVRFILVLVVLH-GIGN-TFQGGANCIMFVLCTRVV
tr|A0A7K8D3B9|A0A7K8D3B9_9CORV GAVLV----SCAL----SCVGSALLLCTHAQLLLYLSLADLLSALSYFYGVLVLQGALSTFANTSSFFWTMAIALLLWC-FHAVSWGLPLGITVAAVSL----LLWML-LTGKVWEILAYVTLPVLYILILIPVIFIFLRIWSTVRFILVLVVLH-GIGN-TFQGGANCIMFVLCTRVV
tr|A0A8C3GY47|A0A8C3GY47_CORMO GAVLV----SCAL----SCVGSALLLCTHAQLLLYLSLADLLSALSYFYGVLVLQGALSTFANTSSFFWTMAIALLLWC-FHAVSWGVPLGITVAAVAL----LLWML-LTGKVWEILAYVTLPVLYILILIPVIFIFLRIWSTVRFILVLVVLHVGIGN-TFQGGANCIMFVLCTRVV
tr|A0A8U7N9A9|A0A8U7N9A9_CORMO GAVLV----SCAL----SCVGSALLLCTHAQLLLYLSLADLLSALSYFYGVLVLQGALSTFANTSSFFWTMAIALLLWC-FHAVSWGLPLGITVAAVSL----LLWML-LTGKVWEILAYVTLPVLYILILIPVIFIFLRIWSTVRFILVLVVLH-GIGN-TFQGGANCIMFVLCTRVV
tr|A0A7K7CGN5|A0A7K7CGN5_APHCE GAVLV----SCAL----SCVGSALLLCTHAQLLLYLSLADLLSALSYFYGVLVLQGALSTFANTSSFFWTMAIALLLWC-FHAVSWGVPLGITVAAVAL----LLWML-LTGKVWEILAYVTLPVLYILILIPVIFIFLRIWSTVRFILVLVVLH-GIGN-TFQGGANCIMFVLCTRVV
tr|A0A851WQU0|A0A851WQU0_CORMO GAVLV----SCAL----SCVGSALLLCTHAQLLLYLSLADLLSALSYFYGVLVLQGALSTFANTSSFFWTMAIALLLWC-FHAVSWGLPLGITVAAVSL----LLWML-LTGKVWEILAYVTLPVLYILILIPVIFIFLRIWSTVRFILVLVVLH-GIGN-TFQGGANCIMFVLCTRVV
tr|K7FIF5|K7FIF5_PELSI         IIVLV----SCVL----SFLGASLLVFTHALLLLYLSLADLLSAASYFYGVLVLQGALSTFSNTSSFFWTMAIALLLCF-FHVMSWGVPLGITVAAVAL----ILWML-LAGKLWELLAYVILPVLYILILIPLIFIFLRIWSTVRFILVLVVLH-GIGN-TFQGGANCIMFVLCTRVI
tr|A0A8T1T6J7|A0A8T1T6J7_CHESE VIVLV----SCVL----SFLGSSLLIFTHALLLLYLSLADLLSAASYFYGVLELQGALSTFSNTSSFFWTTAIAFLLCF-FHVMSWGLPLGITVAAVSL----VLWML-LTGKLWELLAYVILPVLYILILIPLIFIFLRVWSTVRFILVLVVLH-GIGN-TFQGGANCIMFVLCTRVI
tr|A0A674JSW7|A0A674JSW7_TERCA VIVLV----SCVL----SFLGSSLLVFTHALLLLYLSLADLLSAVSYFYGVLVLQGALSTFSNTSSFFWTMAIAFLLCF-FHGMSWGVPLGITVAAVAL----VLWML-LTGKLWELLAYVILPVLYILILIPLIFIFLRIWSTIRFILVLVVLH-GIGN-TFQGGANCIMFVLCTRVI
tr|A0A8C3ICI0|A0A8C3ICI0_CHRPI VIVLV----SCVL----SFLGSSLLVFTHALLLLYLSLADLLSAVSYFYGVLVLQGALSTFSNTSSFFWTMAIAFLLCF-FHGMSWGLPLGITVAAVSL----VLWML-LTGKLWELLAYVILPVLYILILIPLIFIFLRIWSTIRFILVLVVLH-GIGN-TFQGGANCIMFVLCTRVI
tr|A0A8C0IXZ3|A0A8C0IXZ3_CHEAB VIVLL----SCAL----SFLGSSLLVFTHALLLLYLSLADLLSAASYFYGVLVLQGALSTFSNTSSFFWTTAIAFLLCF-FHGMSWGVPLGITVAAVAL----VLWML-LTGKLWELLAYVILPVLYILILIPLIFIFLRIWSTIRFILVLVVLH-GIGN-TFQGGANCIMFVLCTRAI
```

```
tr|A0A8C4WEI8|A0A8C4WEI8_9SAUR VIVLL----SCAL----SFLGSSLLVFTHALLLLYLSLADLLSAVSYFYGVLVLQGALSTFSNTSSFFWTMAIAFLLCF-FHGMSWGLPLGITVAAVSL----VLWML-LTGKLWELLAYVILPVLYILILIPLIFIFLRIWSTIRFILVLVVLH-GIGN-TFQGGANCIMFVLCTRVI
tr|A0A151N3W3|A0A151N3W3_ALLMI ITVLS----SCAL----SFLGSSLLVCTHALLLLYLSLADLLSAASYFYGVLVLQGALSTFSNTSSFFWTMAIAILLCF-FHVVSWGVPLGITVAAVAL----VLWML-LAGKIWEILAYLILPLLYILILIPIIFIFLRIWSTVRFILLLVVLH-GIGN-TFQGGANCIMFVLCTRVT
tr|A0A7M4E4T9|A0A7M4E4T9_CROPO VTVLG----SCAL----SFLGSGLLVCTHALLLLYLSLADLLSAASYFYGVLVLQGALSTFSNTSSFFWTMAIAILLCF-FHAVSWGLPLGITVAAVSL----VLWML-LAGKIWEILAYLILPVLYILILIPIIFIFLRIWSTVRFFLLLVVLH-GIGN-TFQGGANCIMFVLCTRVV
tr|A0A1U7S680|A0A1U7S680_ALLSI ITVLS----SCAL----SFLGSSLLVCTHALLLLYLSLADLLSAASYFYGVLVLQGALSTFSNTSSFFWTMAIAILLCF-FHAVSWGVPLGITVAAVAL----VLWML-LAGKIWEILAYLILPLLYILILIPIIFIFLRIWSTVRFILLLVVLH-GIGN-TFQGGANCIMFVLCTRVV
tr|A0A3Q0FP08|A0A3Q0FP08_ALLSI ITVLS----SCAL----SFLGSSLLVCTHALLLLYLSLADLLSAASYFYGVLVLQGALSTFSNTSSFFWTMAIAILLCF-FHAVSWGLPLGITVAAVSL----VLWML-LAGKIWEILAYLILPLLYILILIPIIFIFLRIWSTVRFILLLVVLH-GIGN-TFQGGANCIMFVLCTRVV
                                                               *:  :: :* ..:           *  : :   :**:** ::*.          *  ..               .:*  : .  *       :. :      *  ::  . :.*:.    *  *
```

Supplemental Figure S1: Multiple sequence alignment of proteins with “GPR157” in the annotation, aligned with AtGCR1 (top row), performed with MUSCLE software (Edgar RC. 2004). Helix boundaries were predicted for a few representative sequences using DeepTMHMM program (Hallgren et al. 2022). Only the helices are included in the alignment for clarity. The amino acid residues are coloured according to the helices they belong to, Red-helix1, Blue-helix2, Green-helix3, Purple-helix4, Orange-helix5, Brown-helix6, Magenta-helix7. The conserved positions are shown in the last row. The fully conserved residues are indicated by *, residues with strongly similar properties are indicated by :, and residues with weakly similar properties are indicated by ·.

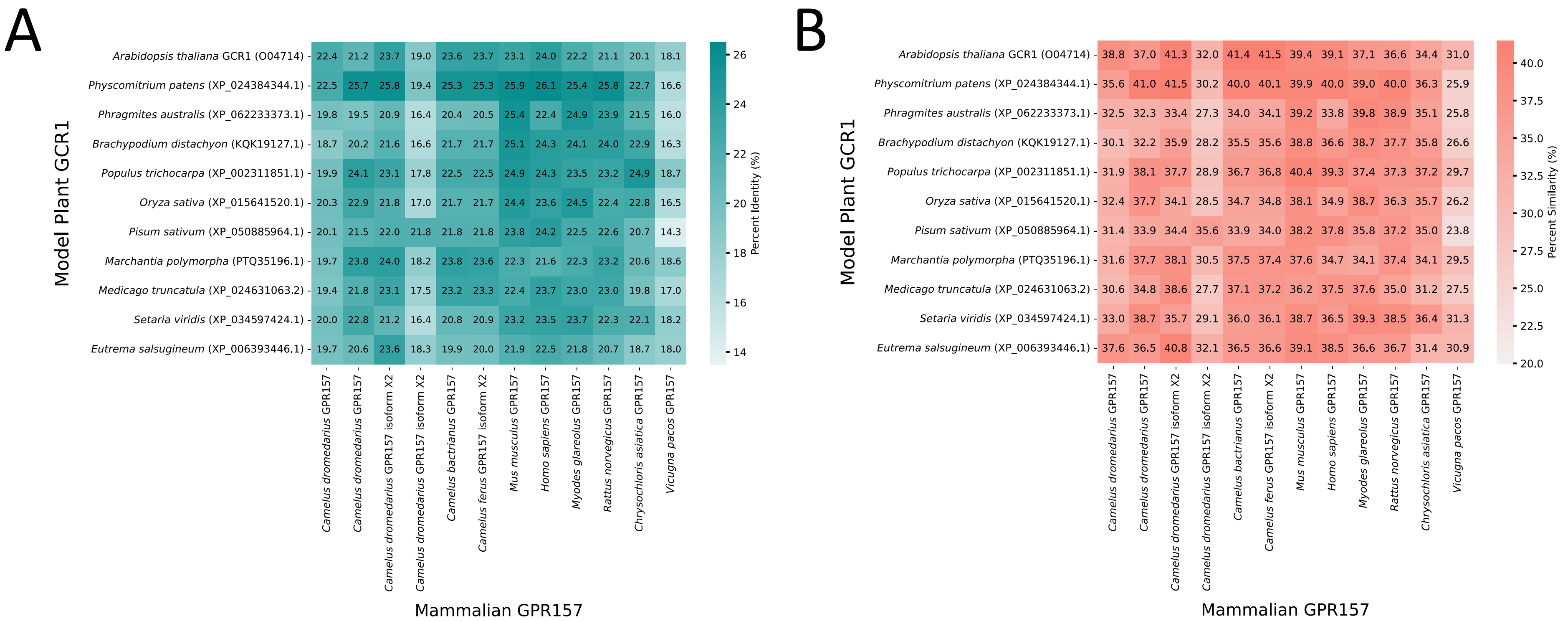

Supplemental Figure S2: Matrices comparing the: A) percent identity and B) percent similarity values between AtGCR1 orthologs from a broad selection of plant species vs. mammalian GPR157 protein sequences. Aside from the reference AtGCR1 sequence in the top row, plant AtGCR1 orthologs were ordered based on the maximum value in the row of identities, and the same order was maintained for the similarity matrix. Mammalian GPR157 sequences on the x-axis are listed in the same order as in Supplemental Table S1-B. Accession IDs of all plant GCR1 protein sequences are provided.

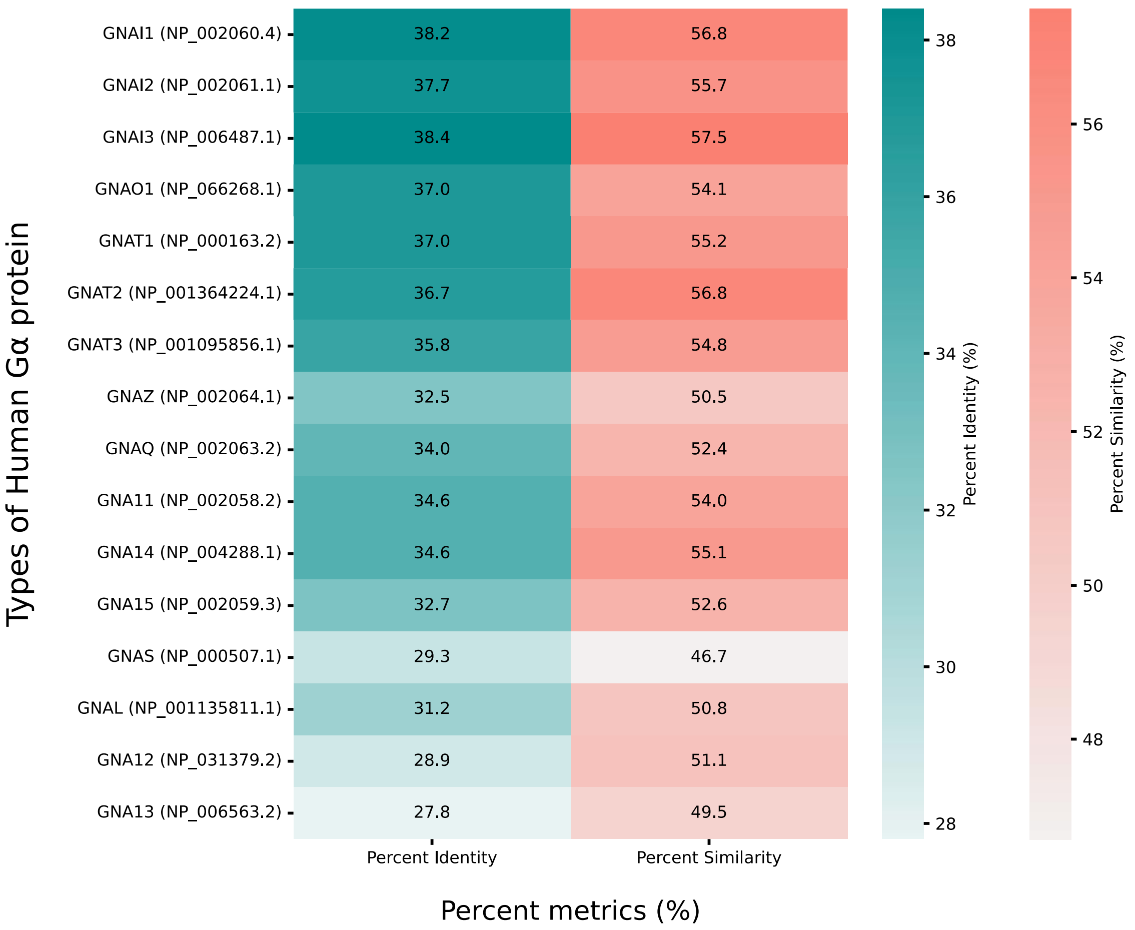

Supplemental Figure S3: Matrix with background color coding and enumeration of percent identity (cyan) and percent similarity (salmon) values between GPA1 of *Arabidopsis thaliana* and the canonical isoforms of each of the 16 human Gα proteins. Human Gα proteins are grouped into four families: Gαi (GNAI1, GNAI2, GNAI3, GNAO1, GNAT1, GNAT2, GNAT3 and GNAZ), Gαq (GNAQ, GNA11, GNA14 and GNA15), Gαs (GNAS and GNAL), and Gα12/13 (GNA12 and GNA13). GPA1 displays the highest identity & similarity with the Gαi and Gαq families of human Gα subunits.

Supplemental Materials and Methods

Our existing pDOE vector system (Gookin and Assmann, 2014) was adapted for transient split luciferase assays by splitting firefly luciferase into nLuc (residues 1-398) and cLuc (residues 399-550) fragments. nLuc DNA sequence fused at the 5' end with the existing pDOE MCS1 sequence and a 3' HiBit tag sequence (i.e. to create a X-nLuc-HiBit cassette) was ordered as a gBlock (IDT) as was the cLuc sequence adapted with the existing pDOE MCS3 sequence (i.e. to create a X-cLuc cassette). The nVenus fragment of pDOE-04 was excised between XhoI/XbaI restriction sites and replaced with X-nLuc-HiBit. The cVenus fragment was then excised from this preliminary vector, between Bsu36I/AsiSI restriction sites, and replaced with X-cLuc. This yielded a single vector with an X-nLuc-HiBit + X-cLuc (pSLU-01) tag configuration for use in this study. Finally, a NOS promoter::Renilla luciferase cassette was cloned into EcoRI/KpnI sites of MCS2, such that normalization of reconstituted firefly luciferase signal would be possible by either Renilla luciferase activity, or by using the HiBit tag attached to the nLuc fusion protein, which can be assayed using a Nano-Glo HiBit Lytic detection system.

GPA1 was first cloned into the NcoI/SpeI restriction sites of pSLU-01 MCS1, yielding a GPA1-nLuc-HiBit + X-cLuc parent vector for use as a negative control. *AtGCR1* was then cloned into the KflI/AatII restriction sites of MCS3 of the parent vector, to yield the test vector encoding GPA1-nLuc-HiBit + AtGCR1-cLuc. An internal NcoI restriction site was synonymously replaced from the Arabidopsis *RGS1* coding region by REPLACR mutagenesis (Trehan et al. 2016) and the resultant coding region was cloned into the MCS3 KflI/AatII restriction sites of pSLU-01, using an RsrII restriction site at the 5' end of the coding region that is compatible with the KflI site in pSLU-01. *GPA1* was subsequently cloned into the NcoI/SpeI of pSLU-01-RGS1 MCS1 to yield the positive control vector encoding GPA1-nLuc-HiBit + RGS1-cLuc. Constructs were Agroinfiltrated into *Nicotiana benthamiana* leaves from at least two plants at an OD_{600} of 0.1 in 10 mM MES pH 5.6, 10 mM $MgSO_4$ and 150 µM acetosyringone buffer. After 40-42 hours 12 leaf discs per construct were excised from the infiltration zones using a standard 0.25 inch paper hole punch and placed in individual wells of a white polystyrene 96 well plate (Corning product #3912). 100 µl of 0.3 mM D-luciferin (GoldBio product # LUCK-100) in water was added to each well and briefly vacuum infiltrated into the leaf discs. Luminescence of firefly luciferase was then monitored with a 5 second integration time per well and a gain of 200 in a Synergy Neo2 multimode reader (Biotek) until peak luminescence was reached in all wells. The D-luciferin substrate was then removed and replaced with 80 µl of Nano-Glo HiBit Lytic detection mix (Promega product # N3040). Nanoluc luminescence was then monitored as above. The resultant NanoLuc fluorescence gives an estimate of relative HiBit abundance, and therefore of GPA1-nLuc-HiBit expression. Peak firefly luciferase luminescence was therefore normalized to peak Nanoluc luminescence for each sample to yield a relative luminescence value. Data are plotted as the average (±SEM) of relative luminescence values. Statistical significance was determined by Student's *t* test.

A 3D structural model of AtGCR1 coupled with GPA1 was constructed using the AlphaFold 3 server (Abramson et al. 2024). Distances between the two chains (AtGCR1 and GPA1) of the complex were calculated with a TCL (Tool Command Language) script executed in Visual Molecular Dynamics (VMD) (Humphrey et al. 1996). Any two residues less than a minimum distance of 8 Å apart (for the Cβ atoms), were considered as interacting pairs. The interacting pairs of residues were subsequently mapped and categorized based on the secondary structural elements (SSEs) they reside within. A network illustrating the interactions between AtGCR1 and GPA1 SSEs was derived using the protocol of Matic et al. (2023)

and visualized using Cytoscape (Shannon et al. 2003). Nodes in the network represent contact elements, with node size proportional to the sum total of interactions an element had with all other elements. Edges connecting the nodes indicate interactions between SSEs, with edge width corresponding to the number of interacting residues between that pair of nodes.

The consensus sequence of GPR157 was obtained by submitting the multiple sequence alignment of 400 GPR157 protein sequences to the EMBOSS Cons tool (Madeira et al. 2024) tool. Pairwise alignment of AtGCR1 with the consensus sequence of GPR157 was performed using EMBOSS NEEDLE tool (Madeira et al. 2024), the Gap open penalty and Gap extend penalty were chosen as default.

To compare AtGCR1 with other GPRs, a query was performed at UniprotKB (Uniprot Consortium, 2023) with "GPR*" as gene name and "Mammalia" as taxonomy (QUERY- "(gene:GPR*) AND (taxonomy_id:40674)"). A total of 11,691 protein sequence results were obtained, out of which 500 were reviewed entries whereas 11,191 were unreviewed entries. All 11,691 protein sequences were downloaded and named as GPR-Mammal dataset. A BLASTp (Altschul et al. 1990) comparison was performed using AtGCR1 as the query sequence and the protein sequences of GPR-Mammal dataset using a default "E-value: Expect threshold" as 0.05. A total of only 123 entries was retained in the BLASTp output. Out of these 123 results, 108 entries (including all of the top 60) are of GPR157 sequences of various mammals. Only 15 entries out of 123 are of five other unique GPRs. To assess complete lengthwise similarities, pairwise global alignments of the five unique GPRs with AtGCR1 were computed using the EMBOSS NEEDLE tool (Madeira F et al. 2024).

To compare homologs of AtGCR1 in other plant species, we chose ten model plant species based on Cesarino et al. (2020). All the plant species chosen had at least two chromosome level genome assemblies. The GCR1 protein sequences were compared to all the mammalian GPR157 sequences obtained from BLASTp results using global alignment implemented in the EMBOSS NEEDLE tool (Madeira F et al. 2024). In cases where a plant species had more than one GCR1 protein sequence, the sequence with the lowest E-value from BLASTp was used for the analysis.

To compare GPA1 with mammalian Gα subunits, sequences of canonical isoforms corresponding to the 16 human Gα subunits were retrieved from Uniprot and the EMBOSS NEEDLE tool (Madeira et al., 2024) was used to perform pairwise alignment of each with the GPA1 sequence.

Supplemental Materials and Methods References:

Abramson J, Adler J, Dunger J. et al. Accurate structure prediction of biomolecular interactions with AlphaFold 3. Nature. 2024:630:493-500. https://doi.org/10.1038/s41586-024-07487-w

Altschul SF, Gish W, Miller W, Myers EW, Lipman, DJ. Basic local alignment search tool. J Mol Biol. 1990:215(3):403-410. https://doi.org/10.1016/S0022-2836(05)80360-2

Cesarino I, Dello Ioio R, Kirschner GK, Ogden MS, Picard KL, Rast-Somssich MI, Somssich M. Plant science's next top models. Ann Bot. 2020: 126(1):1-23. https://doi.org/10.1093/aob/mcaa063

Edgar RC. MUSCLE: multiple sequence alignment with high accuracy and high throughput. Nucleic Acids Res. 2004:32(5):1792-1797. https://doi.org/10.1093/nar/gkh340

Gookin TE, Assmann SM. Significant reduction of BiFC non-specific assembly facilitates in planta assessment of heterotrimeric G-protein interactors. Plant J. 2014:80(3):553-567. https://doi.org/10.1111/tpj.12639

Hallgren J, Tsirigos KD, Pedersen MD, Almagro Armenteros JJ, Marcatili P, Nielsen H, Krogh A, Winther O. DeepTMHMM predicts alpha and beta transmembrane proteins using deep neural networks. bioRxiv [preprint]. 2022.04.08.487609. https://doi.org/10.1101/2022.04.08.487609

Humphrey W, Dalke A, Schulten K. VMD - Visual Molecular Dynamics. J Mol Graphics. 1996:14:33-38. https://doi.org/10.1016/0263-7855(96)00018-5

Madeira F, Madhusoodanan N, Lee J, Eusebi A, Niewielska A, Tivey ARN, Lopez R, Butcher S. The EMBL-EBI Job Dispatcher sequence analysis tools framework in 2024. Nucleic Acids Res. 2024:52(W1):W521-W525. https://doi.org/10.1093/nar/gkae241

Matic M, Miglionico P, Tatsumi M, Inoue A, Raimondi F. GPCRome-wide analysis of G-protein-coupling diversity using a computational biology approach. Nat Commun. 2023:14:4361. https://doi.org/10.1038/s41467-023-40045-y

Shannon P, Markiel A, Ozier O, Baliga NS, Wang JT, Ramage D, Amin N, Schwikowski B, Ideker T. Cytoscape: a software environment for integrated models of biomolecular interaction networks. Genome Res. 2003:13(11):2498-2504. https://doi.org/10.1101/gr.1239303

Trehan A, Kiełbus M, Czapinski J, Stepulak A, Huhtaniemi I, Rivero-Müller A. REPLACR-mutagenesis, a one-step method for site-directed mutagenesis by recombineering. Sci Rep. 2016:6:19121. https://doi.org/10.1038/srep19121

UniProt Consortium. UniProt: the Universal Protein Knowledgebase in 2023. Nucleic Acids Res. 2023:51:D523-D531. https://doi.org/10.1093/nar/gkac1052
